# Global, regional, and national disease burden and attributable risk factors of HIV/AIDS in older adults aged 70 years and above: a trend analysis based on the Global Burden of Disease study 2019

**DOI:** 10.1017/S0950268823001954

**Published:** 2023-12-15

**Authors:** Min Du, Min Liu, Jue Liu

**Affiliations:** 1 Department of Epidemiology and Biostatistics, School of Public Health, Peking University, Beijing, China; 2 Institute for Global Health and Development, Peking University, Beijing, China; 3 Global Center for Infectious Disease and Policy Research & Global Health and Infectious Diseases Group, Peking University, Beijing, China; 4 Key Laboratory of Epidemiology of Major Diseases (Peking University), Ministry of Education, Beijing, China

**Keywords:** HIV/AIDS, incidence, mortality, older adults, prevalence

## Abstract

We aimed to assess the burden and trend of the HIV/AIDS epidemic among older adults over the past three decades at different geographical levels, based on the data collected from the Global Burden of Diseases (GBD) study 2019. This assessment identified the average annual percentage changes (AAPCs) using Joinpoint regression analysis. Globally, the incidence of HIV/AIDS has decreased (AAPC = −3.107); however, the overall prevalence has consistently increased (AAPC = 5.557). Additionally, both mortality (AAPC = 2.166) and disability-adjusted life years (DALYs; AAPC = 2.429) have increased. The highest increasing trends in female HIV/AIDS incidence and prevalence were observed in the Central Asia region. However, for males, these trends were observed in the Oceania region and the high-income Asia Pacific region, respectively. In recent decades, females aged 70–74 years had the highest incidence and prevalence, while males aged 70–74 years had highest mortality and DALYs in low social development index (SDI) regions. Unsafe sex resulted in 15 381.16 deaths, accounting for 90.73% of all HIV/AIDS deaths, and 331 140.56 DALYs, accounting for 91.12% of all HIV/AIDS DALYs. The HIV/AIDS disease burden differs by region, age, and sex among older adults. Sexual health education and targeted screening for older adults are recommended.

## Introduction

Human immunodeficiency virus (HIV), a devastating infection, attacks the body’s immune system and weakens its ability to fight other infections, including tuberculosis and bacterial infections [1–7]. The World Health Organization (WHO) has reported that there were approximately 38.4 million people living with HIV (PLWH) at the end of 2021, and there were 1.5 million new HIV infections worldwide in 2021 [8]. In order to curb the HIV epidemic, it is proposed that each country should achieve three goals by 2030, known as the ‘95–95–95’ targets: 95% of HIV-positive individuals should know their status, 95% of those who know their HIV-positive status should be on treatment, and 95% of those on treatment should have viral suppression [9]. However, these goals still pose significant challenges. In Europe and Central Asia, one out of every five PLWH was unaware of their status, and only 43% of PLWH had a low level of viral loads that could prevent transmission [10]. The goal of ending the HIV epidemic is still far away [11].

Aging is associated with a higher burden of diseases, including cognitive impairment, chronic diseases, and accidental injuries. The number and proportion of people aged 60 years and above are both increasing. According to the WHO report, the number of people aged 60 years and above was 1 billion in 2019, and it is projected to increase to 1.4 billion by 2030 and 2.1 billion by 2050 [12]. HIV infections have significant implications for the long-term health and well-being of older people. Older PLWH are often at a greater risk of infectious diseases, such as tuberculosis [13]. No doubt, HIV/acquired immunodeficiency syndrome (AIDS) infections among older people are significant public health issues. This is due to the increasing aging population, the substantial economic burden, poverty, and isolation [13]. The number of HIV-infected older adults has also grown from 2004 to 2015, with significant increases at an annual average change of 2.1% in Europe [14]. The Joint United Nations Programme on HIV/AIDS (UNAIDS) has reported that an estimated 3.6 million people aged 50 years and above were living with HIV worldwide [15]. In 2012, the proportion of older PLWH has increased in all regions, albeit at different rates. Since 2007, the rate was 10% in low- and middle-income countries, and approximately 30% in high-income countries [15].

However, older adults infected with HIV were largely neglected by the prevention community, partly due to a lack of data regarding their sexual behaviour and the emphasis on younger individuals [13]. Since the widespread use of antiretroviral therapy (ART) and pre-exposure prophylaxis, life expectancy has increased over time. This has led to a rapidly increasing number of HIV-infected older adults, with regional differences [16]. At the same time, it has commonly been assumed that older adults are not at risk of acquiring HIV. However, HIV acquisition risk factors, such as divorce or death of a partner, as well as lack of HIV knowledge and failure to adopt preventive behaviours may contribute to changes in the HIV/AIDS epidemic status [17]. However, few HIV strategies currently address the HIV epidemic in older populations due to a lack of data. Data collection for HIV incidence and prevalence among older adults is often omitted [13]. Thus, data on the burden, trend, and attributable risk factors of HIV/AIDS among older adults are sparse but critical. In this study, we utilized the Global Burden of Diseases (GBD) study 2019 to evaluate the burden and trend of the HIV/AIDS epidemic among older adults over the past three decades on a global, regional, and national scale. We also explored the associated risk factors to establish a solid basis for the development and implementation of future strategies for HIV prevention, treatment, and care.

## Methods

### Data source

The GBD study is coordinated by the Institute for Health Metrics and Evaluation at the University of Washington, USA. It is a systematic and scientific effort to quantify the comparative magnitude of health losses due to diseases by sex, age, region, and country over time [18]. From the Global Health Data Exchange website, established by the GBD group, we obtained data on the annual number, incidence, prevalence, mortality, and disability-adjusted life years (DALYs) of HIV/AIDS by sex, age, region, and country in adults aged 70 years and above from 1990 to 2019 on 14 November 2022 [18]. Detailed descriptions of the methods and approach of GBD 2019 have been previously reported [19]. Series models were used to estimate HIV/AIDS disease burden based on the data from published studies, governmental agencies, the WHO, and the Pan American Health Organization, among others. The underreporting adjustment factors were estimated using a regularized and trimmed meta-regression Bayesian (MR-BRT) approach. The uncertainty from MR-BRT was applied to the adjustment for age and sex. Once the data were adjusted for underreporting, a hybrid approach was used to generate estimates by employing two models: (1) a space–time Gaussian process regression and (2) a negative binomial regression using fixed effects to model all incidence [19].

The burden of disease attributable to risk factors involves a counterfactual approach that quantifies the level of exposure to the risk factor in a population. Risk factors in GBD are part of a comparative risk assessment framework and are modelled independently through six analytical steps [20, 21]. GBD 2019 has identified risk factors for HIV/AIDS, namely drug use, unsafe sex, and intimate partner violence (IPV). We extracted population-attributable fractions (PAFs), attributable deaths, and DALYs. The PAF quantifies the contribution of a risk factor to the occurrence of a disease or death. It represents the proportion of outcomes that would be reduced in a given year if exposure to a risk factor in the past had been reduced to the counterfactual level of theoretical minimum risk exposure. For a given risk–outcome pair, the number of attributable deaths/DALYs was estimated by multiplying the total number of deaths or DALYs for the outcome by the PAF for the risk–outcome pair, taking into account age, sex, location, and year [20, 22].

### Regions and demographics

All 204 countries or territories were classified into five social development index (SDI) regions, including low, low-middle, middle, high-middle, and high SDI regions. The SDI was developed by GBD researchers and is a composite indicator of the total fertility rate under age 25 years, years of education for those aged 15 and above, and lag-distributed income per capita [23]. Meanwhile, 204 countries or territories were also separated into 21 regions based on their epidemiological homogeneity and geographical contiguity, including high-income Asia Pacific, Central Asia, and others in the GBD study [24]. We classified 204 countries or territories into six WHO regions and four World Bank income regions. In this study, we stratified all data by age groups (70–74, 75–80, 80–84, 85–90, 90–94, and 95+ years), sex, region, and country level.

### Statistical analysis

The epidemic status was presented using absolute number with 95% uncertainty intervals (UIs) and age-standardized rate (ASR) with 95% UIs [25]. We calculated the relative changes from 1990 to 2019, which were defined as follows: 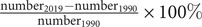 . Average annual percentage change (AAPC) as a commonly used measure of the ASR trend over a specific time period was calculated using Joinpoint regression analysis [22, 26, 27]. Joinpoint regression analysis assessed the trends of the incidence, prevalence, mortality, and DALYs over the time period using a logarithmic scale. In this study, the number of joinpoints ranged from 0 to 5. Joinpoint regression analysis tested each group for significance using a Monte Carlo permutation method, selected the best appropriate model, and estimated AAPC with a 95% confidential interval, which provides a single summary measure for the trend over the past 30 years [27, 28]. The model also identified the year with significant changes in trends. Joinpoint regression analyses were performed using the Joinpoint regression programme (version 4.9.1.0, November 2022; Statistical Methodology and Applications Branch, Surveillance Research Program, National Cancer Institute). Other analysis was conducted using R (version 4.1.0). Ethical approval and patient consent were not required due to the availability of public data from GBD.

## Results

### Trends in HIV/AIDS incidence from 1990 to 2019 at the global, regional, national, sex, and age levels

**Figure 1.**
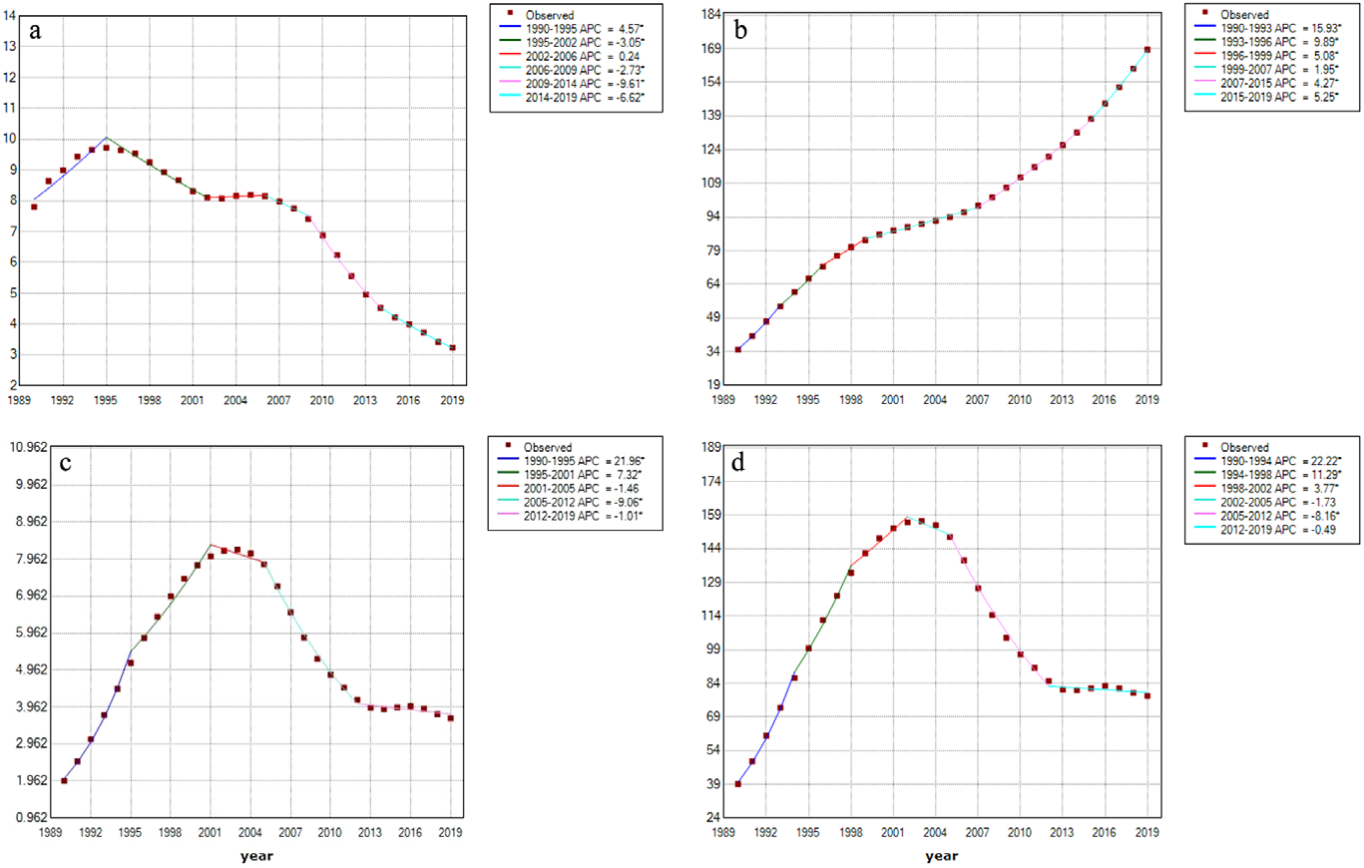
Global trends in the incidence, prevalence, mortality, and DALYs of HIV/AIDS from 1990 to 2019. (a) Incidence; (b) prevalence; (c) mortality; (d) DALYs. APC, annual percentage change; DALYs, disability adjusted life years

**Figure 2.**
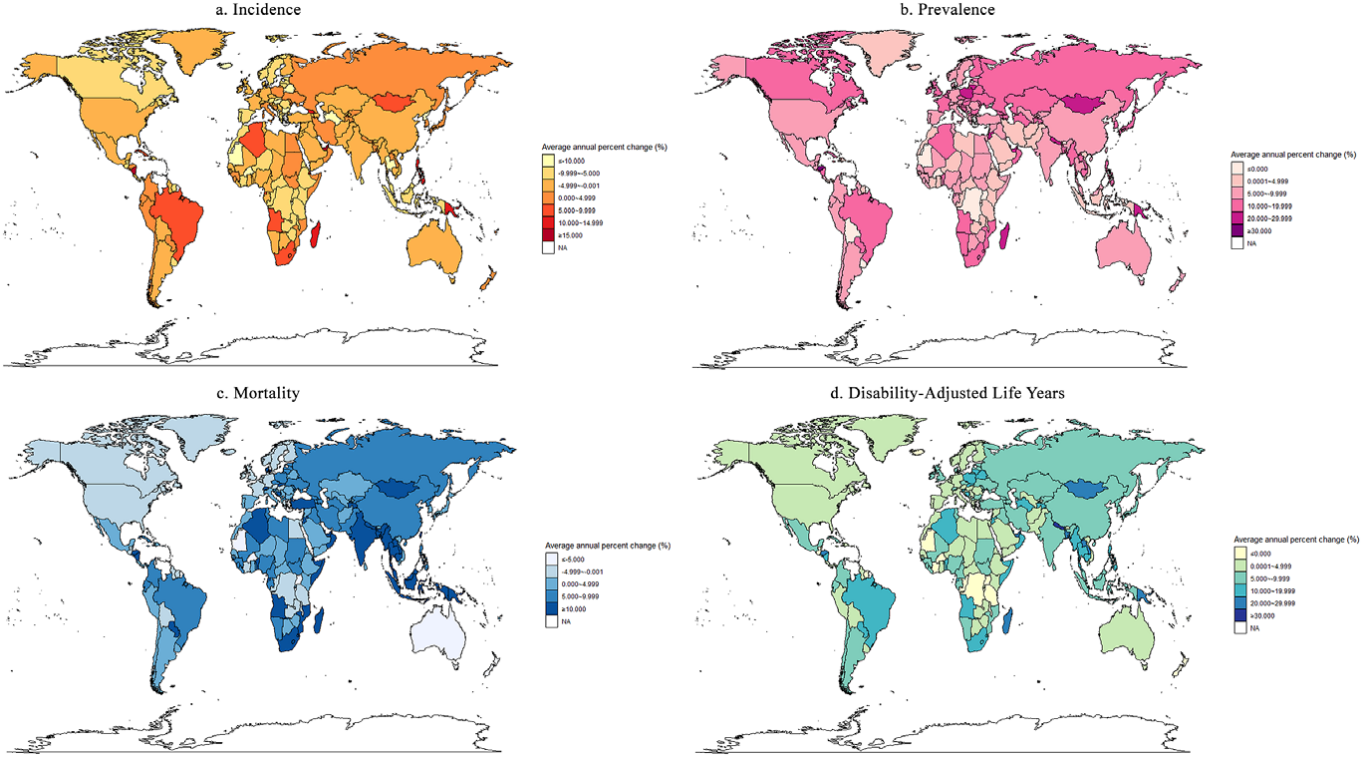
Trends in the incidence, prevalence, mortality, and DALYs of HIV/AIDS in 204 countries and territories from 1990 to 2019. (a) Incidence; (b) prevalence; (c) mortality; (d) DALYs. AAPC, average annual percentage change; DALYs, disability adjusted life years

The overall incidence declined from 7.80 (95% UI 6.30, 9.62) per 100 000 population in 1990 to 3.24 (95% UI 2.66, 3.97) in 2019 (AAPC = −3.107, 95% CI −3.487, −2.719) (Table 1, Supplementary Table S1, and Figure 1a). The incidence was highest in the low SDI region and the southern Sub-Saharan Africa region in 2019. Middle SDI region experienced the highest increase in incidence from 1990 to 2019 (AAPC = 1.175, 95% CI 0.308, 2.049). Notably, although most regions had a decreasing trend, the AAPC was greater than 5% in Oceania (AAPC = 7.625, 95% CI 5.438, 9.858) and the tropical Latin America region (AAPC = 5.423, 95% CI 4.850, 6.000). There were 17, 156 and 31 countries or territories that had a decreasing, increasing, or stable trend of HIV/AIDS incidence with AAPC ranging from −6.935 to 37.496 from 1990 to 2019 (Figure 2a and Supplementary Table S2).

**Table 1.** HIV/AIDS incidence and prevalence among older adults in 1990 and 2019, and their temporal trends from 1990 to 2019

	Age-standardized incidence rate (per 100 000 population)			Age-standardized prevalence rate (per 100 000 population)		
	1990 (95% UI)	2019 (95% UI)	AAPC (95% CI)	1990 (95% UI)	2019 (95% UI)	AAPC (95% CI)
Global	7.80 (6.30, 9.62)	3.24 (2.66, 3.97)	−3.104 (−3.487, −2.719)	34.88 (30.00, 41.58)	168.62 (146.01, 193.23)	5.557 (5.469, 5.645)
SDI region						
Low SDI	81.16 (58.10, 107.70)	14.90 (10.68, 20.48)	−5.712 (−5.867, −5.556)	397.75 (320.99, 485.21)	616.91 (521.36, 737.77)	1.462 (1.257, 1.667)
Low-middle SDI	19.68 (14.46, 27.07)	4.79 (3.67, 6.25)	−4.886 (−5.230, −4.540)	59.66 (46.48, 82.06)	214.08 (187.17, 250.99)	4.407 (4.088, 4.726)
Middle SDI	4.17 (3.09, 6.84)	5.61 (4.49, 7.01)	1.175 (0.308, 2.049)	15.94 (11.38, 26.75)	230.52 (202.48, 260.33)	9.668 (9.437, 9.900)
High-middle SDI	0.81 (0.53, 1.39)	0.52 (0.37, 0.69)	−1.405 (−2.782, −0.008)	4.71 (3.42, 6.83)	31.34 (28.06, 35.08)	6.770 (6.579, 6.961)
High SDI	0.87 (0.62, 1.26)	0.55 (0.25, 0.90)	−1.517 (−3.052, 0.043)	10.57 (6.98, 16.01)	132.45 (75.11, 192.40)	9.130 (9.001, 9.259)
GBD region						
Andean Latin America	2.54 (1.25, 6.37)	2.83 (0.99, 5.46)	0.753 (−0.099, 1.612)	24.85 (13.41, 58.54)	113.99 (60.49, 171.63)	5.511 (5.051, 5.973)
Australasia	0.53 (0.32, 0.75)	0.30 (0.16, 0.50)	−1.886 (−3.291, −0.460)	5.52 (4.05, 7.55)	52.37 (39.31, 63.23)	8.112 (7.912, 8.314)
Caribbean	26.19 (17.80, 39.95)	4.88 (2.75, 8.06)	−5.801 (−6.629, −4.965)	85.16 (53.86, 133.25)	220.45 (177.95, 271.14)	3.294 (2.936, 3.653)
Central Asia	0.07 (0.03, 0.12)	0.09 (0.03, 0.19)	1.167 (0.327, 2.014)	0.42 (0.22, 1.05)	5.43 (3.60, 7.39)	9.509 (9.107, 9.913)
Central Europe	0.09 (0.06, 0.13)	0.04 (0.02, 0.07)	−2.987 (−4.791, −1.149)	0.44 (0.30, 0.66)	10.36 (6.50, 14.70)	11.492 (11.021, 11.966)
Central Latin America	3.06 (2.17, 5.08)	1.84 (0.88, 2.92)	−1.320 (−2.239, −0.391)	17.47 (9.66, 35.72)	104.82 (68.36, 136.19)	6.495 (6.200, 6.790)
Central Sub-Saharan Africa	111.50 (75.21, 156.56)	23.00 (14.74, 33.81)	−5.319 (−5.480, −5.157)	460.22 (315.85, 660.16)	570.78 (436.01, 748.44)	0.693 (0.457, 0.930)
East Asia	1.70 (0.80, 4.43)	1.39 (0.66, 2.06)	−1.635 (−4.171, 0.968)	10.90 (6.92, 25.37)	54.96 (26.33, 99.07)	5.722 (5.279, 6.168)
Eastern Europe	0.05 (0.03, 0.07)	0.13 (0.02, 0.31)	4.023 (−0.201, 8.426)	0.26 (0.17, 0.42)	9.82 (6.21, 14.15)	13.450 (12.855, 14.048)
Eastern Sub-Saharan Africa	252.61 (185.71, 326.42)	47.59 (34.49, 65.31)	−5.634 (−5.829, −5.439)	1 131.69 (918.70, 1 368.65)	2 169.36 (1839.86, 2 602.40)	2.207 (2.000, 2.413)
High-income Asia Pacific	0.26 (0.13, 0.47)	0.27 (0.10, 0.59)	−0.803 (−3.347, 1.808)	0.88 (0.42, 1.54)	43.52 (23.06, 64.29)	14.484 (14.100, 14.870)
High-income North America	1.51 (0.81, 2.14)	1.09 (0.19, 2.10)	−0.689 (−2.506, 1.162)	22.65 (14.43, 34.77)	292.67 (151.54, 450.37)	9.207 (8.964, 9.452)
North Africa and Middle East	0.69 (0.15, 2.09)	0.96 (0.30, 3.21)	0.562 (−0.119, 1.247)	3.66 (1.70, 9.98)	16.09 (7.72, 38.38)	5.232 (5.101, 5.363)
Oceania	2.21 (0.43, 9.76)	19.57 (0.46, 70.12)	7.625 (5.438, 9.858)	11.34 (2.84, 44.97)	374.26 (19.54, 1 005.00)	12.940 (12.027, 13.861)
South Asia	0.94 (0.32, 3.00)	0.48 (0.12, 1.11)	−2.301 (−3.073, −1.524)	4.89 (2.50, 14.76)	25.65 (17.66, 37.65)	5.936 (5.697, 6.174)
Southeast Asia	5.02 (1.93, 13.56)	1.12 (0.56, 2.07)	−5.164 (−6.972, −3.321)	11.72 (5.60, 28.40)	134.36 (88.16, 196.79)	8.425 (8.089, 8.762)
Southern Latin America	3.67 (1.79, 9.34)	1.25 (0.84, 1.73)	−3.545 (−5.013, −2.053)	25.42 (16.85, 38.39)	97.59 (83.77, 111.61)	4.817 (4.646, 4.989)
Southern Sub-Saharan Africa	145.40 (66.36, 254.50)	193.40 (149.77, 251.03)	0.998 (−0.002, 2.008)	360.64 (193.63, 685.04)	7 812.31 (6 880.84, 8 914.86)	11.144 (10.701, 11.588)
Tropical Latin America	1.08 (0.85, 1.39)	4.88 (2.35, 7.90)	5.423 (4.850, 6.000)	6.67 (4.43, 11.01)	213.40 (128.56, 309.42)	12.736 (12.249, 13.225)
Western Europe	0.66 (0.46, 1.08)	0.31 (0.21, 0.51)	−2.549 (−3.038, −2.057)	5.44 (3.56, 8.51)	69.17 (50.75, 84.25)	9.173 (8.836, 9.512)
Western Sub-Saharan Africa	52.34 (35.45, 75.22)	22.76 (18.23, 28.77)	−2.896 (−3.297, −2.493)	207.65 (154.53, 294.63)	707.67 (588.47, 871.61)	4.239 (3.743, 4.737)
WHO region						
African region	130.87 (96.44, 170.14)	51.17 (41.02, 63.74)	−3.242 (−3.439, −3.044)	532.98 (439.47, 634.75)	2036.83 (1800.85, 2 342.77)	4.681 (4.478, 4.884)
Eastern Mediterranean region	1.06 (0.20, 3.20)	1.32 (0.44, 3.96)	0.678 (−0.369, 1.737)	8.02 (3.06, 23.56)	21.03 (9.38, 52.25)	3.438 (3.281, 3.596)
European region	0.41 (0.29, 0.66)	0.22 (0.15, 0.35)	−2.176 (−2.970, −1.375)	3.31 (2.22, 5.08)	45.27 (34.10, 54.70)	9.459 (9.314, 9.604)
Region of the Americas	2.69 (2.11, 3.41)	2.09 (1.15, 2.97)	−0.725 (−1.315, −0.132)	22.56 (16.23, 31.77)	225.96 (148.26, 307.69)	8.296 (8.216, 8.376)
South-East Asia region	2.55 (0.97, 6.83)	0.63 (0.27, 1.23)	−4.872 (−5.668, −4.071)	6.52 (3.41, 15.94)	59.17 (39.71, 82.67)	7.772 (7.343, 8.202)
Western Pacific region	1.36 (0.70, 3.31)	1.14 (0.60, 1.59)	−1.092 (−2.828, 0.675)	8.51 (5.46, 18.78)	53.19 (32.75, 83.60)	6.513 (6.001, 7.028)
World Bank income region						
Low income	132.31 (89.81, 180.80)	24.31 (17.37, 33.69)	−5.714 (−5.883, −5.544)	627.65 (497.25, 770.45)	1 080.02 (920.22, 1 295.18)	1.827 (1.563, 2.091)
Lower-middle income	11.18 (9.13, 14.01)	3.16 (2.42, 4.13)	−4.314 (−4.600, −4.027)	33.77 (27.56, 43.77)	122.40 (106.94, 141.93)	4.396 (4.083, 4.710)
Upper-middle income	2.62 (1.95, 4.38)	3.99 (3.16, 4.96)	1.520 (0.670, 2.378)	11.71 (8.18, 20.34)	171.82 (150.89, 193.71)	9.746 (9.583, 9.909)
High income	0.96 (0.69, 1.35)	0.54 (0.29, 0.85)	−1.879 (−3.094, −0.649)	10.21 (6.71, 15.48)	117.85 (69.73, 168.12)	8.821 (8.663, 8.980)

Note: AAPC, average annual percentage change; CI, confidence interval; UI, uncertainty interval.

**Figure 3.**
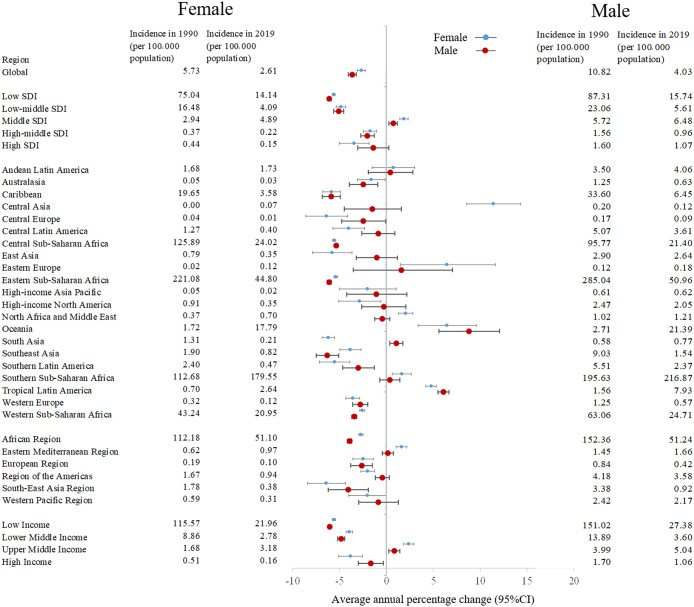
Incidence and trend of HIV/AIDS from 1990 to 2019 by global, SDI, GBD, WHO, and World Bank income regions. AAPC, average annual percentage changes; CI, confidence interval; GBD, Global Burden of Diseases; SDI, social development index; WHO, World Health Organization

In 1990 and 2019, although males had a higher HIV/AIDS incidence worldwide, they also had a higher decreasing trend of incidence (AAPC = −3.4435, 95% CI −3.800, −3.085), compared to females (AAPC = −2.668, 95% CI −3.083, −2.252) (Figure 3 and Supplementary Table S3). The region with the highest increasing trend of HIV/AIDS incidence among females was Central Asia (AAPC = 11.412, 95% CI 8.560, 14.338) (Figure 3 and Supplementary Table S3). For males, it was Oceania (AAPC = 8.401, 95% CI 4.424, 12.530). The characteristics of incident cases and changes with respect to sex and region were similar to the incidence (Supplementary Table S4). In 2019, among older adults aged 70 years and above, the 70–74 years age group had a higher incidence of HIV/AIDS in most regions (except Andean Latin America, Central Europe and East Asia). Additionally, this age group showed the greater number of regions (tropical Latin America, Central Asia, etc.) with an increasing trend in HIV/AIDS incidence, compared to the 75–79 years age group (Supplementary Figure S1A and Supplementary Table S5). In the recent decade, the incidence was higher among females aged 70–74 years than in other groups in low SDI regions from 1990 to 2019. This trend was different from the global and other four SDI regions (Supplementary Figure S2).

### Trends in the prevalence of HIV/AIDS from 1990 to 2019 at the global, regional, national, sex, and age levels

The overall prevalence increased from 34.88 (95% UI 30.00, 41.58) per 100 000 population in 1990 to 168.62 (95% UI 146.01, 193.23) in 2019 (AAPC = 5.557, 95% CI 5.469, 5.645) (Table 1, Supplementary Table S1, and Figure 1b). The prevalence was also highest in the low SDI region and the southern Sub-Saharan Africa region in 2019. The middle SDI region experienced the largest increase in prevalence from 1990 to 2019 (AAPC = 9.668, 95% CI 9.437, 9.900). The region with the highest increasing trend in prevalence was high-income Asia Pacific region (AAPC = 14.484, 95% CI 14.100, 14.870). There were 13, 185, and 6 countries or territories with a decreasing, increasing, or stable trend of HIV/AIDS prevalence with AAPC ranging from −6.019 to 32.225 from 1990 to 2019 (Figure 2b and Supplementary Table S6).

In low SDI regions, females had a higher prevalence of HIV/AIDS than males, contrasting most other regions (Supplementary Figure S3). The highest increasing trend of HIV/AIDS prevalence among females was observed in Central Asia (AAPC = 18.902, 95% CI 18.042, 19.768) (Supplementary Figure S3 and Supplementary Table S7). For males, it was in high-income Asia Pacific (AAPC = 13.701, 95% CI 13.356, 14.047). The characteristics of prevalent cases and changes with respect to sex and region were similar to the prevalence (Supplementary Table S8).

In 2019, the global prevalence of HIV/AIDS decreased with increasing age (Supplementary Figure S1B and Supplementary Table S9). However, the highest increasing trend of HIV/AIDS prevalence was observed in the 95+ years age group in most regions, except the high SDI region where it was observed in the 80–84 years age group. Although the global HIV/AIDS prevalence was highest in males aged 70–74 years from 1990 to 2019, in the recent decade, it has been highest in females aged 70–74 years in the other four SDI regions, except the high SDI region (Supplementary Figure S4).

### Trends and attributable risk factors of HIV/AIDS mortality from 1990 to 2019 at the global, regional, national, sex, and age levels

The estimated number of deaths due to HIV/AIDS has significantly increased from 3 954.08 (95% UI 2619.34, 5 545.47) in 1990 to 16 955.51 (95% UI 14 811.81, 19 876.02) in 2019, representing a 328.81% increase (Supplementary Table S10). The overall mortality increased from 1.96 (95% UI 1.30, 2.75) per 100 000 population in 1990 to 3.66 (95% UI 3.19, 4.29) in 2019 (AAPC = 2.166, 95% CI 1.462, 2.874) (Table 2 and Figure 1c). The middle SDI region experienced the largest increase in mortality from 1990 to 2019 (AAPC = 9.113, 95% CI 8.672, 9.557). Among the GBD regions, Oceania (AAPC = 16.871, 95% CI 15.750, 18.002) had the highest increasing trend in mortality. There were 25, 138, and 41 countries or territories with a decreasing, increasing, or stable trend of HIV/AIDS mortality (AAPC = −8.122 ~ 197.801) (Figure 2c and Supplementary Table S11).

**Table 2.** HIV/AIDS mortality and DALYs among older adults in 1990 and 2019, and their temporal trends from 1990 to 2019

	Age-standardized mortality rate (per 100 000 population)			Age-standardized DALYs (per 100 000 population)		
	1990 (95% UI)	2019 (95% UI)	AAPC (95% CI)	1990 (95% UI)	2019 (95% UI)	AAPC (95% CI)
Global	1.96 (1.30, 2.75)	3.66 (3.19, 4.29)	2.166 (1.462, 2.874)	39.12 (27.79, 52.49)	78.39 (67.60, 93.75)	2.429 (1.775, 3.087)
SDI region						
Low SDI	28.68 (18.47, 40.36)	16.59 (13.43, 21.24)	−2.084 (−2.723, −1.440)	561.49 (380.87, 766.15)	347.47 (280.12, 451.78)	−1.857 (−2.434, −1.278)
Low-middle SDI	2.69 (1.50, 4.43)	6.25 (5.16, 8.03)	2.754 (2.243, 3.268)	55.38 (34.23, 87.60)	130.11 (106.67, 167.48)	2.805 (2.386, 3.227)
Middle SDI	0.47 (0.29, 0.65)	6.22 (5.46, 6.98)	9.113 (8.672, 9.557)	10.39 (7.50, 13.55)	127.90 (111.77, 147.06)	8.991 (8.711, 9.272)
High-middle SDI	0.12 (0.10, 0.15)	0.67 (0.56, 0.78)	6.030 (5.357, 6.708)	2.57 (2.06, 3.09)	14.23 (11.74, 17.20)	6.095 (5.547, 6.645)
High SDI	0.34 (0.33, 0.34)	0.27 (0.24, 0.43)	−1.160 (−2.443, 0.141)	6.80 (6.13, 7.87)	14.67 (9.79, 21.80)	2.137 (1.047, 3.238)
GBD region						
Andean Latin America	1.51 (0.89, 3.11)	2.78 (1.49, 6.01)	2.193 (1.413, 2.980)	28.31 (17.38, 58.57)	56.88 (32.58, 125.78)	2.611 (1.870, 3.357)
Australasia	0.25 (0.24, 0.27)	0.06 (0.06, 0.06)	−5.300 (−6.421, −4.166)	4.88 (4.40, 5.60)	4.79 (3.26, 6.74)	−0.177 (−1.229, 0.887)
Caribbean	4.11 (2.52, 6.91)	6.05 (4.66, 7.93)	1.259 (0.051, 2.482)	80.80 (51.04, 134.56)	122.30 (93.34, 160.42)	1.387 (0.504, 2.277)
Central Asia	0.01 (0.01, 0.01)	0.05 (0.03, 0.08)	6.046 (5.242, 6.857)	0.17 (0.15, 0.23)	1.28 (0.82, 2.00)	7.150 (6.090, 8.221)
Central Europe	0.02 (0.01, 0.02)	0.05 (0.04, 0.07)	3.945 (2.600, 5.307)	0.34 (0.25, 0.41)	1.59 (1.15, 2.12)	5.570 (4.704, 6.442)
Central Latin America	0.52 (0.50, 0.55)	1.76 (1.48, 2.19)	4.376 (3.547, 5.213)	10.24 (9.23, 12.30)	37.68 (30.70, 46.35)	4.751 (3.486, 6.031)
Central Sub-Saharan Africa	34.90 (22.14, 52.25)	24.39 (18.62, 31.42)	−1.288 (−1.881, −0.692)	684.17 (456.45, 1 013.16)	488.22 (374.79, 633.78)	−1.242 (−1.752, −0.730)
East Asia	0.33 (0.13, 0.48)	3.09 (2.30, 3.80)	7.813 (6.251, 9.398)	7.28 (4.07, 9.72)	58.47 (41.90, 76.99)	7.425 (6.243, 8.621)
Eastern Europe	0.02 (0.02, 0.02)	0.09 (0.09, 0.09)	6.202 (3.856, 8.602)	0.31 (0.28, 0.36)	2.47 (2.04, 2.86)	7.439 (6.314, 8.575)
Eastern Sub-Saharan Africa	77.39 (48.43, 111.91)	52.11 (42.74, 67.20)	−1.515 (−1.906, −1.123)	1 517.22 (1 007.24, 2 114.73)	1 106.33 (896.48, 1 421.81)	−1.234 (−1.635, −0.831)
High-income Asia Pacific	0.02 (0.02, 0.02)	0.07 (0.06, 0.07)	4.279 (3.004, 5.569)	0.46 (0.37, 0.56)	5.14 (2.91, 8.58)	8.576 (7.604, 9.557)
High-income North America	0.75 (0.74, 0.76)	0.54 (0.53, 0.56)	−1.380 (−2.775, 0.035)	15.23 (13.74, 17.57)	30.74 (19.56, 46.77)	2.062 (1.052, 3.082)
North Africa and Middle East	0.17 (0.05, 0.63)	1.02 (0.46, 2.94)	6.435 (5.884, 6.989)	3.60 (1.28, 11.99)	20.19 (8.85, 58.17)	6.126 (5.572, 6.682)
Oceania	0.23 (0.04, 1.11)	21.47 (8.07, 58.69)	16.871 (15.750, 18.002)	5.05 (1.41, 21.69)	423.80 (152.55, 1 199.99)	16.550 (15.383, 17.729)
South Asia	0.06 (0.01, 0.27)	0.49 (0.17, 1.70)	7.263 (6.268, 8.267)	1.99 (0.84, 5.76)	12.02 (5.79, 37.16)	6.369 (5.760, 6.980)
Southeast Asia	0.07 (0.05, 0.11)	3.58 (2.33, 6.43)	14.128 (13.233, 15.030)	2.66 (2.00, 4.02)	80.58 (53.75, 138.68)	12.360 (11.470, 13.257)
Southern Latin America	0.48 (0.47, 0.49)	1.06 (1.03, 1.08)	3.169 (1.509, 4.856)	9.70 (8.54, 11.71)	27.16 (22.83, 33.49)	3.780 (1.932, 5.661)
Southern Sub-Saharan Africa	14.67 (5.96, 32.11)	155.04 (131.23, 186.77)	8.335 (6.439, 10.265)	314.43 (148.18, 644.92)	3 311.64 (2 824.01, 3 927.53)	8.346 (6.789, 9.927)
Tropical Latin America	0.18 (0.17, 0.18)	2.45 (2.34, 2.59)	9.781 (8.309, 11.274)	3.47 (2.92, 4.73)	61.94 (52.30, 73.79)	10.875 (5.025, 17.051)
Western Europe	0.13 (0.13, 0.14)	0.14 (0.14, 0.15)	0.255 (−0.897, 1.421)	2.68 (2.34, 3.26)	7.48 (5.47, 9.79)	3.413 (2.296, 4.542)
Western Sub-Saharan Africa	12.81 (7.80, 20.13)	32.22 (27.40, 37.88)	3.024 (2.545, 3.505)	255.68 (166.95, 388.44)	634.39 (531.01, 769.16)	3.132 (2.403, 3.867)
WHO region						
African region	34.83 (21.82, 50.09)	51.39 (44.49, 61.82)	1.324 (0.565, 2.089)	688.34 (462.09, 946.77)	1 072.31 (924.22, 1 293.58)	1.507 (0.968, 2.048)
Eastern Mediterranean region	0.28 (0.05, 0.99)	1.59 (0.68, 5.09)	6.194 (5.776, 6.614)	6.02 (1.79, 19.54)	31.48 (12.88, 99.22)	5.860 (5.472, 6.250)
European region	0.08 (0.08, 0.09)	0.12 (0.12, 0.13)	1.295 (0.272, 2.330)	1.69 (1.48, 2.04)	5.42 (4.13, 6.97)	4.000 (3.130, 4.877)
Region of the Americas	0.78 (0.70, 0.90)	1.40 (1.29, 1.57)	1.936 (1.341, 2.533)	15.61 (13.70, 18.56)	41.56 (33.67, 51.47)	3.382 (2.917, 3.849)
South-East Asia region	0.04 (0.02, 0.08)	1.47 (0.99, 2.50)	13.063 (12.476, 13.652)	1.63 (1.17, 2.79)	33.55 (23.96, 54.44)	10.744 (10.215, 11.276)
Western Pacific region	0.25 (0.11, 0.35)	2.29 (1.71, 2.80)	7.321 (5.551, 9.121)	5.51 (3.21, 7.23)	44.49 (32.35, 57.73)	7.345 (6.564, 8.132)
World Bank Income region						
Low income	44.76 (28.82, 63.76)	27.15 (22.14, 35.15)	−1.855 (−2.224, −1.485)	6.27 (5.63, 7.31)	13.58 (9.34, 19.66)	2.314 (1.550, 3.083)
Lower-middle income	1.55 (0.86, 2.56)	3.93 (3.24, 4.94)	2.987 (2.443, 3.535)	877.39 (590.85, 1 206.07)	573.64 (469.34, 748.65)	−1.594 (−2.101, −1.084)
Upper-middle income	0.34 (0.20, 0.47)	4.60 (4.02, 5.18)	9.365 (9.097, 9.634)	31.93 (20.02, 50.55)	81.59 (66.60, 104.57)	3.017 (2.528, 3.509)
High income	0.31 (0.31, 0.32)	0.28 (0.25, 0.41)	−0.884 (−1.767, 0.007)	7.48 (5.28, 9.55)	94.36 (81.62, 108.49)	9.241 (8.886, 9.596)

Note: AAPC, average annual percentage change; CI, confidence interval; DALYs, disability-adjusted life years; UI, uncertainty interval.

In 1990 and 2019, although males had a higher HIV/AIDS mortality, females (AAPC = 2.322, 95% CI 1.785, 2.862) exhibited a greater increasing trend than males (AAPC = 1.832, 95% CI 1.082, 2.586) worldwide (Supplementary Figure S5 and Supplementary Table S12). The highest increasing trend of HIV/AIDS mortality for both females (AAPC = 18.742, 95% CI 17.730, 19.763) and males (AAPC = 15.730, 95% CI 14.561, 16.911) were observed in Oceania region. The number of female deaths was decreasing in the high SDI region and the high-income North America region, in contrast to male deaths (Supplementary Table S13).

The oldest age group (95+ years) had a higher increasing trend of mortality in low SDI region, Central Europe, North Africa and Middle East, and the western Sub-Saharan Africa region (Supplementary Table S14). On the other hand, the youngest age group (0–74 years) had a higher increasing trend of mortality in high-middle SDI region, Central Asia, Central Latin America, Oceania, South Asia, Southeast Asia, southern Latin America, and tropical Latin America. In the recent decade, HIV/AIDS mortality in low SDI region and high-middle SDI region became highest in males aged 70–74 years (Supplementary Figure S6). In 2019, unsafe sex was identified as the leading risk factor for HIV/AIDS mortality, resulting in 15 381.16 (95% UI 13 402.784, 18 097.156) deaths. This accounted for 90.73% (87.09%, 93.34%) of all HIV/AIDS deaths (Supplementary Table S15).

### Trends and attributable risk factors of HIV/AIDS DALYs from 1990 to 2019 at the global, regional, national, sex, and age levels

The estimated number of DALYs due to HIV/AIDS significantly increased from 78 835.14 (95% UI 56 016.37, 105 782.21) in 1990 to 363 463.28 (95% UI 313 429.57, 434 717.84) in 2019, representing a 361.04% increase (Supplementary Table S10). The overall age-standardized DALYs increased from 39.12 (95% UI 27.79, 52.49) per 100 000 population in 1990 to 78.39 (95% UI 67.60, 93.75) in 2019 (AAPC = 2.429, 95% CI 1.775, 3.087) (Table 2 and Figure 1d). Oceania had the highest increasing trend in DALYs (AAPC = 16.550, 95% CI 15.383, 17.729). The middle SDI region experienced the largest increase in DALYs from 1990 to 2019 (AAPC = 8.991, 95% CI 8.711, 9.272). There were 17, 156, and 31 countries or territories with a decreasing, increasing, or stable trend of HIV/AIDS DALY with AAPC ranging from −1.186 to 5.391 from 1990 to 2019 (Figure 2d and Supplementary Table S16).

In 1990 and 2019, males had a higher HIV/AIDS DALY at the overall level and in most regions, except central Sub-Saharan Africa (Supplementary Figure S7 and Supplementary Table S17). Although females had a higher increasing trend of DALYs than males worldwide, males had a higher increasing trend than females in high-income North America, South Asia and western Sub-Saharan Africa. The highest increasing trend of HIV/AIDS DALYs for both females (AAPC = 18.425, 95% CI 17.246, 19.616) and males (AAPC = 15.438, 95% CI 14.345, 16.541) were observed in Oceania (Supplementary Figure S7 and Supplementary Table S17). The number of DALYs among different sexes and regions all increased (Supplementary Table S18).

The 80–84 years age group exhibited a higher increasing trend in HIV/AIDS DALYs, compared to other age groups worldwide (Supplementary Table S19). However, the 95+ years age group showed a higher increasing trend in low SDI region and in most African regions. Additionally, the 70–74 years age group exhibited a higher increasing trend in high-middle SDI region and most Asian region (Supplementary Table S19). The global HIV/AIDS DALYs, which were consistently higher in males aged 70–74 years, until the recent decade, became the highest in males aged 70–74 years in low SDI region (Supplementary Figure S8). Unsafe sex was identified as the primary risk factor for HIV/AIDS DALYs, amounting to 331 140.56 (95% UI 285 150.21, 397 281.03). This accounted for 91.12% (87.65%, 93.66%) of all HIV/AIDS DALYs in 2019 (Supplementary Table S15).

## Discussion

To the best of our knowledge, this is the first study to assess the global landscape, long-term trends, and regional differences in the burden of the older HIV/AIDS epidemic at the sex and age levels using the GBD 2019 study. The findings of this study provide a comprehensive analysis of the distribution and trends in the incidence, prevalence, mortality, and DALYs among adults aged 70 years and above.

This study found that while the global incidence of HIV/AIDS among older adults decreased, the prevalence, mortality, and DALYs increased between 1990 and 2019 have increased. Increased survivorship may be attributed to ART, resulting in a growing population of older PLWH [16]. An observational cohort-modelling study has reported that both men and women who achieved viral suppression could expect to live nearly as long as those not infected with HIV [29]. The decrease in mortality and the improvement in survival rates highlight that new HIV prevention and control measures for the older population are a priority. Older adults infected with HIV are often overlooked in prevention strategies and face barriers due to factors such as increased opportunities for new sexual partnerships, comorbidities, and engaging in unprotected sex [30]. In addition, diagnosing and treating HIV in older individuals can be challenging due to the early signs and symptoms that are often attributed to age-related diseases. Neither they nor their caregivers perceive themselves to be at risk of HIV infection [31].

Although the global incidence decreased, our study also found that the highest incidence was observed in southern Sub-Saharan Africa in 2019. Southern Sub-Saharan Africa additionally had the highest prevalence, mortality, and DALYs. The WHO has reported that there were 25.6 million PLWH in the African region in 2022 [8]. The WHO African region accounts for nearly 60% of the global new HIV infections [8]. Although the large-scale roll-out of ART has successfully led to declines in HIV-related mortality in Sub-Saharan Africa [29], our findings indicate that southern Sub-Saharan Africa still requires HIV/AIDS prevention and control measures. It is noted that challenges from the older populations of other regions also existed. High-income Asia Pacific had the highest increasing trend of prevalence. Simultaneously, the highest increasing trend of incidence, mortality, and DALYs were all observed in Oceania. Older adults are at risk of acquiring HIV due to various factors, such as divorce or death of a partner, lack of HIV-related knowledge, and failure to adopt preventive behaviours [17]. Considering the cultural and medical advances of the past decades, the acceptance of marriage terminations, the promotion of sexual liberty, and the improvements in sexual performance and safer sex in older population should be promoted, especially in high-income Asia Pacific and Oceania region [30]. In addition, the causes of uncontrolled mortality in Oceania should also be investigated. In our study, we found that unsafe sex was the primary risk factor for HIV/AIDS mortality. However, it is important not to neglect other risk factors such as drug use and IPV. Drug use significantly impacts morbidity, increases the risk of mortality, and reduces life expectancy among PLWH [32]. IPV impacts engagement in care for HIV-infected patients and predicts worse biological outcomes, including a lower level of CD4 and a higher level of viral load [33]. Therefore, in addition to traditional health education, which includes promoting safe sex and reducing drug use, implementing measures to reduce violence and discrimination can contribute to improving the survival status of PLWH.

Males had a higher disease burden than females worldwide, and regional differences should be taken into consideration. The highest increasing trends in female HIV/AIDS incidence and prevalence were observed in the Central Asia region. For males, these trends were observed in the Oceania region and the high-income Asia Pacific region, respectively. The number of HIV infections among females in Central Asia is on the rise. This increase may be attributed to the high percentage of at-risk populations, such as female sex workers (FSWs) or drug users. These population face potential challenges in accessing HIV self-testing, as well as stigma and barriers to HIV testing [34, 35]. A meta-analysis has reported that HIV prevalence in Kazakhstan ranged from 0.06% to 30.1% [36]. The highest prevalence (30.1%) was reported among women who injected drugs [36]. Alaei et al. have found that of 2 174 FSWs in Tajikistan, 2.6% tested positive for HIV [37]. Although most countries have proposed plans to curb the HIV epidemic, there are still critical problems that act as barriers, including lack of financial support, policy violence, and injectable drug use [36]. Alaei et al. have suggested that the government should consider implementing targeted testing and linkage-to-care efforts for FSWs who inject drugs as well as discover ways to reduce stigma, violence, and discrimination against FSWs [37]. In Oceania and the high-income Asia Pacific region, the transmission of HIV among male sex workers (MSWs) and male migrants was a critical issue [38, 39]. Marukutira et al. have found that 41% of eligible retained PLWH were migrants in Australia from 2013 to 2017 [38]. Turek et al. have reported that the incidence of HIV among MSWs in Melbourne, Australia, was 1.7% [39]. Most (85.6%) MSWs also had non-commercial sex partners [39]. Streamlined testing strategies, especially among migrant populations, were needed to ensure timely HIV testing and linkage to care [38]. In addition, in the recent decade, females aged 70–74 years had a higher incidence and prevalence compared to other groups, while males aged 70–74 years had a higher mortality and DALY in low SDI regions. The characteristics of HIV/AIDS at the sex and age level may be complicated by differences in sexual activities and regional gender culture. Our findings suggest that specific management measures should consider sex and age in these regions.

Older PLWH were largely neglected in the past decades. However, with the widespread use of ART, their life expectancy has increased and their case fatality rate has decreased. Besides, the rapidly aging society over time, factors such as HIV acquisition risk, and others may contribute to changes in the HIV/AIDS epidemic among older adults. However, data collection regarding HIV/AIDS in older adults is often omitted. We used GBD data to assess the global landscape, long-term trends, and regional differences in the burden of HIV/AIDS diseases among older individuals, considering sex and age. There were still several limitations in this study. Firstly, the most notable limitation of this analysis was that the accuracy and robustness of GBD estimates largely depend on the quality and quantity of data used in the modelling [25]. For countries lacking or having insufficient national systematic surveillance and population-based studies of HIV/AIDS, the estimates might be subject to a margin of bias. Secondly, due to GBD only providing data for the population aged 70 years and above, and not 50 years and above, we were unable to analyse this population.

## Conclusions

The global prevalence, mortality, and DALYs among older adults increased between 1990 and 2019, while the global incidence decreased. The disease burden of HIV varied between regions, age groups, and sexes. It is therefore imperative to provide detailed sexual health education and targeted screening for older adults.

## Supporting information

Du et al. supplementary material Du et al. supplementary material

## Data Availability

The datasets analysed during the current study are available in the GBD repository (http://ghdx.healthdata.org/gbd-results-tool).

## Supplementary material

For supplementary material accompanying this paper visit http://doi.org/10.1017/S0950268823001954. click here to view supplementary material
