# Supplementary material for: Global, regional, and national disease burden and attributable risk factors of HIV/AIDS in older adults aged 70 years and above: a trend analysis based on the Global Burden of Disease study 2019
Source: Epidemiol Infect. 2023 Dec 15;152:e2. doi: 10.1017/S0950268823001954 (PMC10789987; doi:10.1017/S0950268823001954)
Supplement: Du et al. supplementary material [file S0950268823001954sup001.docx]

**Epidemiology and Infection**

**Title:** Global, regional, and national disease burden and attributable risk factors of HIV/AIDs in older adults aged 70 years and older: a trend analysis based on the Global Burden of Disease Study 2019

**Authors:** Min Du, Min Liu, Jue Liu

**Supplementary Material**

[Supplementary Table S1. The number of HIV/AIDs incident cases and HIV/AIDs infections among older adults in 204 countries or territories from 1990 to 2019. 1](#_Toc150540035)

[Supplementary Table S2. The number of incident cases and incidence of HIV/AIDs among older adults in 204 countries or territories from 1990 to 2019. 3](#_Toc150540036)

[Supplementary Table S3. The HIV/AIDs incidence and its trends among older adults by region and sex level. 13](#_Toc150540037)

[Supplementary Table S4. The incident cases of HIV/AIDs and its changes among older adults by region and sex level. 15](#_Toc150540038)

[Supplementary Figure S1. The incidence, prevalence, mortality, and rate of DALYs in HIV/AIDs by age group at overall, SDI, GBD, WHO and World Bank income regional level in 2019. 17](#_Toc150540039)

[Supplementary Table S5. Average annual percentage changes of incidence rate by region and age from 2010 to 2019. 18](#_Toc150540040)

[Supplementary Figure S2. Sex and age group distribution of HIV/AIDs incidence by SDI region from1990 to 2019. 19](#_Toc150540041)

[Supplementary Table S6. The number of cases and prevalence of HIV/AIDs among older adults in 204 countries or territories from 1990 to 2019. 20](#_Toc150540042)

[Supplementary Figure S3. The prevalence and its trend of HIV/AIDs from 1990 to 2019 by global, SDI, GBD, WHO and World Bank income regions. 26](#_Toc150540044)

[Supplementary Table S7. The HIV/AIDs prevalence and its trends among older adults by region and sex level from 1990 to 2019. 27](#_Toc150540045)

[Supplementary Table S8. The HIV/AIDs cases and its changes among older adults by region and sex level in 1990 and 2019. 30](#_Toc150540046)

[Supplementary Table S9. Average annual percentage changes of prevalence by region and age from 2010 to 2019. 33](#_Toc150540047)

[Supplementary Figure S4. Sex and age group distribution of HIV/AIDs prevalence by SDI region from1990 to 2019. 37](#_Toc150540048)

[Supplementary Table S10. The number of HIV/AIDs deaths and DALYs among older adults from 1990 to 2019. 38](#_Toc150540049)

[Supplementary Table S11. The number of HIV/AIDs deaths and mortality among older adults in 204 countries or territories from 1990 to 2019. 40](#_Toc150540050)

[Supplementary Figure S5. The mortality and its trend of HIV/AIDs from 1990 to 2019 in global, SDI, GBD, WHO and World Bank income regions by sex. 46](#_Toc150540052)

[Supplementary Table S12. The HIV/AIDs mortality and its trends among older adults by region and sex level from 1990 to 2019. 47](#_Toc150540053)

[Supplementary Table S13. The HIV/AIDs deaths and its changes among older adults by region and sex level in 1990 and 2019. 50](#_Toc150540054)

[Supplementary Table S14. Average annual percentage changes of mortality by region and age from 2010 to 2019. 52](#_Toc150540055)

[Supplementary Figure S6. Sex and age group distribution of HIV/AIDs mortality by SDI region from1990 to 2019. 54](#_Toc150540056)

[Supplementary Table S15. The attributable risk factor of HIV/AIDs mortality and DALYs in 2019 55](#_Toc150540057)

[Supplementary Table S16. The number of HIV/AIDs DALYs and DALY rate among older adults in 204 countries or territories from 1990 to 2019. 56](#_Toc150540058)

[Supplementary Figure S7. The DALY rate and its trend of HIV/AIDs from 1990 to 2019 in global, SDI, GBD, WHO and World Bank income regions by sex. 66](#_Toc150540059)

[Supplementary Table S17. The HIV/AIDs DALY rate and its trends among older adults by region and sex level from 1990 to 2019. 67](#_Toc150540060)

[Supplementary Table S18. The HIV/AIDs DALYs and its changes among older adults by region and sex level in 1990 and 2019. 70](#_Toc150540061)

[Supplementary Table S19. Average annual percentage changes of DALY rate by region and age from 2010 to 2019. 73](#_Toc150540062)

[Supplementary Figure S8. Sex and age group distribution of HIV/AIDs DALY rate by SDI region from1990 to 2019. 75](#_Toc150540064)

**Supplementary Table S1**. The number of HIV/AIDs incident cases and HIV/AIDs infections among older adults in 204 countries or territories from 1990 to 2019.

|  | Incident cases |  |  | Cases |  |  |
| --- | --- | --- | --- | --- | --- | --- |
|  | 1990 | 2019 | Percentage change (%) | 1990 (95% UI) | 2019 (95% UI) | Percentage change (%) |
| **Global** | 15725.46 (12696.54, 19389.42) | 15001.57 (12337.48, 18415.62) | -4.60 | 70296.17 (60463.91, 83793.43) | 781833.70 (676993.34, 895975.55) | 1012.20 |
| **SDI region** | |  |  |  |  |  |
| Low SDI | 7832.60 (5607.53, 10394.24) | 3358.55 (2405.91, 4615.07) | -57.12 | 38387.45 (30978.97, 46828.17) | 139033.28 (117500.42, 166271.75) | 262.18 |
| Low-middle SDI | 4961.87 (3646.43, 6826.38) | 3272.48 (2508.19, 4266.47) | -34.05 | 15044.65 (11722.45, 20695.77) | 146133.12 (127767.44, 171334.57) | 871.33 |
| Middle SDI | 1884.83 (1395.70, 3093.07) | 7035.86 (5631.40, 8790.51) | 273.29 | 7211.66 (5146.46, 12099.90) | 288911.97 (253780.34, 326282.93) | 3906.18 |
| High-middle SDI | 449.97 (295.96, 773.24) | 623.54 (444.62, 823.87) | 38.57 | 2615.08 (1896.86, 3789.74) | 37285.46 (33377.43, 41737.41) | 1325.79 |
| High SDI | 576.16 (407.15, 830.83) | 700.27 (316.18, 1160.88) | 21.54 | 6964.21 (4596.03, 10544.99) | 169982.63 (96391.40, 246917.01) | 2340.80 |
| **GBD region** |  |  |  |  |  |  |
| Andean Latin America | 25.96 (12.74, 65.04) | 88.86 (31.15, 171.54) | 242.30 | 253.66 (136.86, 597.62) | 3582.08 (1900.71, 5393.20) | 1312.17 |
| Australasia | 7.73 (4.61, 10.86) | 10.08 (5.26, 16.66) | 30.34 | 80.51 (59.05, 110.02) | 1737.26 (1303.86, 2097.43) | 2057.79 |
| Caribbean | 390.31 (265.23, 595.28) | 150.28 (84.66, 248.52) | -61.50 | 1269.14 (802.61, 1985.77) | 6794.52 (5484.49, 8356.79) | 435.36 |
| Central Asia | 1.49 (0.59, 2.64) | 2.55 (0.85, 5.36) | 71.22 | 9.31 (4.96, 23.34) | 152.22 (100.94, 207.13) | 1534.40 |
| Central Europe | 6.99 (4.89, 10.55) | 5.16 (2.15, 9.95) | -26.21 | 34.44 (23.24, 52.02) | 1447.87 (908.80, 2054.42) | 4104.40 |
| Central Latin America | 124.48 (88.06, 206.63) | 239.89 (114.10, 380.53) | 92.72 | 710.14 (392.49, 1451.98) | 13653.20 (8905.04, 17740.00) | 1822.60 |
| Central Sub-Saharan Africa | 933.04 (629.33, 1310.03) | 448.81 (287.54, 659.79) | -51.90 | 3851.06 (2643.00, 5524.06) | 11137.94 (8507.96, 14604.51) | 189.22 |
| East Asia | 673.04 (315.54, 1753.65) | 1558.22 (742.20, 2305.15) | 131.52 | 4320.89 (2741.70, 10052.12) | 61560.45 (29497.55, 110974.54) | 1324.72 |
| Eastern Europe | 7.16 (4.83, 10.49) | 27.55 (3.74, 62.77) | 285.06 | 39.00 (26.01, 63.17) | 2009.70 (1271.11, 2894.76) | 5052.84 |
| Eastern Sub-Saharan Africa | 7956.09 (5848.98, 10280.77) | 3235.56 (2344.99, 4440.24) | -59.33 | 35642.76 (28934.52, 43105.84) | 147496.17 (125092.75, 176938.70) | 313.82 |
| High-income Asia Pacific | 29.66 (14.59, 52.41) | 90.80 (33.54, 196.31) | 206.18 | 99.12 (47.42, 172.92) | 14471.25 (7666.53, 21374.00) | 14499.90 |
| High-income North America | 352.08 (189.90, 500.13) | 440.14 (75.11, 848.57) | 25.01 | 5289.58 (3370.45, 8120.74) | 118400.22 (61306.89, 182197.68) | 2138.37 |
| North Africa and Middle East | 51.59 (10.89, 156.72) | 186.64 (58.29, 626.58) | 261.77 | 273.94 (126.89, 746.62) | 3141.39 (1506.76, 7491.01) | 1046.76 |
| Oceania | 2.40 (0.47, 10.62) | 50.69 (1.19, 181.64) | 2008.34 | 12.33 (3.09, 48.89) | 969.55 (50.61, 2603.53) | 7764.51 |
| South Asia | 205.83 (69.04, 656.71) | 328.56 (85.24, 768.74) | 59.63 | 1070.64 (547.62, 3232.53) | 17731.16 (12207.49, 26022.24) | 1556.13 |
| Southeast Asia | 548.51 (210.41, 1482.24) | 311.55 (155.07, 575.69) | -43.20 | 1280.71 (611.50, 3103.10) | 37320.17 (24487.47, 54660.46) | 2814.03 |
| Southern Latin America | 96.51 (47.02, 245.83) | 65.16 (44.06, 90.49) | -32.49 | 668.80 (443.24, 1009.92) | 5104.98 (4382.26, 5838.41) | 663.30 |
| Southern Sub-Saharan Africa | 1934.69 (883.02, 3386.39) | 5127.57 (3971.00, 6655.58) | 165.03 | 4798.81 (2576.43, 9115.27) | 207128.94 (182432.68, 236360.93) | 4216.26 |
| Tropical Latin America | 47.04 (37.11, 60.60) | 653.14 (314.87, 1057.51) | 1288.63 | 289.70 (192.65, 478.35) | 28550.05 (17199.62, 41395.92) | 9755.21 |
| Western Europe | 246.31 (172.45, 403.14) | 197.25 (134.21, 327.22) | -19.92 | 2031.94 (1328.97, 3179.18) | 44007.00 (32289.76, 53602.67) | 2065.76 |
| Western Sub-Saharan Africa | 2084.56 (1411.95, 2995.69) | 1783.11 (1428.31, 2253.60) | -14.46 | 8269.69 (6154.30, 11733.60) | 55437.59 (46099.48, 68279.82) | 570.37 |
| **WHO region** | |  |  |  |  |  |
| African Region | 12898.37 (9505.23, 16769.65) | 10578.07 (8480.41, 13176.49) | -17.99 | 52532.24 (43314.73, 62562.22) | 421085.93 (372299.74, 484333.06) | 701.58 |
| Eastern Mediterranean Region | 87.36 (16.26, 264.74) | 235.67 (78.99, 704.52) | 169.76 | 663.88 (253.22, 1949.36) | 3741.58 (1669.24, 9296.53) | 463.59 |
| European Region | 263.33 (187.55, 423.42) | 235.48 (162.58, 371.67) | -10.58 | 2122.30 (1424.16, 3253.82) | 47757.38 (35973.74, 57712.06) | 2150.26 |
| Region of the Americas | 985.74 (773.57, 1247.55) | 1623.81 (893.52, 2310.15) | 64.73 | 8263.42 (5944.88, 11633.79) | 175524.52 (115168.00, 239012.59) | 2024.11 |
| South-East Asia Region | 675.18 (255.08, 1803.98) | 532.39 (230.38, 1038.87) | -21.15 | 1723.90 (899.97, 4212.34) | 50045.91 (33582.58, 69923.33) | 2803.06 |
| Western Pacific Region | 756.56 (390.30, 1840.83) | 1775.19 (927.38, 2476.15) | 134.64 | 4731.89 (3036.26, 10439.90) | 82791.82 (50975.95, 130128.25) | 1649.65 |
| **World Bank income region** |  |  |  |  |  |  |
| Low income | 8227.81 (5584.57, 11243.04) | 3311.15 (2365.65, 4589.24) | -59.76 | 39029.68 (30920.67, 47909.16) | 147101.85 (125337.21, 176408.02) | 276.90 |
| Lower-middle income | 4833.96 (3946.83, 6055.94) | 3513.28 (2699.25, 4595.90) | -27.32 | 14601.38 (11914.99, 18927.10) | 136277.66 (119067.85, 158017.52) | 833.32 |
| Upper-middle income | 1872.22 (1389.54, 3127.12) | 7328.70 (5802.02, 9108.03) | 291.45 | 8355.05 (5838.13, 14509.28) | 315361.84 (276948.99, 355525.62) | 3674.51 |
| High income | 771.45 (556.54, 1090.39) | 837.50 (443.87, 1316.25) | 8.56 | 8236.93 (5407.37, 12484.50) | 182604.46 (108035.64, 260497.98) | 2116.90 |

Note: UI, uncertainty interval

**Supplementary Table S2**. The number of incident cases and incidence of HIV/AIDs among older adults in 204 countries or territories from 1990 to 2019.

|  | Number | | | ASR (per 100,000 population) | | |
| --- | --- | --- | --- | --- | --- | --- |
| Countries or territories | 1990 (95% UI) | 2019 (95% UI) | Percentage change (%) | 1990 (95% UI) | 2019 (95% UI) | AAPC (95% CI) |
| Afghanistan | 2.84 (0.00, 20.00) | 1.60 (0.00, 13.13) | -43.7391698 | 0.87 (0.00, 6.10) | 0.32 (0.00, 2.64) | -4.614 (-6.426, -2.767) |
| Albania | 0.09 (0.04, 0.16) | 0.00 (0.00, 0.00) | -97.6006143 | 0.09 (0.04, 0.16) | 0.00 (0.00, 0.00) | -15.214 (-17.809, -12.538) |
| Algeria | 0.24 (0.00, 1.39) | 3.42 (0.00, 26.07) | 1316.6881 | 0.04 (0.00, 0.23) | 0.21 (0.00, 1.63) | 5.813 (4.188, 7.462) |
| American Samoa | 0.01 (0.00, 0.07) | 0.01 (0.00, 0.02) | -42.2711625 | 1.32 (0.12, 8.06) | 0.31 (0.07, 0.98) | -5.087 (-6.677, -3.470) |
| Andorra | 0.08 (0.00, 0.42) | 0.11 (0.00, 0.66) | 35.1657907 | 2.66 (0.01, 14.26) | 1.25 (0.01, 7.82) | -0.205 (-2.891, 2.556) |
| Angola | 14.73 (4.21, 32.96) | 237.18 (120.30, 406.25) | 1510.69621 | 11.16 (3.19, 24.98) | 62.73 (31.81, 107.44) | 6.108 (5.623, 6.595) |
| Antigua and Barbuda | 0.65 (0.48, 0.93) | 0.33 (0.07, 0.70) | -49.5276417 | 17.84 (13.35, 25.58) | 6.19 (1.26, 13.29) | -3.940 (-5.628, -2.223) |
| Argentina | 21.83 (17.01, 28.62) | 27.30 (13.87, 49.83) | 25.061856 | 1.18 (0.92, 1.55) | 0.80 (0.41, 1.46) | -1.397 (-1.817, -0.975) |
| Armenia | 0.02 (0.00, 0.18) | 0.01 (0.00, 0.03) | -50.7982647 | 0.02 (0.00, 0.16) | 0.00 (0.00, 0.01) | -4.723 (-7.285, -2.090) |
| Australia | 7.35 (4.15, 10.55) | 7.77 (3.52, 13.68) | 5.84361046 | 0.61 (0.34, 0.87) | 0.28 (0.13, 0.49) | -2.722 (-4.159, -1.264) |
| Austria | 17.74 (0.00, 49.21) | 2.71 (0.70, 6.03) | -84.7429302 | 2.32 (0.00, 6.44) | 0.22 (0.06, 0.49) | -9.217 (-11.966, -6.383) |
| Azerbaijan | 0.01 (0.01, 0.06) | 0.01 (0.00, 0.01) | -63.1143174 | 0.01 (0.00, 0.03) | 0.00 (0.00, 0.00) | -3.833 (-6.327, -1.274) |
| Bahamas | 3.76 (2.80, 5.45) | 0.95 (0.49, 1.53) | -74.7504431 | 46.73 (34.78, 67.74) | 5.07 (2.63, 8.15) | -9.318 (-11.228, -7.366) |
| Bahrain | 0.04 (0.02, 0.06) | 0.02 (0.01, 0.04) | -43.3773083 | 0.60 (0.36, 1.01) | 0.09 (0.04, 0.15) | -6.413 (-7.273, -5.545) |
| Bangladesh | 0.00 (0.00, 0.00) | 3.95 (0.00, 24.46) | - | 0.03 (0.17, 0.00) | 0.06 (0.00, 0.35) | 2.608 (-0.544, 5.861) |
| Barbados | 5.18 (3.78, 7.95) | 1.15 (0.42, 2.41) | -77.8509469 | 24.22 (17.70, 37.19) | 3.77 (1.38, 7.93) | -6.813 (-8.865, -4.714) |
| Belarus | 0.09 (0.00, 0.79) | 0.00 (0.00, 0.00) | -98.768968 | 0.01 (0.00, 0.11) | 0.00 (0.00, 0.00) | -15.593 (-20.200, -10.720) |
| Belgium | 21.20 (11.12, 42.54) | 17.89 (4.07, 37.84) | -15.6107649 | 2.20 (1.16, 4.42) | 1.15 (0.26, 2.43) | -1.975 (-3.014, -0.925) |
| Belize | 1.79 (1.23, 2.77) | 0.73 (0.12, 1.70) | -59.0768966 | 34.28 (23.62, 53.00) | 5.68 (0.94, 13.21) | -6.224 (-8.076, -4.334) |
| Benin | 16.56 (8.37, 28.80) | 20.33 (9.20, 39.76) | 22.7680485 | 16.08 (8.13, 27.97) | 9.33 (4.23, 18.26) | -1.904 (-3.151, -0.642) |
| Bermuda | 1.08 (0.81, 1.62) | 0.39 (0.12, 0.74) | -64.0052124 | 31.00 (23.26, 46.35) | 4.60 (1.44, 8.75) | -6.336 (-8.178, -4.458) |
| Bhutan | 0.39 (0.00, 2.28) | 0.40 (0.00, 2.65) | 0.82629622 | 4.28 (0.01, 24.86) | 1.38 (0.00, 9.23) | -4.686 (-6.381, -2.961) |
| Bolivia (Plurinational State of) | 9.82 (0.06, 48.72) | 9.82 (0.11, 47.76) | -0.01889409 | 6.50 (0.04, 32.25) | 2.16 (0.02, 10.51) | -3.771 (-4.307, -3.231) |
| Bosnia and Herzegovina | 0.00 (0.00, 0.01) | 0.01 (0.00, 0.03) | 152.201741 | 0.00 (0.00, 0.00) | 0.00 (0.00, 0.01) | 0.175 (-1.597, 1.979) |
| Botswana | 142.23 (90.31, 199.41) | 71.18 (29.39, 139.80) | -49.9564561 | 573.88 (364.39, 804.60) | 133.85 (55.28, 262.90) | -4.934 (-5.118, -4.748) |
| Brazil | 46.43 (36.59, 59.82) | 647.42 (312.85, 1047.58) | 1294.50924 | 1.10 (0.86, 1.41) | 4.95 (2.39, 8.00) | 5.423 (4.865, 5.985) |
| Brunei Darussalam | 0.04 (0.02, 0.06) | 0.09 (0.03, 0.18) | 148.297764 | 1.00 (0.54, 1.60) | 0.93 (0.34, 1.79) | -0.402 (-1.867, 1.084) |
| Bulgaria | 0.06 (0.00, 0.15) | 0.00 (0.00, 0.01) | -94.8595597 | 0.01 (0.00, 0.02) | 0.00 (0.00, 0.00) | -11.210 (-13.265, -9.106) |
| Burkina Faso | 277.26 (119.03, 515.33) | 15.63 (6.86, 31.32) | -94.3633563 | 140.98 (60.52, 262.03) | 4.00 (1.75, 8.01) | -11.571 (-11.889, -11.252) |
| Burundi | 334.69 (83.22, 651.17) | 7.38 (3.09, 14.63) | -97.7944278 | 291.62 (72.51, 567.37) | 4.51 (1.89, 8.94) | -13.552 (-13.970, -13.132) |
| Cabo Verde | 3.48 (1.25, 7.18) | 0.97 (0.21, 3.17) | -72.0557235 | 22.95 (8.20, 47.29) | 4.65 (1.02, 15.12) | -5.413 (-5.530, -5.296) |
| Cambodia | 10.27 (3.12, 20.16) | 8.60 (0.72, 23.16) | -16.3388066 | 5.66 (1.72, 11.10) | 1.61 (0.13, 4.34) | -4.847 (-7.494, -2.124) |
| Cameroon | 113.84 (60.68, 183.58) | 191.71 (102.23, 313.65) | 68.4047714 | 61.72 (32.90, 99.54) | 39.19 (20.90, 64.12) | -1.611 (-1.969, -1.252) |
| Canada | 13.63 (5.67, 21.89) | 2.74 (0.37, 6.12) | -79.8711086 | 0.68 (0.28, 1.09) | 0.06 (0.01, 0.14) | -8.298 (-9.686, -6.889) |
| Central African Republic | 242.11 (127.36, 391.65) | 38.67 (13.43, 90.93) | -84.0277877 | 609.99 (320.87, 986.74) | 57.42 (19.94, 135.01) | -7.943 (-8.256, -7.628) |
| Chad | 73.52 (24.45, 162.81) | 60.26 (29.34, 114.22) | -18.0418738 | 48.75 (16.21, 107.95) | 24.75 (12.05, 46.91) | -2.289 (-3.120, -1.451) |
| Chile | 3.55 (2.53, 4.51) | 25.00 (13.26, 39.12) | 604.744569 | 0.66 (0.47, 0.85) | 1.73 (0.92, 2.71) | 3.325 (2.578, 4.077) |
| China | 659.92 (311.02, 1746.74) | 1525.13 (732.15, 2272.50) | 131.109294 | 1.72 (0.81, 4.57) | 1.41 (0.68, 2.10) | -1.359 (-3.471, 0.799) |
| Colombia | 17.18 (11.20, 29.01) | 75.20 (35.73, 132.02) | 337.785896 | 2.07 (1.35, 3.49) | 2.43 (1.16, 4.27) | 1.086 (-2.281, 4.569) |
| Comoros | 0.05 (0.00, 0.24) | 0.23 (0.00, 1.52) | 331.584996 | 0.45 (0.00, 2.08) | 0.89 (0.00, 5.98) | 2.362 (1.279, 3.456) |
| Congo | 102.98 (49.04, 183.76) | 42.32 (13.28, 108.04) | -58.9071002 | 232.95 (110.93, 415.70) | 42.48 (13.33, 108.45) | -5.722 (-5.878, -5.566) |
| Cook Islands | 0.00 (0.00, 0.02) | 0.06 (0.00, 0.29) | 2283.5705 | 0.43 (0.02, 2.57) | 4.46 (0.10, 21.02) | 8.335 (7.140, 9.544) |
| Costa Rica | 1.28 (1.02, 1.53) | 1.15 (0.47, 2.23) | -10.5400299 | 1.35 (1.07, 1.62) | 0.40 (0.16, 0.78) | -4.267 (-5.289, -3.234) |
| Croatia | 0.42 (0.26, 0.59) | 0.27 (0.05, 0.62) | -36.9640704 | 0.13 (0.08, 0.18) | 0.05 (0.01, 0.10) | -3.210 (-6.068, -0.265) |
| Cuba | 1.48 (1.13, 2.06) | 15.72 (5.25, 39.16) | 962.580982 | 0.23 (0.18, 0.33) | 1.30 (0.43, 3.23) | 5.329 (3.172, 7.531) |
| Cyprus | 0.02 (0.01, 0.03) | 0.31 (0.13, 0.55) | 1834.03468 | 0.03 (0.01, 0.05) | 0.25 (0.10, 0.44) | 7.697 (5.864, 9.562) |
| Czechia | 0.63 (0.34, 1.10) | 1.14 (0.25, 2.38) | 80.1752104 | 0.08 (0.04, 0.14) | 0.08 (0.02, 0.16) | -0.524 (-3.070, 2.089) |
| Cote d'Ivoire | 487.71 (135.77, 989.25) | 80.39 (42.84, 138.98) | -83.5172762 | 365.07 (101.63, 740.50) | 20.25 (10.79, 35.01) | -9.695 (-9.945, -9.444) |
| Democratic People's Republic of Korea | 7.04 (0.01, 49.27) | 12.75 (0.06, 84.92) | 81.0169492 | 1.11 (0.00, 7.75) | 0.70 (0.00, 4.68) | -10.609 (-21.803, 2.189) |
| Democratic Republic of the Congo | 552.74 (328.97, 827.01) | 67.61 (28.90, 127.98) | -87.7680508 | 94.29 (56.11, 141.07) | 5.03 (2.15, 9.52) | -9.678 (-9.884, -9.472) |
| Denmark | 8.13 (4.88, 12.33) | 4.23 (1.50, 8.86) | -47.9152925 | 1.45 (0.87, 2.20) | 0.52 (0.18, 1.08) | -3.533 (-4.366, -2.693) |
| Djibouti | 0.22 (0.03, 0.76) | 5.73 (2.05, 13.32) | 2476.46617 | 5.14 (0.68, 17.61) | 27.43 (9.82, 63.78) | 5.405 (4.717, 6.098) |
| Dominica | 0.72 (0.53, 1.10) | 0.33 (0.05, 0.77) | -54.2070393 | 14.26 (10.40, 21.64) | 6.08 (0.92, 14.13) | -3.155 (-4.239, -2.060) |
| Dominican Republic | 76.16 (38.31, 131.91) | 39.07 (12.01, 93.22) | -48.6977046 | 40.95 (20.60, 70.93) | 7.74 (2.38, 18.47) | -5.485 (-5.922, -5.047) |
| Ecuador | 3.13 (1.89, 6.85) | 18.99 (7.10, 37.45) | 507.090483 | 1.15 (0.70, 2.52) | 2.26 (0.84, 4.45) | 2.466 (1.374, 3.571) |
| Egypt | 1.48 (0.86, 2.55) | 3.32 (0.99, 6.29) | 125.091136 | 0.12 (0.07, 0.20) | 0.13 (0.04, 0.25) | 0.740 (-0.745, 2.247) |
| El Salvador | 8.26 (6.00, 12.26) | 29.76 (5.22, 70.40) | 260.276585 | 5.24 (3.80, 7.77) | 8.09 (1.42, 19.14) | 1.685 (-0.319, 3.730) |
| Equatorial Guinea | 2.17 (0.71, 5.29) | 43.96 (14.85, 99.02) | 1926.80675 | 27.32 (8.93, 66.66) | 225.71 (76.24, 508.38) | 7.561 (6.977, 8.149) |
| Eritrea | 23.12 (6.19, 49.66) | 4.96 (2.18, 9.84) | -78.5326394 | 79.15 (21.19, 170.00) | 5.65 (2.48, 11.20) | -8.847 (-9.172, -8.520) |
| Estonia | 0.00 (0.00, 0.01) | 0.00 (0.00, 0.00) | -87.9784425 | 0.00 (0.00, 0.01) | 0.00 (0.00, 0.00) | -9.518 (-11.604, -7.383) |
| Eswatini | 6.26 (0.21, 22.07) | 57.06 (27.75, 105.47) | 812.200677 | 49.99 (1.71, 176.37) | 225.16 (109.50, 416.16) | 4.836 (3.256, 6.440) |
| Ethiopia | 1083.39 (553.24, 1809.48) | 137.60 (95.26, 194.70) | -87.2993679 | 140.89 (71.94, 235.31) | 7.28 (5.04, 10.31) | -9.834 (-10.372, -9.292) |
| Fiji | 0.30 (0.10, 1.22) | 0.59 (0.18, 1.28) | 100.038591 | 2.18 (0.72, 8.95) | 1.94 (0.60, 4.19) | 0.897 (-1.664, 3.525) |
| Finland | 0.37 (0.23, 0.54) | 0.63 (0.16, 2.14) | 68.839606 | 0.08 (0.05, 0.12) | 0.07 (0.02, 0.24) | -0.094 (-1.543, 1.375) |
| France | 13.57 (9.58, 18.12) | 17.16 (8.20, 36.88) | 26.4522034 | 0.26 (0.18, 0.35) | 0.18 (0.09, 0.39) | -1.565 (-2.492, -0.628) |
| Gabon | 18.31 (4.26, 49.33) | 19.07 (6.30, 44.15) | 4.16068502 | 68.47 (15.93, 184.48) | 44.42 (14.66, 102.82) | -1.551 (-1.980, -1.120) |
| Gambia | 2.31 (0.87, 4.49) | 16.61 (7.04, 30.90) | 618.545422 | 14.78 (5.53, 28.72) | 35.65 (15.10, 66.31) | 2.933 (2.423, 3.445) |
| Georgia | 0.03 (0.00, 0.14) | 0.49 (0.07, 1.10) | 1373.32819 | 0.01 (0.00, 0.05) | 0.13 (0.02, 0.30) | 11.396 (8.378, 14.499) |
| Germany | 25.35 (17.11, 33.64) | 20.59 (9.51, 40.67) | -18.7728878 | 0.31 (0.21, 0.42) | 0.16 (0.07, 0.31) | -2.411 (-3.389, -1.424) |
| Ghana | 145.90 (99.06, 205.58) | 135.13 (78.32, 215.01) | -7.37898531 | 57.31 (38.91, 80.75) | 19.82 (11.49, 31.54) | -3.618 (-3.716, -3.520) |
| Greece | 0.61 (0.32, 0.95) | 0.17 (0.04, 0.80) | -72.5326239 | 0.06 (0.03, 0.10) | 0.01 (0.00, 0.05) | -6.625 (-7.940, -5.291) |
| Greenland | 0.06 (0.03, 0.10) | 0.03 (0.00, 0.08) | -50.9441181 | 4.77 (2.27, 7.86) | 0.96 (0.13, 2.54) | -4.754 (-6.297, -3.186) |
| Grenada | 0.73 (0.50, 1.08) | 0.22 (0.05, 0.46) | -69.0856204 | 14.41 (9.93, 21.47) | 3.80 (0.81, 7.74) | -4.543 (-5.626, -3.448) |
| Guam | 0.16 (0.01, 1.01) | 0.06 (0.02, 0.19) | -60.7440641 | 5.75 (0.53, 36.29) | 0.61 (0.16, 1.85) | -7.743 (-9.714, -5.730) |
| Guatemala | 8.08 (2.88, 25.51) | 5.98 (2.02, 13.74) | -25.9807956 | 5.28 (1.88, 16.66) | 0.98 (0.33, 2.25) | -5.483 (-6.852, -4.095) |
| Guinea | 29.67 (15.10, 51.40) | 88.91 (39.76, 175.15) | 199.708483 | 17.95 (9.14, 31.10) | 34.22 (15.30, 67.40) | 2.255 (1.838, 2.674) |
| Guinea-Bissau | 5.53 (2.36, 10.29) | 8.79 (2.68, 18.62) | 59.0498321 | 31.71 (13.52, 59.00) | 31.41 (9.58, 66.51) | -0.043 (-0.426, 0.342) |
| Guyana | 6.80 (4.84, 10.48) | 4.12 (0.37, 14.56) | -39.4888312 | 38.85 (27.62, 59.84) | 15.08 (1.35, 53.35) | -3.405 (-6.883, 0.203) |
| Haiti | 226.85 (109.41, 418.07) | 66.41 (28.30, 130.44) | -70.7275569 | 165.64 (79.89, 305.27) | 22.73 (9.69, 44.65) | -6.690 (-6.965, -6.413) |
| Honduras | 0.00 (0.00, 0.00) | 0.01 (0.00, 0.03) | 269.302436 | 0.00 (0.00, 0.00) | 0.00 (0.00, 0.01) | 0.305 (-0.848, 1.472) |
| Hungary | 0.02 (0.01, 0.03) | 0.00 (0.00, 0.00) | -93.6069811 | 0.00 (0.00, 0.00) | 0.00 (0.00, 0.00) | -10.598 (-12.420, -8.738) |
| Iceland | 0.25 (0.10, 0.47) | 0.00 (0.00, 0.02) | -98.608812 | 1.36 (0.55, 2.59) | 0.01 (0.00, 0.07) | -16.570 (-20.847, -12.061) |
| India | 171.89 (64.74, 461.69) | 278.59 (76.57, 627.52) | 62.0778901 | 1.05 (0.40, 2.82) | 0.49 (0.14, 1.11) | -2.477 (-2.987, -1.965) |
| Indonesia | 214.58 (0.00, 865.61) | 80.27 (19.52, 207.63) | -62.5911958 | 5.63 (0.00, 22.71) | 0.89 (0.22, 2.31) | -6.895 (-11.276, -2.297) |
| Iran (Islamic Republic of) | 3.98 (1.29, 15.91) | 33.40 (6.95, 80.75) | 739.088398 | 0.41 (0.13, 1.66) | 0.96 (0.20, 2.33) | 2.775 (1.090, 4.488) |
| Iraq | 0.07 (0.01, 0.20) | 0.21 (0.02, 1.30) | 185.603176 | 0.02 (0.00, 0.05) | 0.02 (0.00, 0.13) | -0.129 (-2.180, 1.965) |
| Ireland | 0.06 (0.03, 0.12) | 0.11 (0.02, 0.31) | 86.2199902 | 0.02 (0.01, 0.04) | 0.02 (0.00, 0.06) | -0.061 (-1.692, 1.598) |
| Israel | 9.91 (4.45, 18.78) | 8.13 (2.17, 16.49) | -17.9979072 | 3.25 (1.46, 6.17) | 1.08 (0.29, 2.20) | -3.573 (-5.599, -1.504) |
| Italy | 27.84 (5.18, 90.28) | 71.43 (22.21, 194.15) | 156.597646 | 0.50 (0.09, 1.63) | 0.69 (0.22, 1.89) | 1.363 (-0.214, 2.965) |
| Jamaica | 6.76 (3.48, 15.38) | 4.69 (2.22, 8.28) | -30.6809141 | 5.81 (2.99, 13.22) | 2.65 (1.26, 4.69) | -2.593 (-4.544, -0.602) |
| Japan | 6.21 (4.14, 8.98) | 30.02 (9.71, 48.52) | 383.356476 | 0.06 (0.04, 0.09) | 0.11 (0.04, 0.18) | 1.833 (0.447, 3.237) |
| Jordan | 0.00 (0.00, 0.00) | 0.01 (0.00, 0.01) | 153.788525 | 0.00 (0.00, 0.01) | 0.00 (0.00, 0.01) | -2.977 (-5.666, -0.213) |
| Kazakhstan | 0.04 (0.02, 0.06) | 0.03 (0.00, 0.12) | -9.82075722 | 0.01 (0.00, 0.01) | 0.00 (0.00, 0.02) | -0.984 (-1.647, -0.316) |
| Kenya | 1829.77 (1470.84, 2241.56) | 461.21 (338.51, 624.40) | -74.7941417 | 494.74 (397.69, 606.08) | 52.11 (38.25, 70.55) | -7.568 (-7.833, -7.303) |
| Kiribati | 0.02 (0.01, 0.04) | 0.01 (0.00, 0.02) | -57.7420521 | 1.68 (0.84, 3.10) | 0.44 (0.06, 1.06) | -4.661 (-5.472, -3.842) |
| Kuwait | 0.02 (0.01, 0.03) | 0.00 (0.00, 0.00) | -90.3312934 | 0.10 (0.06, 0.15) | 0.00 (0.00, 0.00) | -12.665 (-15.489, -9.745) |
| Kyrgyzstan | 0.45 (0.21, 0.98) | 1.50 (0.52, 2.88) | 234.568549 | 0.29 (0.14, 0.64) | 0.83 (0.29, 1.59) | 3.835 (2.755, 4.927) |
| Lao People's Democratic Republic | 0.45 (0.00, 3.49) | 4.21 (0.02, 29.58) | 829.439907 | 0.51 (0.00, 3.90) | 2.34 (0.01, 16.42) | 2.308 (-2.010, 6.816) |
| Latvia | 0.35 (0.24, 0.60) | 0.06 (0.01, 0.17) | -81.7991302 | 0.17 (0.11, 0.29) | 0.02 (0.00, 0.06) | -7.516 (-9.689, -5.290) |
| Lebanon | 2.47 (0.00, 16.78) | 1.28 (0.00, 8.71) | -48.3549279 | 2.44 (0.00, 16.57) | 0.40 (0.00, 2.72) | -7.274 (-8.489, -6.042) |
| Lesotho | 90.45 (51.91, 138.04) | 222.85 (132.34, 342.02) | 146.370262 | 193.66 (111.15, 295.54) | 409.16 (242.98, 627.96) | 2.582 (2.002, 3.166) |
| Liberia | 17.44 (3.02, 44.19) | 15.46 (7.36, 29.32) | -11.3160722 | 29.86 (5.18, 75.67) | 18.34 (8.73, 34.78) | -1.632 (-2.409, -0.848) |
| Libya | 0.33 (0.00, 2.17) | 0.54 (0.00, 3.96) | 62.8737179 | 0.39 (0.00, 2.54) | 0.23 (0.00, 1.71) | -2.524 (-4.306, -0.708) |
| Lithuania | 0.01 (0.00, 0.02) | 0.00 (0.00, 0.00) | -85.2826767 | 0.00 (0.00, 0.01) | 0.00 (0.00, 0.00) | -7.632 (-9.470, -5.757) |
| Luxembourg | 0.72 (0.39, 1.45) | 0.05 (0.02, 0.09) | -93.2295686 | 2.12 (1.15, 4.23) | 0.08 (0.03, 0.15) | -10.808 (-11.387, -10.224) |
| Madagascar | 0.39 (0.08, 0.90) | 29.77 (10.90, 60.36) | 7529.12242 | 0.17 (0.03, 0.40) | 8.03 (2.94, 16.28) | 14.139 (13.464, 14.817) |
| Malawi | 842.72 (533.25, 1226.94) | 241.93 (130.18, 390.74) | -71.2917723 | 529.51 (335.06, 770.92) | 75.39 (40.57, 121.77) | -6.553 (-6.750, -6.356) |
| Malaysia | 10.57 (4.46, 24.70) | 2.02 (0.43, 4.68) | -80.8528956 | 2.51 (1.06, 5.87) | 0.16 (0.03, 0.36) | -9.779 (-10.428, -9.126) |
| Maldives | 0.00 (0.00, 0.00) | 0.00 (0.00, 0.01) | 104.545724 | 0.06 (0.05, 0.09) | 0.03 (0.01, 0.06) | -2.755 (-3.223, -2.286) |
| Mali | 51.55 (12.17, 121.60) | 49.17 (21.54, 96.55) | -4.61298964 | 29.19 (6.89, 68.84) | 13.10 (5.74, 25.72) | -2.661 (-3.452, -1.864) |
| Malta | 0.08 (0.06, 0.10) | 0.37 (0.15, 0.63) | 351.339833 | 0.33 (0.23, 0.42) | 0.56 (0.22, 0.95) | 1.845 (1.312, 2.381) |
| Marshall Islands | 0.06 (0.00, 0.50) | 0.05 (0.00, 0.37) | -15.591089 | 8.29 (0.02, 66.00) | 4.84 (0.03, 33.54) | -7.902 (-13.636, -1.788) |
| Mauritania | 15.11 (0.01, 109.54) | 1.36 (0.00, 6.73) | -90.9733799 | 28.81 (0.01, 208.87) | 1.35 (0.00, 6.69) | -10.136 (-11.415, -8.838) |
| Mauritius | 0.05 (0.04, 0.08) | 0.96 (0.22, 2.54) | 1673.25614 | 0.16 (0.12, 0.24) | 1.06 (0.25, 2.81) | 5.592 (1.850, 9.471) |
| Mexico | 52.98 (39.40, 85.90) | 50.55 (26.31, 71.08) | -4.57584602 | 2.52 (1.88, 4.09) | 0.79 (0.41, 1.12) | -3.292 (-4.082, -2.496) |
| Micronesia (Federated States of) | 0.15 (0.00, 1.04) | 0.40 (0.00, 2.89) | 164.491289 | 7.31 (0.02, 50.77) | 17.44 (0.08, 127.42) | -3.159 (-12.795, 7.542) |
| Monaco | 0.05 (0.00, 0.23) | 0.07 (0.00, 0.45) | 33.9506562 | 1.09 (0.02, 4.67) | 1.04 (0.03, 6.49) | -0.150 (-1.070, 0.779) |
| Mongolia | 0.00 (0.00, 0.00) | 0.02 (0.00, 0.08) | 6442.06518 | 0.00 (0.00, 0.00) | 0.03 (0.00, 0.10) | 8.833 (3.444, 14.502) |
| Montenegro | 0.01 (0.01, 0.02) | 0.00 (0.00, 0.00) | -81.7797269 | 0.04 (0.03, 0.06) | 0.00 (0.00, 0.01) | -7.671 (-8.403, -6.933) |
| Morocco | 8.02 (0.01, 50.85) | 3.19 (0.00, 22.80) | -60.2089318 | 1.29 (0.00, 8.16) | 0.23 (0.00, 1.65) | -7.858 (-9.355, -6.336) |
| Mozambique | 177.85 (86.90, 313.43) | 1162.19 (583.65, 2150.63) | 553.451215 | 72.56 (35.45, 127.87) | 259.18 (130.16, 479.61) | 4.508 (4.260, 4.757) |
| Myanmar | 74.59 (39.21, 111.12) | 119.91 (59.98, 197.26) | 60.7677262 | 7.52 (3.95, 11.21) | 5.70 (2.85, 9.37) | -1.395 (-2.320, -0.462) |
| Namibia | 49.76 (32.00, 75.30) | 60.93 (36.58, 93.86) | 22.4651787 | 132.28 (85.07, 200.18) | 88.49 (53.12, 136.29) | -1.424 (-2.042, -0.801) |
| Nauru | 0.00 (0.00, 0.00) | 0.00 (0.00, 0.01) | 606.147996 | 0.43 (0.02, 2.62) | 4.25 (0.10, 17.73) | 8.088 (6.997, 9.190) |
| Nepal | 0.42 (0.00, 2.58) | 8.58 (0.01, 53.22) | 1960.13299 | 0.11 (0.00, 0.71) | 0.78 (0.00, 4.86) | 4.542 (1.233, 7.959) |
| Netherlands | 2.93 (1.85, 4.31) | 7.47 (2.49, 14.15) | 155.04749 | 0.23 (0.14, 0.34) | 0.32 (0.11, 0.61) | 0.843 (-1.971, 3.738) |
| New Zealand | 0.38 (0.16, 0.60) | 2.30 (0.35, 5.18) | 498.328871 | 0.16 (0.06, 0.24) | 0.44 (0.07, 1.00) | 3.742 (2.267, 5.239) |
| Nicaragua | 0.06 (0.04, 0.07) | 10.33 (2.20, 20.85) | 18353.0646 | 0.08 (0.05, 0.10) | 4.80 (1.02, 9.69) | 15.306 (14.006, 16.620) |
| Niger | 22.03 (9.31, 42.56) | 12.51 (4.66, 25.34) | -43.1902588 | 21.29 (9.00, 41.14) | 4.03 (1.50, 8.17) | -5.750 (-6.337, -5.160) |
| Nigeria | 752.11 (471.67, 1135.81) | 986.52 (757.21, 1340.36) | 31.1679477 | 36.75 (23.05, 55.50) | 27.94 (21.44, 37.96) | -0.960 (-1.574, -0.342) |
| Niue | 0.00 (0.00, 0.00) | 0.00 (0.00, 0.02) | 962.637634 | 0.27 (0.01, 1.59) | 3.32 (0.08, 13.94) | 8.970 (7.487, 10.473) |
| North Macedonia | 0.00 (0.00, 0.00) | 0.00 (0.00, 0.01) | 50.7365493 | 0.00 (0.00, 0.00) | 0.00 (0.00, 0.00) | -1.394 (-2.321, -0.457) |
| Northern Mariana Islands | 0.00 (0.00, 0.03) | 0.01 (0.00, 0.02) | 79.1679277 | 0.86 (0.10, 5.25) | 0.47 (0.12, 1.45) | -2.098 (-4.309, 0.164) |
| Norway | 6.28 (3.34, 12.47) | 0.94 (0.30, 2.05) | -85.0705759 | 1.29 (0.68, 2.55) | 0.14 (0.05, 0.31) | -7.146 (-8.766, -5.498) |
| Oman | 0.05 (0.04, 0.07) | 0.14 (0.04, 0.39) | 167.168401 | 0.23 (0.15, 0.33) | 0.26 (0.07, 0.72) | 0.590 (-1.267, 2.481) |
| Pakistan | 33.13 (0.02, 187.99) | 37.05 (0.04, 232.75) | 11.808316 | 1.12 (0.00, 6.38) | 0.82 (0.00, 5.15) | -1.533 (-3.216, 0.179) |
| Palau | 0.00 (0.00, 0.01) | 0.03 (0.00, 0.14) | 1483.87546 | 0.43 (0.02, 2.62) | 4.05 (0.10, 17.39) | 8.038 (7.219, 8.862) |
| Palestine | 0.00 (0.00, 0.00) | 0.00 (0.00, 0.00) | -10.0229259 | 0.00 (0.00, 0.01) | 0.00 (0.00, 0.00) | -3.288 (-4.464, -2.097) |
| Panama | 7.38 (5.57, 10.78) | 4.25 (1.95, 7.79) | -42.3887881 | 9.03 (6.82, 13.20) | 1.74 (0.80, 3.18) | -5.526 (-6.198, -4.849) |
| Papua New Guinea | 0.61 (0.02, 1.90) | 45.84 (0.14, 169.46) | 7375.41207 | 0.94 (0.03, 2.91) | 27.92 (0.08, 103.21) | 12.576 (11.613, 13.547) |
| Paraguay | 0.61 (0.20, 2.52) | 5.73 (1.31, 13.17) | 840.605886 | 0.52 (0.17, 2.17) | 1.97 (0.45, 4.54) | 4.787 (3.594, 5.994) |
| Peru | 13.01 (8.30, 16.60) | 60.06 (16.51, 126.39) | 361.579727 | 2.18 (1.39, 2.78) | 3.25 (0.89, 6.84) | 1.057 (-0.023, 2.150) |
| Philippines | 0.00 (0.00, 0.00) | 20.46 (11.67, 30.90) | - | 2.09 (10.51, 0.20) | 0.61 (0.35, 0.92) | -1.600 (-8.209, 5.486) |
| Poland | 0.30 (0.20, 0.47) | 1.48 (0.31, 3.26) | 393.711395 | 0.01 (0.01, 0.02) | 0.03 (0.01, 0.07) | 3.475 (2.758, 4.197) |
| Portugal | 37.76 (20.74, 68.96) | 4.31 (0.99, 8.80) | -88.5744334 | 4.38 (2.40, 7.99) | 0.25 (0.06, 0.52) | -9.712 (-11.014, -8.391) |
| Puerto Rico | 35.97 (25.16, 55.56) | 7.53 (1.38, 16.93) | -79.0652254 | 15.99 (11.18, 24.70) | 1.48 (0.27, 3.34) | -7.797 (-8.863, -6.718) |
| Qatar | 0.00 (0.00, 0.00) | 0.00 (0.00, 0.00) | -38.6468173 | 0.00 (0.00, 0.01) | 0.00 (0.00, 0.00) | -8.071 (-9.953, -6.150) |
| Republic of Korea | 6.39 (0.00, 17.45) | 52.95 (5.28, 159.04) | 728.735953 | 0.50 (0.00, 1.37) | 1.01 (0.10, 3.03) | 2.110 (0.503, 3.743) |
| Republic of Moldova | 0.40 (0.09, 1.77) | 0.78 (0.22, 1.55) | 95.2610685 | 0.19 (0.04, 0.84) | 0.24 (0.07, 0.48) | 0.008 (-4.585, 4.823) |
| Romania | 5.00 (3.48, 7.38) | 1.96 (0.46, 5.71) | -60.7809548 | 0.35 (0.24, 0.51) | 0.08 (0.02, 0.23) | -5.840 (-8.777, -2.809) |
| Russian Federation | 5.93 (4.00, 8.64) | 26.03 (3.05, 60.33) | 338.860536 | 0.06 (0.04, 0.09) | 0.19 (0.02, 0.44) | 4.308 (-0.178, 8.996) |
| Rwanda | 169.70 (40.77, 375.91) | 33.31 (16.56, 61.54) | -80.3727078 | 138.38 (33.25, 306.53) | 14.25 (7.08, 26.32) | -7.593 (-7.883, -7.301) |
| Saint Kitts and Nevis | 0.37 (0.00, 1.32) | 0.96 (0.01, 3.46) | 160.042213 | 13.85 (0.12, 49.37) | 36.04 (0.27, 129.63) | 3.354 (2.733, 3.978) |
| Saint Lucia | 0.56 (0.41, 0.85) | 0.23 (0.04, 0.52) | -59.7173985 | 10.63 (7.77, 16.09) | 1.83 (0.34, 4.19) | -6.187 (-7.799, -4.546) |
| Saint Vincent and the Grenadines | 1.20 (0.89, 1.77) | 0.44 (0.11, 1.08) | -63.5286729 | 27.27 (20.22, 40.09) | 5.85 (1.41, 14.45) | -5.436 (-7.455, -3.374) |
| Samoa | 0.31 (0.00, 2.05) | 0.27 (0.00, 1.86) | -11.4808314 | 7.45 (0.02, 49.79) | 4.01 (0.02, 27.48) | -4.965 (-9.185, -0.549) |
| San Marino | 0.03 (0.00, 0.14) | 0.04 (0.00, 0.27) | 34.4429223 | 1.49 (0.03, 6.42) | 0.97 (0.02, 6.08) | -1.466 (-2.317, -0.607) |
| Sao Tome and Principe | 0.08 (0.01, 0.43) | 0.01 (0.00, 0.04) | -85.3230688 | 2.49 (0.23, 12.85) | 0.27 (0.00, 0.93) | -7.621 (-9.229, -5.985) |
| Saudi Arabia | 3.13 (0.67, 7.92) | 5.69 (0.64, 32.18) | 81.8712476 | 1.27 (0.27, 3.22) | 1.17 (0.13, 6.61) | -0.122 (-0.453, 0.211) |
| Senegal | 24.54 (12.88, 41.83) | 19.68 (10.11, 35.57) | -19.7838859 | 16.22 (8.52, 27.66) | 5.69 (2.92, 10.28) | -3.584 (-4.029, -3.136) |
| Serbia | 0.39 (0.00, 0.66) | 0.21 (0.04, 0.47) | -44.9159177 | 0.07 (0.00, 0.13) | 0.02 (0.00, 0.05) | -4.604 (-8.491, -0.551) |
| Seychelles | 0.11 (0.05, 0.28) | 0.01 (0.01, 0.02) | -90.4061685 | 3.31 (1.47, 8.20) | 0.21 (0.10, 0.35) | -8.680 (-10.209, -7.125) |
| Sierra Leone | 14.62 (3.47, 36.86) | 52.22 (24.41, 100.78) | 257.109306 | 14.31 (3.40, 36.07) | 31.60 (14.77, 60.98) | 2.704 (2.487, 2.922) |
| Singapore | 17.02 (8.85, 29.09) | 7.74 (2.97, 14.35) | -54.5406881 | 16.26 (8.45, 27.79) | 1.85 (0.71, 3.43) | -7.502 (-10.125, -4.802) |
| Slovakia | 0.05 (0.00, 0.48) | 0.02 (0.00, 0.04) | -70.7397559 | 0.02 (0.00, 0.15) | 0.00 (0.00, 0.01) | -5.459 (-9.980, -0.711) |
| Slovenia | 0.01 (0.00, 0.05) | 0.07 (0.03, 0.13) | 597.430573 | 0.01 (0.00, 0.04) | 0.02 (0.01, 0.04) | 4.244 (2.918, 5.587) |
| Solomon Islands | 0.35 (0.00, 2.51) | 0.48 (0.00, 3.38) | 37.2730897 | 8.27 (0.02, 59.08) | 4.69 (0.03, 32.85) | -2.317 (-3.585, -1.032) |
| Somalia | 4.28 (0.61, 14.93) | 13.02 (5.75, 25.56) | 203.974242 | 5.27 (0.75, 18.38) | 5.72 (2.53, 11.23) | 0.181 (-0.593, 0.961) |
| South Africa | 479.39 (373.18, 621.99) | 4488.67 (3445.52, 5893.38) | 836.334866 | 46.56 (36.25, 60.41) | 207.81 (159.51, 272.84) | 5.318 (4.492, 6.149) |
| South Sudan | 25.03 (2.65, 94.61) | 66.88 (9.75, 198.63) | 167.162055 | 22.26 (2.36, 84.13) | 43.48 (6.34, 129.15) | 2.402 (1.957, 2.849) |
| Spain | 30.65 (16.75, 46.63) | 8.61 (0.57, 29.23) | -71.9161586 | 0.89 (0.49, 1.35) | 0.13 (0.01, 0.44) | -6.333 (-7.800, -4.844) |
| Sri Lanka | 0.32 (0.02, 1.79) | 0.13 (0.02, 0.35) | -58.7561073 | 0.06 (0.01, 0.36) | 0.01 (0.00, 0.03) | -6.497 (-7.943, -5.029) |
| Sudan | 24.22 (2.71, 85.17) | 107.04 (14.45, 432.43) | 342.039135 | 5.15 (0.58, 18.10) | 12.77 (1.72, 51.57) | 3.164 (2.883, 3.446) |
| Suriname | 4.05 (1.50, 7.77) | 1.02 (0.35, 2.39) | -74.8436926 | 31.42 (11.66, 60.30) | 3.23 (1.10, 7.59) | -7.399 (-10.721, -3.954) |
| Sweden | 5.64 (3.06, 9.80) | 0.79 (0.12, 2.07) | -85.9012756 | 0.52 (0.28, 0.90) | 0.05 (0.01, 0.13) | -7.737 (-10.000, -5.416) |
| Switzerland | 14.50 (7.59, 22.51) | 10.49 (1.67, 21.03) | -27.6646186 | 2.08 (1.09, 3.22) | 0.88 (0.14, 1.76) | -3.522 (-6.696, -0.239) |
| Syrian Arab Republic | 0.03 (0.02, 0.03) | 0.02 (0.01, 0.04) | -6.84201123 | 0.01 (0.01, 0.01) | 0.00 (0.00, 0.01) | -3.252 (-17.077, 12.879) |
| Taiwan (Province of China) | 6.08 (3.27, 10.03) | 20.35 (6.29, 43.60) | 234.794042 | 0.83 (0.45, 1.37) | 0.91 (0.28, 1.95) | -0.425 (-4.906, 4.266) |
| Tajikistan | 0.01 (0.00, 0.02) | 0.01 (0.00, 0.04) | 24.5629345 | 0.01 (0.00, 0.01) | 0.01 (0.00, 0.03) | 0.595 (-0.517, 1.718) |
| Thailand | 195.01 (82.48, 452.64) | 25.38 (6.84, 70.02) | -86.9830884 | 12.75 (5.39, 29.60) | 0.45 (0.12, 1.24) | -10.777 (-13.195, -8.292) |
| Timor-Leste | 10.95 (0.05, 83.61) | 2.42 (0.01, 18.69) | -77.8484127 | 126.64 (0.55, 967.36) | 6.14 (0.04, 47.30) | -16.998 (-22.860, -10.690) |
| Togo | 31.25 (9.00, 72.27) | 27.40 (13.94, 49.20) | -12.3191683 | 60.70 (17.48, 140.41) | 19.49 (9.91, 35.00) | -4.022 (-4.527, -3.514) |
| Tokelau | 0.00 (0.00, 0.00) | 0.00 (0.00, 0.01) | 658.85591 | 0.40 (0.02, 2.38) | 4.04 (0.10, 17.74) | 8.197 (6.526, 9.894) |
| Tonga | 0.06 (0.01, 0.38) | 0.06 (0.01, 0.21) | -2.21667863 | 2.34 (0.24, 14.60) | 1.36 (0.16, 4.83) | -2.032 (-3.683, -0.353) |
| Trinidad and Tobago | 2.69 (1.90, 3.76) | 0.30 (0.08, 0.73) | -88.9599904 | 5.70 (4.03, 7.96) | 0.28 (0.07, 0.70) | -9.904 (-11.342, -8.442) |
| Tunisia | 0.62 (0.01, 3.18) | 0.93 (0.03, 6.76) | 49.1542069 | 0.26 (0.00, 1.31) | 0.14 (0.01, 1.03) | -1.923 (-3.554, -0.264) |
| Turkey | 1.59 (0.00, 4.97) | 3.15 (1.50, 5.31) | 98.4070144 | 0.10 (0.00, 0.31) | 0.07 (0.03, 0.11) | -2.890 (-4.739, -1.004) |
| Turkmenistan | 0.00 (0.00, 0.01) | 0.00 (0.00, 0.00) | -97.9178482 | 0.00 (0.00, 0.01) | 0.00 (0.00, 0.00) | -14.734 (-17.675, -11.688) |
| Tuvalu | 0.00 (0.00, 0.01) | 0.02 (0.00, 0.09) | 1835.11156 | 0.37 (0.02, 2.29) | 4.13 (0.10, 18.26) | 8.699 (8.285, 9.114) |
| Uganda | 848.78 (490.94, 1359.15) | 382.76 (179.54, 674.07) | -54.9042554 | 286.93 (165.96, 459.47) | 64.20 (30.11, 113.06) | -5.114 (-5.425, -4.802) |
| Ukraine | 0.37 (0.28, 0.58) | 0.68 (0.04, 2.02) | 86.8583943 | 0.01 (0.01, 0.01) | 0.01 (0.00, 0.04) | 1.531 (-0.429, 3.530) |
| United Arab Emirates | 0.04 (0.00, 0.26) | 20.26 (0.01, 173.70) | 53820.0726 | 0.40 (0.00, 2.79) | 50.26 (0.02, 430.82) | 15.564 (6.116, 25.853) |
| United Kingdom | 22.32 (14.81, 38.16) | 20.45 (10.75, 35.37) | -8.34210201 | 0.36 (0.24, 0.61) | 0.23 (0.12, 0.40) | -1.516 (-2.827, -0.187) |
| United Republic of Tanzania | 1952.90 (1041.25, 3138.93) | 421.65 (167.96, 798.15) | -78.409081 | 396.87 (211.60, 637.90) | 38.49 (15.33, 72.87) | -7.783 (-8.016, -7.550) |
| United States of America | 338.38 (180.98, 482.65) | 437.36 (73.01, 845.61) | 29.2505075 | 1.59 (0.85, 2.26) | 1.22 (0.20, 2.35) | -0.513 (-2.389, 1.399) |
| United States Virgin Islands | 0.51 (0.23, 1.23) | 0.61 (0.17, 1.35) | 19.6659443 | 12.63 (5.61, 30.12) | 4.98 (1.37, 10.92) | -3.079 (-4.829, -1.296) |
| Uruguay | 71.14 (23.18, 218.83) | 12.86 (5.01, 25.96) | -81.9282033 | 28.96 (9.44, 89.10) | 3.48 (1.36, 7.03) | -7.311 (-9.862, -4.689) |
| Uzbekistan | 0.93 (0.18, 1.32) | 0.48 (0.01, 2.27) | -48.1914082 | 0.17 (0.03, 0.24) | 0.09 (0.00, 0.41) | -2.653 (-4.999, -0.249) |
| Vanuatu | 0.22 (0.00, 1.79) | 0.39 (0.00, 2.83) | 73.0979889 | 8.06 (0.02, 64.43) | 4.89 (0.03, 35.65) | -10.653 (-20.055, -0.145) |
| Venezuela (Bolivarian Republic of) | 29.26 (14.20, 56.25) | 62.66 (17.07, 127.75) | 114.152415 | 6.16 (2.99, 11.83) | 4.10 (1.12, 8.35) | -0.836 (-2.565, 0.923) |
| Viet Nam | 30.88 (8.59, 70.33) | 46.74 (6.08, 122.78) | 51.3716064 | 1.52 (0.42, 3.45) | 1.13 (0.15, 2.98) | -1.173 (-2.731, 0.409) |
| Yemen | 2.39 (0.00, 16.04) | 2.23 (0.00, 15.86) | -6.70419925 | 1.23 (0.00, 8.25) | 0.39 (0.00, 2.80) | -4.834 (-5.740, -3.920) |
| Zambia | 657.33 (382.64, 1037.11) | 264.36 (129.33, 480.47) | -59.7824543 | 565.62 (329.25, 892.41) | 96.12 (47.03, 174.70) | -5.905 (-6.106, -5.705) |
| Zimbabwe | 1166.61 (165.85, 2557.83) | 226.87 (113.39, 398.61) | -80.5527512 | 650.09 (92.42, 1425.36) | 78.38 (39.17, 137.71) | -7.089 (-7.659, -6.515) |

Note: ASR: age-standardized rates; AAPC, average annual percentage change; CI, confidence interval; UI, uncertainty interval

**Supplementary Table S3**. The HIV/AIDs incidence and its trends among older adults by region and sex level.

|  | Female | | | Male | | |
| --- | --- | --- | --- | --- | --- | --- |
|  | 1990 (95% UI) | 2019 (95% UI) | AAPC (95% CI) | 1990 (95% UI) | 2019 (95% UI) | AAPC (95% CI) |
| **Global** | 5.73 (4.60, 7.22) | 2.61 (2.07, 3.26) | -2.668 (-3.083, -2.252) | 10.82 (8.53, 13.97) | 4.03 (3.27, 5.04) | -3.443 (-3.800, -3.085) |
| **SDI region** |  |  |  |  |  |  |
| Low SDI | 75.04 (53.30, 99.43) | 14.14 (9.96, 19.33) | -5.623 (-5.783, -5.463) | 87.31 (58.13, 120.96) | 15.74 (11.00, 22.68) | -5.774 (-5.941, -5.606) |
| Low-middle SDI | 16.48 (12.27, 22.26) | 4.09 (3.13, 5.27) | -4.863 (-5.372, -4.350) | 23.06 (15.74, 33.28) | 5.61 (4.11, 7.58) | -4.838 (-5.300, -4.373) |
| Middle SDI | 2.94 (2.31, 4.18) | 4.89 (3.76, 6.39) | 1.874 (1.432, 2.318) | 5.72 (3.93, 10.13) | 6.48 (4.98, 8.12) | 0.714 (-0.308, 1.746) |
| High-middle SDI | 0.37 (0.24, 0.65) | 0.22 (0.13, 0.32) | -1.735 (-2.435, -1.030) | 1.56 (1.00, 2.87) | 0.96 (0.67, 1.28) | -1.898 (-3.637, -0.127) |
| High SDI | 0.44 (0.29, 0.66) | 0.15 (0.06, 0.26) | -3.454 (-5.014, -1.868) | 1.60 (1.15, 2.33) | 1.07 (0.49, 1.78) | -1.322 (-2.876, 0.258) |
| **GBD region** |  |  |  |  |  |  |
| Andean Latin America | 1.68 (0.71, 4.35) | 1.73 (0.40, 3.76) | 0.728 (-1.514, 3.021) | 3.50 (1.67, 7.97) | 4.06 (1.39, 8.35) | 0.409 (-0.436, 1.261) |
| Australasia | 0.05 (0.03, 0.07) | 0.03 (0.00, 0.14) | -1.618 (-3.079, -0.134) | 1.25 (0.73, 1.79) | 0.63 (0.32, 1.06) | -2.316 (-3.811, -0.797) |
| Caribbean | 19.65 (12.29, 30.05) | 3.58 (1.85, 6.47) | -5.861 (-6.778, -4.934) | 33.60 (20.98, 56.03) | 6.45 (3.46, 10.98) | -5.611 (-6.271, -4.946) |
| Central Asia | 0.00 (0.00, 0.01) | 0.07 (0.02, 0.15) | 11.412 (8.560, 14.338) | 0.20 (0.08, 0.35) | 0.12 (0.04, 0.32) | -1.405 (-2.264, -0.539) |
| Central Europe | 0.04 (0.03, 0.06) | 0.01 (0.00, 0.01) | -6.390 (-8.603, -4.124) | 0.17 (0.12, 0.25) | 0.09 (0.04, 0.17) | -2.320 (-4.763, 0.186) |
| Central Latin America | 1.27 (0.87, 2.19) | 0.40 (0.15, 0.70) | -4.036 (-5.699, -2.344) | 5.07 (3.57, 8.40) | 3.61 (1.71, 5.83) | -0.821 (-1.519, -0.117) |
| Central Sub-Saharan Africa | 125.89 (82.87, 180.24) | 24.02 (15.11, 36.54) | -5.571 (-5.731, -5.410) | 95.77 (56.98, 145.98) | 21.40 (12.95, 33.39) | -5.083 (-5.263, -4.902) |
| East Asia | 0.79 (0.33, 2.19) | 0.35 (0.15, 0.51) | -5.791 (-7.868, -3.668) | 2.90 (1.40, 7.15) | 2.64 (1.26, 4.01) | -0.962 (-3.630, 1.781) |
| Eastern Europe | 0.02 (0.01, 0.04) | 0.12 (0.01, 0.27) | 6.457 (1.527, 11.626) | 0.12 (0.07, 0.18) | 0.18 (0.02, 0.45) | 1.569 (-2.834, 6.171) |
| Eastern Sub-Saharan Africa | 221.08 (160.29, 285.36) | 44.80 (31.62, 60.74) | -5.397 (-5.600, -5.193) | 285.04 (195.98, 386.63) | 50.96 (34.44, 73.01) | -5.802 (-5.961, -5.643) |
| High-income Asia Pacific | 0.05 (0.03, 0.08) | 0.02 (0.01, 0.04) | -2.034 (-5.031, 1.057) | 0.61 (0.29, 1.09) | 0.62 (0.22, 1.37) | -1.010 (-3.724, 1.781) |
| High-income North America | 0.91 (0.50, 1.43) | 0.35 (0.06, 0.72) | -2.875 (-5.103, -0.594) | 2.47 (1.38, 3.39) | 2.05 (0.35, 4.05) | -0.253 (-2.190, 1.722) |
| North Africa and Middle East | 0.37 (0.08, 1.17) | 0.70 (0.22, 1.92) | 2.047 (1.279, 2.821) | 1.02 (0.21, 3.58) | 1.21 (0.29, 4.89) | -0.403 (-1.115, 0.315) |
| Oceania | 1.72 (0.26, 6.44) | 17.79 (0.38, 64.68) | 6.447 (3.394, 9.590) | 2.71 (0.52, 12.34) | 21.39 (0.39, 86.23) | 8.401 (4.424, 12.530) |
| South Asia | 1.31 (0.35, 4.49) | 0.21 (0.03, 0.54) | -6.198 (-6.863, -5.529) | 0.58 (0.23, 1.66) | 0.77 (0.18, 1.86) | 1.053 (0.382, 1.728) |
| Southeast Asia | 1.90 (0.86, 4.37) | 0.82 (0.29, 1.84) | -3.851 (-4.985, -2.703) | 9.03 (2.86, 26.14) | 1.54 (0.78, 2.84) | -5.981 (-7.755, -4.174) |
| Southern Latin America | 2.40 (0.83, 6.61) | 0.47 (0.20, 1.04) | -5.541 (-7.138, -3.917) | 5.51 (2.80, 12.97) | 2.37 (1.54, 3.28) | -2.838 (-4.166, -1.491) |
| Southern Sub-Saharan Africa | 112.68 (55.34, 190.40) | 179.55 (134.81, 236.00) | 1.642 (0.631, 2.662) | 195.63 (80.88, 370.56) | 216.87 (158.14, 296.72) | 0.352 (-0.484, 1.194) |
| Tropical Latin America | 0.70 (0.56, 0.91) | 2.64 (1.15, 4.50) | 4.776 (4.207, 5.348) | 1.56 (1.20, 2.03) | 7.93 (3.86, 12.99) | 5.827 (5.296, 6.362) |
| Western Europe | 0.32 (0.20, 0.51) | 0.12 (0.06, 0.19) | -3.595 (-4.342, -2.842) | 1.25 (0.86, 2.01) | 0.57 (0.36, 1.03) | -2.640 (-3.149, -2.128) |
| Western Sub-Saharan Africa | 43.24 (28.98, 61.84) | 20.95 (16.27, 26.86) | -2.577 (-2.847, -2.306) | 63.06 (41.51, 94.80) | 24.71 (19.01, 32.82) | -3.247 (-3.642, -2.850) |
| **WHO region** |  |  |  |  |  |  |
| African Region | 112.18 (83.71, 145.70) | 51.10 (40.54, 63.92) | -2.724 (-2.951, -2.495) | 152.36 (105.59, 204.05) | 51.24 (40.24, 65.78) | -3.710 (-4.121, -3.296) |
| Eastern Mediterranean Region | 0.62 (0.12, 1.70) | 0.97 (0.37, 2.56) | 1.610 (1.045, 2.178) | 1.45 (0.24, 4.53) | 1.66 (0.41, 5.62) | 0.157 (-1.307, 1.643) |
| European Region | 0.19 (0.13, 0.30) | 0.10 (0.05, 0.15) | -2.490 (-3.568, -1.400) | 0.84 (0.59, 1.33) | 0.42 (0.27, 0.71) | -2.508 (-3.028, -1.985) |
| Region of the Americas | 1.67 (1.23, 2.27) | 0.94 (0.50, 1.42) | -2.002 (-2.718, -1.281) | 4.18 (3.20, 5.39) | 3.58 (2.00, 5.15) | -0.406 (-1.183, 0.377) |
| South-East Asia Region | 1.78 (0.69, 4.68) | 0.38 (0.12, 0.83) | -6.421 (-8.431, -4.368) | 3.38 (1.09, 10.39) | 0.92 (0.37, 1.86) | -3.871 (-4.431, -3.308) |
| Western Pacific Region | 0.59 (0.26, 1.57) | 0.31 (0.16, 0.46) | -2.027 (-4.025, 0.014) | 2.42 (1.28, 5.51) | 2.17 (1.13, 3.11) | -0.812 (-2.491, 0.896) |
| **World Bank Income region** |  |  |  |  |  |  |
| Low Income | 115.57 (77.37, 157.77) | 21.96 (15.57, 30.16) | -5.613 (-5.783, -5.442) | 151.02 (94.51, 222.11) | 27.38 (18.97, 39.48) | -5.753 (-5.920, -5.585) |
| Lower Middle Income | 8.86 (7.17, 11.35) | 2.78 (2.14, 3.62) | -3.951 (-4.307, -3.594) | 13.89 (10.53, 18.26) | 3.60 (2.64, 4.96) | -4.597 (-4.926, -4.266) |
| Upper Middle Income | 1.68 (1.29, 2.55) | 3.18 (2.41, 4.19) | 2.369 (1.821, 2.919) | 3.99 (2.83, 7.03) | 5.04 (3.84, 6.26) | 0.829 (-0.257, 1.926) |
| High Income | 0.51 (0.35, 0.78) | 0.16 (0.08, 0.26) | -3.841 (-5.098, -2.568) | 1.70 (1.25, 2.42) | 1.06 (0.56, 1.66) | -1.564 (-2.909, -0.201) |

Note: AAPC, average annual percentage change; CI, confidence interval; UI, uncertainty interval

**Supplementary Table S4**. The incident cases of HIV/AIDs and its changes among older adults by region and sex level.

|  | Female | | | Male | | |
| --- | --- | --- | --- | --- | --- | --- |
|  | 1990 (95% UI) | 2019 (95% UI) | Percentage change (%) | 1990 (95% UI) | 2019 (95% UI) | Percentage change (%) |
| Global | 6831.20 (5486.79, 8613.31) | 6794.85 (5392.87, 8497.48) | -0.53 | 8894.26 (7016.11, 11483.96) | 8206.72 (6643.05, 10249.61) | -7.73 |
| **SDI region** |  |  |  |  |  |  |
| Low SDI | 3631.63 (2579.62, 4812.11) | 1666.40 (1173.68, 2278.35) | -54.11 | 4200.97 (2796.74, 5820.12) | 1692.15 (1182.50, 2438.48) | -59.72 |
| Low-middle SDI | 2137.57 (1591.12, 2887.96) | 1508.13 (1151.94, 1939.77) | -29.45 | 2824.30 (1928.34, 4076.55) | 1764.35 (1290.43, 2381.69) | -37.53 |
| Middle SDI | 741.73 (582.57, 1054.54) | 3352.24 (2576.32, 4379.64) | 351.95 | 1143.10 (784.67, 2023.91) | 3683.62 (2827.81, 4612.23) | 222.25 |
| High-middle SDI | 129.06 (83.44, 225.41) | 153.44 (94.21, 226.21) | 18.89 | 320.91 (204.57, 589.19) | 470.09 (328.76, 629.09) | 46.49 |
| High SDI | 183.12 (119.74, 273.81) | 109.86 (43.43, 192.56) | -40.01 | 393.04 (284.07, 573.07) | 590.41 (268.63, 982.78) | 50.22 |
| **GBD region** |  |  |  |  |  |  |
| Andean Latin America | 9.01 (3.82, 23.30) | 28.63 (6.57, 62.42) | 217.73 | 16.95 (8.09, 38.66) | 60.23 (20.68, 123.95) | 255.37 |
| Australasia | 0.41 (0.23, 0.61) | 0.55 (0.08, 2.49) | 32.37 | 7.32 (4.25, 10.48) | 9.53 (4.81, 16.09) | 30.23 |
| Caribbean | 155.42 (97.23, 237.69) | 60.62 (31.37, 109.46) | -61.00 | 234.89 (146.67, 391.77) | 89.66 (48.13, 152.77) | -61.83 |
| Central Asia | 0.05 (0.02, 0.15) | 1.26 (0.29, 2.67) | 2501.20 | 1.44 (0.57, 2.47) | 1.30 (0.38, 3.33) | -10.06 |
| Central Europe | 2.02 (1.36, 3.15) | 0.55 (0.15, 1.28) | -72.86 | 4.97 (3.49, 7.37) | 4.61 (1.92, 9.07) | -7.29 |
| Central Latin America | 27.31 (18.74, 47.16) | 28.72 (10.77, 50.00) | 5.17 | 97.17 (68.44, 160.90) | 211.17 (100.02, 340.56) | 117.33 |
| Central Sub-Saharan Africa | 550.28 (362.24, 787.84) | 285.84 (179.79, 434.83) | -48.06 | 382.75 (227.75, 583.43) | 162.98 (98.65, 254.24) | -57.42 |
| East Asia | 178.38 (74.78, 493.03) | 214.89 (89.18, 315.18) | 20.47 | 494.66 (238.84, 1219.65) | 1343.33 (639.56, 2036.30) | 171.57 |
| Eastern Europe | 2.56 (1.14, 4.08) | 16.40 (2.04, 37.78) | 541.13 | 4.60 (2.91, 6.84) | 11.15 (1.03, 28.38) | 142.54 |
| Eastern Sub-Saharan Africa | 3530.12 (2559.45, 4556.54) | 1668.12 (1177.28, 2261.55) | -52.75 | 4425.97 (3043.03, 6003.37) | 1567.44 (1059.15, 2245.50) | -64.59 |
| High-income Asia Pacific | 3.48 (1.94, 5.71) | 4.75 (1.76, 7.36) | 36.54 | 26.18 (12.64, 46.96) | 86.05 (30.70, 191.71) | 228.70 |
| High-income North America | 130.99 (72.00, 206.04) | 79.99 (14.17, 163.78) | -38.94 | 221.08 (123.68, 303.04) | 360.15 (61.77, 712.37) | 62.90 |
| North Africa and Middle East | 14.20 (3.03, 44.63) | 68.43 (21.29, 187.70) | 381.95 | 37.39 (7.51, 131.07) | 118.21 (28.64, 475.42) | 216.14 |
| Oceania | 0.94 (0.14, 3.52) | 23.35 (0.50, 84.89) | 2389.23 | 1.47 (0.28, 6.67) | 27.33 (0.50, 110.21) | 1764.58 |
| South Asia | 140.44 (37.07, 480.59) | 74.28 (11.66, 195.61) | -47.11 | 65.40 (26.07, 186.47) | 254.29 (61.04, 614.86) | 288.84 |
| Southeast Asia | 116.72 (52.63, 268.20) | 133.37 (47.57, 296.80) | 14.27 | 431.79 (137.00, 1250.29) | 178.17 (90.83, 329.17) | -58.74 |
| Southern Latin America | 37.45 (12.93, 102.93) | 14.53 (6.12, 32.24) | -61.20 | 59.07 (30.05, 139.18) | 50.63 (32.86, 70.02) | -14.28 |
| Southern Sub-Saharan Africa | 907.89 (445.88, 1534.13) | 2994.05 (2248.06, 3935.48) | 229.78 | 1026.80 (424.51, 1944.95) | 2133.51 (1555.73, 2918.98) | 107.78 |
| Tropical Latin America | 16.90 (13.48, 21.97) | 203.77 (88.64, 347.02) | 1106.03 | 30.14 (23.31, 39.25) | 449.37 (218.72, 735.98) | 1391.00 |
| Western Europe | 75.10 (48.15, 120.78) | 42.32 (20.78, 68.97) | -43.65 | 171.21 (117.28, 274.67) | 154.93 (98.48, 279.65) | -9.51 |
| Western Sub-Saharan Africa | 931.55 (624.27, 1332.17) | 850.44 (660.66, 1090.53) | -8.71 | 1153.01 (759.02, 1733.19) | 932.67 (717.53, 1238.42) | -19.11 |
| **WHO region** |  |  |  |  |  |  |
| African Region | 5915.08 (4414.16, 7682.35) | 5787.44 (4591.30, 7238.85) | -2.16 | 6983.28 (4839.39, 9352.48) | 4790.63 (3762.38, 6150.15) | -31.40 |
| Eastern Mediterranean Region | 24.35 (4.91, 66.95) | 84.10 (31.83, 222.28) | 245.40 | 63.01 (10.38, 196.45) | 151.57 (37.48, 513.22) | 140.54 |
| European Region | 79.89 (53.43, 125.37) | 60.79 (32.51, 93.13) | -23.90 | 183.44 (129.47, 290.22) | 174.68 (112.82, 298.02) | -4.78 |
| Region of the Americas | 361.90 (267.81, 492.06) | 411.27 (217.77, 620.90) | 13.64 | 623.84 (477.45, 804.55) | 1212.54 (676.99, 1742.51) | 94.37 |
| South-East Asia Region | 242.29 (93.81, 636.87) | 172.95 (55.19, 377.26) | -28.62 | 432.89 (139.61, 1330.56) | 359.45 (146.15, 727.22) | -16.97 |
| Western Pacific Region | 189.31 (84.36, 505.71) | 270.38 (136.18, 394.51) | 42.82 | 567.25 (300.28, 1288.25) | 1504.81 (783.09, 2151.20) | 165.28 |
| **World Bank Income region** |  |  |  |  |  |  |
| Low Income | 3791.73 (2538.40, 5176.18) | 1691.74 (1199.26, 2323.98) | -55.38 | 4436.08 (2776.10, 6524.30) | 1619.41 (1122.04, 2335.70) | -63.49 |
| Lower Middle Income | 2062.27 (1670.63, 2643.63) | 1673.63 (1288.10, 2181.28) | -18.85 | 2771.69 (2101.91, 3643.64) | 1839.65 (1350.76, 2535.50) | -33.63 |
| Upper Middle Income | 712.80 (547.33, 1076.91) | 3285.30 (2493.30, 4329.62) | 360.90 | 1159.42 (821.42, 2039.87) | 4043.41 (3083.50, 5029.32) | 248.75 |
| High Income | 256.32 (175.10, 390.77) | 139.39 (66.97, 228.57) | -45.62 | 515.13 (377.10, 731.30) | 698.10 (368.31, 1098.48) | 35.52 |

Note: UI, uncertainty interval


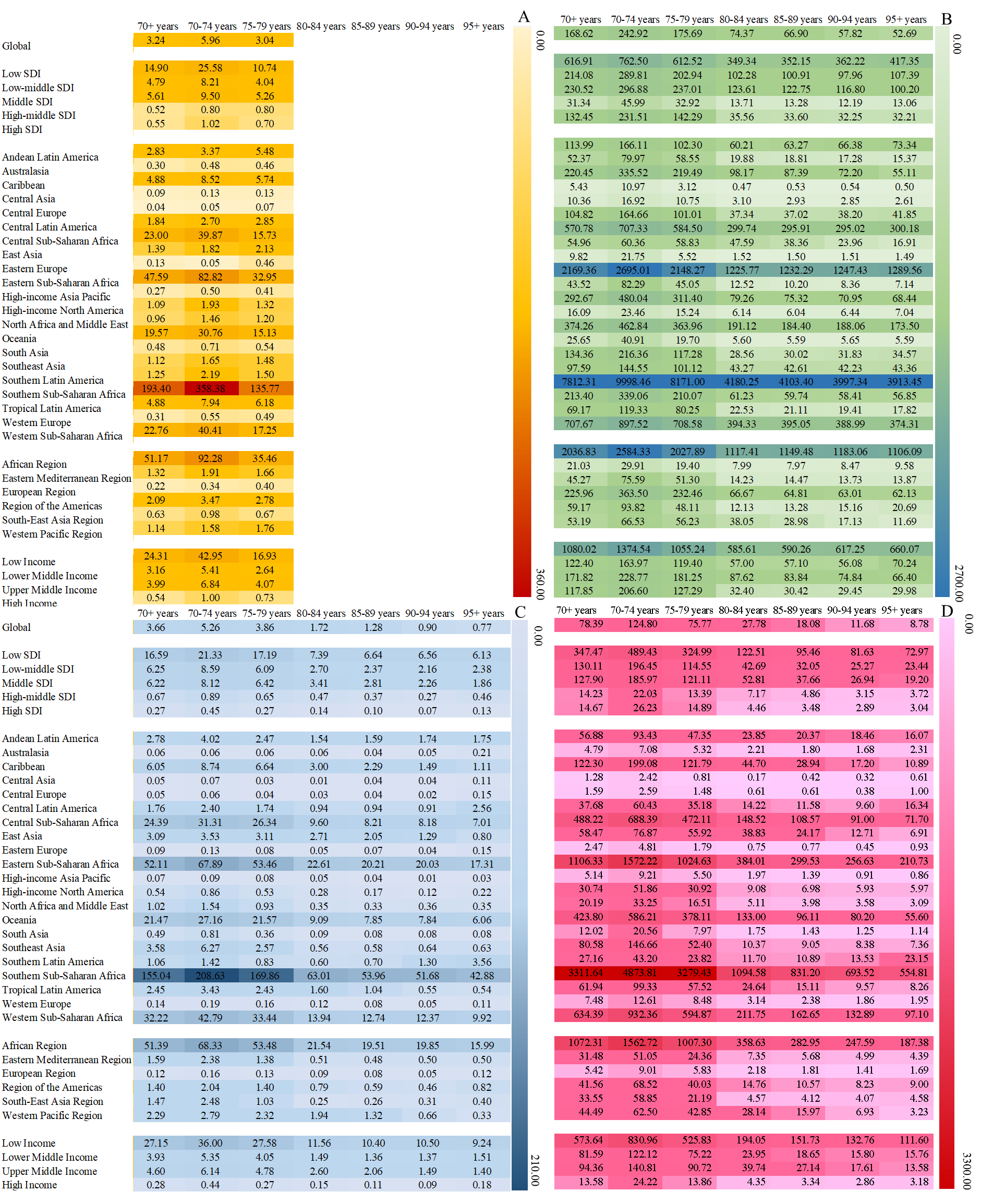


**Supplementary Figure S1**. The incidence, prevalence, mortality, and rate of DALYs in HIV/AIDs by age group at overall, SDI, GBD, WHO and World Bank income regional level in 2019.

DALY: disability adjusted life years; GBD: Global Burden of Diseases, Injuries, and Risk Factors Study; SDI: Sociodemographic Index; WHO: World Health Organization

**Supplementary Table S5**. Average annual percentage changes of incidence rate by region and age from 2010 to 2019.

|  | 70-74 years | 75-79 years |
| --- | --- | --- |
|  |  |  |
| Global | -2.628 (-3.230, -2.023) | -3.195 (-3.721, -2.667) |
| **SDI region** |  |  |
| Low SDI | -5.417 (-5.583, -5.250) | -5.626 (-5.864, -5.387) |
| Low-middle SDI | -4.418 (-4.610, -4.225) | -5.297 (-6.034, -4.554) |
| Middle SDI | 2.931 (2.281, 3.585) | -1.565 (-3.423, 0.328) |
| High-middle SDI | 1.057 (0.488, 1.629) | -2.641 (-3.974, -1.289) |
| High SDI | 0.120 (-1.194, 1.451) | -3.043 (-4.473, -1.592) |
| **GBD region** |  |  |
| Andean Latin America | 1.000 (0.377, 1.626) | -0.118 (-1.986, 1.785) |
| Australasia | -1.402 (-2.827, 0.044) | -1.619 (-3.069, -0.147) |
| Caribbean | -5.617 (-5.990, -5.242) | -5.625 (-6.570, -4.670) |
| Central Asia | 4.743 (3.726, 5.771) | -1.084 (-2.034, -0.126) |
| Central Europe | -2.185 (-4.820, 0.522) | -3.024 (-5.423, -0.564) |
| Central Latin America | 2.129 (1.723, 2.537) | -3.600 (-4.757, -2.428) |
| Central Sub-Saharan Africa | -4.760 (-4.930, -4.589) | -4.991 (-5.163, -4.819) |
| East Asia | 3.092 (1.225, 4.993) | -3.807 (-7.774, 0.330) |
| Eastern Europe | 0.186 (-3.794, 4.331) | 5.559 (-0.097, 11.535) |
| Eastern Sub-Saharan Africa | -5.265 (-5.427, -5.102) | -5.797 (-5.997, -5.596) |
| High-income Asia Pacific | 0.097 (-2.225, 2.474) | 0.030 (-1.327, 1.405) |
| High-income North America | 0.687 (-0.974, 2.375) | -2.158 (-4.696, 0.448) |
| North Africa and Middle East | 1.664 (1.210, 2.119) | -0.470 (-1.576, 0.648) |
| Oceania | 11.116 (10.316, 11.922) | -0.134 (-5.860, 5.941) |
| South Asia | 2.304 (1.082, 3.542) | -5.153 (-5.634, -4.671) |
| Southeast Asia | -0.444 (-1.677, 0.805) | -8.264 (-10.393, -6.084) |
| Southern Latin America | -1.541 (-2.193, -0.885) | -5.795 (-7.940, -3.599) |
| Southern Sub-Saharan Africa | 1.145 (0.127, 2.173) | 0.742 (-0.076, 1.567) |
| Tropical Latin America | 5.953 (5.269, 6.642) | 5.339 (4.252, 6.438) |
| Western Europe | -0.364 (-0.984, 0.260) | -3.775 (-4.848, -2.689) |
| Western Sub-Saharan Africa | -2.583 (-2.779, -2.387) | -2.816 (-3.129, -2.502) |
| **WHO region** |  |  |
| African Region | -2.841 (-3.071, -2.609) | -3.326 (-3.558, -3.093) |
| Eastern Mediterranean Region | 2.302 (1.825, 2.781) | -1.396 (-2.687, -0.087) |
| European Region | -0.201 (-0.971, 0.574) | -2.961 (-4.125, -1.783) |
| Region of the Americas | 0.004 (-0.566, 0.577) | -1.515 (-2.666, -0.349) |
| South-East Asia Region | 0.146 (-0.342, 0.635) | -8.128 (-9.340, -6.900) |
| Western Pacific Region | 2.981 (1.643, 4.337) | -2.750 (-5.581, 0.165) |
| **World Bank Income region** |  |  |
| Low Income | -5.317 (-5.482, -5.152) | -5.808 (-6.147, -5.469) |
| Lower Middle Income | -3.885 (-4.044, -3.727) | -5.021 (-5.324, -4.716) |
| Upper Middle Income | 3.412 (2.526, 4.305) | -0.989 (-2.615, 0.663) |
| High Income | 0.200 (-1.470, 1.899) | -3.285 (-4.468, -2.086) |

Note: data was presented as average annual percentage change and its 95% confidence interval


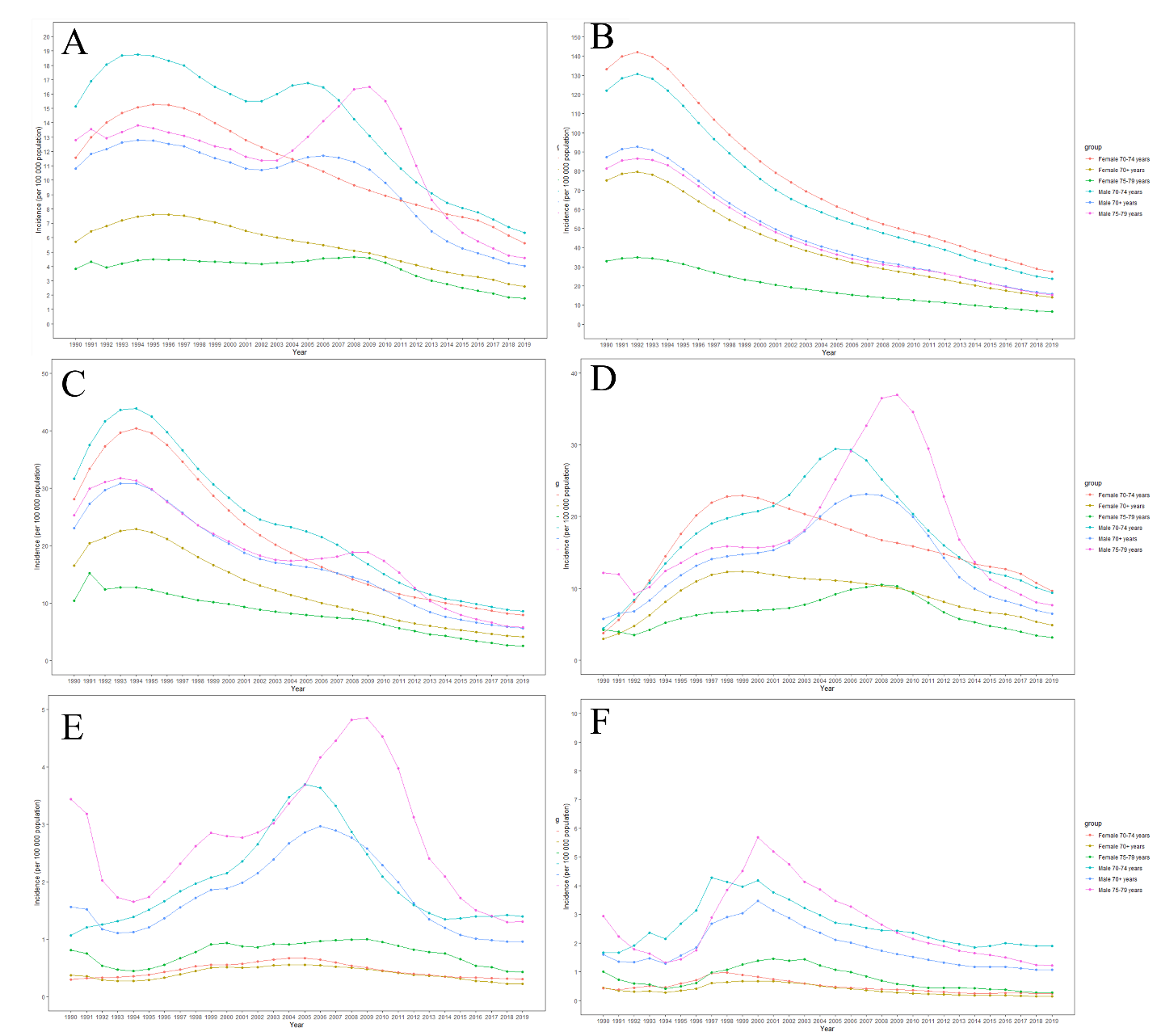


**Supplementary Figure S2**. Sex and age group distribution of HIV/AIDs incidence by SDI region from1990 to 2019.

(A) Global; (B) Low SDI; (C) Low-middle SDI; (D) Middle SDI; (E) High-middle SDI; (F) High SDI. SDI: Socio-demographic Index

**Supplementary Table S6**. The number of cases and prevalence of HIV/AIDs among older adults in 204 countries or territories from 1990 to 2019.

|  | Number |  |  | ASR (per 100,000) | |  |
| --- | --- | --- | --- | --- | --- | --- |
| Countries or territories | 1990 (95% UI) | 2019 (95% UI) | Percentage change (%) | 1990 (95% UI) | 2019 (95% UI) | AAPC (95% CI) |
| Afghanistan | 18.08 (0.73, 119.52) | 39.73 (0.77, 201.54) | 119.71 | 5.52 (0.22, 36.49) | 7.99 (0.16, 40.52) | 1.275 (1.104, 1.446) |
| Albania | 0.51 (0.41, 0.73) | 1.56 (1.10, 2.08) | 203.60 | 0.51 (0.41, 0.72) | 0.59 (0.42, 0.79) | 0.532 (-0.043, 1.109) |
| Algeria | 2.66 (0.58, 15.11) | 343.19 (23.54, 1404.21) | 12788.65 | 0.44 (0.10, 2.49) | 21.50 (1.48, 87.97) | 14.355 (13.995, 14.716) |
| American Samoa | 0.05 (0.02, 0.16) | 0.25 (0.12, 0.55) | 411.74 | 5.51 (1.97, 18.18) | 11.33 (5.50, 24.53) | 2.547 (2.095, 3.001) |
| Andorra | 0.99 (0.11, 4.23) | 8.62 (0.27, 48.18) | 767.38 | 33.84 (3.69, 143.97) | 101.77 (3.22, 568.76) | 3.926 (3.676, 4.175) |
| Angola | 42.33 (17.33, 87.55) | 3145.16 (2082.39, 4525.21) | 7330.55 | 32.07 (13.13, 66.34) | 831.78 (550.71, 1196.75) | 11.846 (11.570, 12.123) |
| Antigua and Barbuda | 3.62 (2.33, 6.38) | 6.09 (3.37, 8.96) | 68.33 | 99.78 (64.20, 176.10) | 115.51 (64.01, 170.01) | 0.692 (0.116, 1.271) |
| Argentina | 200.87 (146.49, 265.44) | 3103.80 (2529.45, 3715.96) | 1445.15 | 10.85 (7.91, 14.34) | 90.75 (73.96, 108.65) | 7.633 (7.496, 7.769) |
| Armenia | 0.18 (0.00, 0.32) | 6.52 (4.05, 10.80) | 3515.71 | 0.16 (0.00, 0.28) | 2.90 (1.80, 4.81) | 10.633 (8.915, 12.378) |
| Australia | 68.50 (50.25, 93.19) | 1617.69 (1192.71, 1971.09) | 2261.72 | 5.66 (4.15, 7.70) | 57.80 (42.62, 70.43) | 8.399 (8.195, 8.604) |
| Austria | 66.55 (0.00, 175.46) | 1450.35 (597.33, 2653.10) | 2079.31 | 8.71 (0.00, 22.97) | 117.90 (48.56, 215.67) | 9.535 (9.001, 10.072) |
| Azerbaijan | 0.10 (0.07, 0.26) | 5.34 (3.64, 7.13) | 5360.85 | 0.04 (0.03, 0.12) | 1.63 (1.11, 2.18) | 13.552 (13.031, 14.075) |
| Bahamas | 16.03 (9.57, 26.50) | 68.94 (48.53, 86.23) | 330.05 | 199.40 (119.07, 329.57) | 368.25 (259.23, 460.59) | 2.086 (1.917, 2.256) |
| Bahrain | 0.19 (0.13, 0.30) | 1.85 (1.47, 2.15) | 891.09 | 3.18 (2.30, 5.07) | 7.93 (6.29, 9.21) | 3.409 (2.939, 3.881) |
| Bangladesh | 0.00 (0.00, 0.00) | 185.26 (79.63, 826.19) | - | 0.05 (0.05, 0.04) | 2.66 (1.14, 11.86) | 15.218 (13.914, 16.538) |
| Barbados | 26.28 (15.96, 45.26) | 89.90 (60.40, 119.15) | 242.06 | 122.97 (74.69, 211.76) | 295.45 (198.49, 391.59) | 3.080 (2.742, 3.419) |
| Belarus | 0.29 (0.00, 0.93) | 34.09 (23.13, 46.55) | 11653.46 | 0.04 (0.00, 0.13) | 3.66 (2.48, 5.00) | 16.942 (15.203, 18.707) |
| Belgium | 79.19 (44.78, 135.59) | 3283.87 (1375.56, 5762.35) | 4046.82 | 8.23 (4.65, 14.10) | 210.86 (88.33, 370.00) | 11.911 (11.785, 12.037) |
| Belize | 9.34 (6.33, 15.97) | 31.08 (17.24, 48.95) | 232.63 | 179.06 (121.27, 306.06) | 241.11 (133.75, 379.80) | 1.045 (0.819, 1.272) |
| Benin | 20.68 (12.66, 33.12) | 1058.29 (720.53, 1546.50) | 5017.03 | 20.09 (12.30, 32.17) | 485.86 (330.80, 710.01) | 11.561 (10.814, 12.313) |
| Bermuda | 6.51 (3.99, 11.80) | 8.24 (5.40, 10.33) | 26.63 | 186.28 (114.35, 337.91) | 97.33 (63.81, 122.04) | -2.196 (-2.647, -1.743) |
| Bhutan | 4.09 (0.22, 16.59) | 13.47 (0.46, 66.77) | 228.84 | 44.66 (2.45, 180.92) | 46.89 (1.61, 232.49) | 0.117 (-0.217, 0.452) |
| Bolivia (Plurinational State of) | 111.36 (15.42, 453.20) | 313.21 (59.28, 1241.24) | 181.26 | 73.72 (10.21, 300.03) | 68.92 (13.04, 273.13) | -0.210 (-0.380, -0.041) |
| Bosnia and Herzegovina | 0.03 (0.03, 0.04) | 9.39 (4.12, 15.65) | 27384.27 | 0.02 (0.02, 0.03) | 2.64 (1.16, 4.41) | 16.696 (14.836, 18.585) |
| Botswana | 346.66 (228.81, 488.08) | 6533.96 (4863.07, 8353.61) | 1784.85 | 1398.75 (923.25, 1969.37) | 12287.25 (9145.10, 15709.13) | 7.734 (7.429, 8.040) |
| Brazil | 285.72 (189.34, 471.08) | 28228.96 (16992.32, 40855.08) | 9780.00 | 6.75 (4.48, 11.14) | 215.68 (129.83, 312.14) | 12.744 (12.286, 13.204) |
| Brunei Darussalam | 0.31 (0.20, 0.46) | 8.08 (5.25, 11.76) | 2465.19 | 8.48 (5.45, 12.50) | 81.38 (52.91, 118.52) | 8.168 (7.638, 8.700) |
| Bulgaria | 0.27 (0.00, 0.67) | 33.02 (25.07, 40.93) | 12005.51 | 0.04 (0.00, 0.10) | 3.21 (2.44, 3.98) | 16.198 (15.666, 16.732) |
| Burkina Faso | 2804.41 (1701.00, 4609.69) | 1632.62 (1072.57, 2464.33) | -41.78 | 1425.96 (864.91, 2343.88) | 417.50 (274.28, 630.19) | -4.179 (-4.349, -4.008) |
| Burundi | 1676.63 (333.51, 4619.26) | 1176.17 (705.04, 1946.39) | -29.85 | 1460.89 (290.60, 4024.86) | 718.94 (430.96, 1189.74) | -2.527 (-2.897, -2.157) |
| Cabo Verde | 14.54 (4.99, 31.83) | 53.67 (32.50, 90.03) | 269.11 | 95.76 (32.89, 209.62) | 256.17 (155.14, 429.75) | 3.380 (3.103, 3.656) |
| Cambodia | 13.71 (7.28, 23.92) | 2195.76 (1473.86, 3034.39) | 15913.99 | 7.55 (4.01, 13.17) | 411.49 (276.20, 568.65) | 14.686 (14.016, 15.361) |
| Cameroon | 277.42 (167.34, 429.02) | 6449.32 (4356.40, 9244.03) | 2224.74 | 150.41 (90.73, 232.61) | 1318.42 (890.57, 1889.73) | 7.674 (7.073, 8.279) |
| Canada | 156.51 (95.65, 240.28) | 6362.62 (4306.63, 8598.26) | 3965.42 | 7.78 (4.76, 11.95) | 141.51 (95.78, 191.23) | 10.555 (10.389, 10.723) |
| Central African Republic | 486.56 (247.19, 803.35) | 1040.67 (565.33, 1755.49) | 113.88 | 1225.86 (622.79, 2024.01) | 1545.22 (839.42, 2606.60) | 0.602 (0.253, 0.952) |
| Chad | 253.21 (110.29, 554.36) | 1803.32 (1261.37, 2558.56) | 612.19 | 167.89 (73.12, 367.56) | 740.59 (518.02, 1050.75) | 5.203 (4.749, 5.659) |
| Chile | 36.39 (30.65, 42.57) | 1375.62 (1127.87, 1618.17) | 3679.71 | 6.82 (5.74, 7.98) | 95.40 (78.22, 112.22) | 9.495 (9.229, 9.762) |
| China | 4250.41 (2715.44, 10004.45) | 60519.62 (29198.77, 108120.78) | 1323.85 | 11.11 (7.10, 26.15) | 56.05 (27.04, 100.14) | 5.705 (5.292, 6.119) |
| Colombia | 68.16 (45.23, 116.63) | 2843.85 (1953.35, 3624.64) | 4072.18 | 8.21 (5.45, 14.05) | 91.94 (63.15, 117.19) | 8.694 (8.349, 9.039) |
| Comoros | 5.52 (4.37, 6.02) | 4.94 (2.62, 18.35) | -10.52 | 47.59 (37.68, 51.85) | 19.49 (10.35, 72.35) | -3.297 (-4.722, -1.850) |
| Congo | 365.86 (192.01, 644.42) | 1034.48 (599.85, 1748.68) | 182.75 | 827.62 (434.35, 1457.75) | 1038.40 (602.12, 1755.30) | 0.704 (0.382, 1.026) |
| Cook Islands | 0.01 (0.00, 0.06) | 0.59 (0.04, 2.62) | 4390.58 | 2.14 (0.42, 10.45) | 42.20 (2.56, 188.51) | 10.846 (10.692, 11.001) |
| Costa Rica | 8.58 (5.83, 12.19) | 247.42 (178.01, 305.67) | 2784.34 | 9.03 (6.14, 12.83) | 85.95 (61.84, 106.19) | 8.089 (8.004, 8.173) |
| Croatia | 1.69 (1.15, 2.98) | 79.88 (45.54, 117.65) | 4613.91 | 0.52 (0.35, 0.91) | 13.53 (7.71, 19.93) | 11.942 (11.666, 12.218) |
| Cuba | 7.55 (5.58, 11.71) | 966.78 (606.58, 1708.83) | 12709.87 | 1.20 (0.88, 1.86) | 79.79 (50.06, 141.02) | 15.525 (15.041, 16.010) |
| Cyprus | 0.27 (0.21, 0.35) | 14.54 (10.78, 18.91) | 5279.15 | 0.52 (0.41, 0.68) | 11.74 (8.71, 15.27) | 11.385 (10.999, 11.773) |
| Czechia | 3.24 (2.45, 4.60) | 159.04 (97.97, 237.08) | 4803.15 | 0.41 (0.31, 0.58) | 10.84 (6.68, 16.16) | 11.993 (11.412, 12.576) |
| Cote d'Ivoire | 1408.66 (300.17, 4065.41) | 5375.59 (3673.45, 7757.78) | 281.61 | 1054.44 (224.69, 3043.12) | 1354.09 (925.33, 1954.16) | 0.710 (0.350, 1.071) |
| Democratic People's Republic of Korea | 63.03 (16.96, 289.83) | 847.95 (127.68, 6189.97) | 1245.23 | 9.91 (2.67, 45.59) | 46.73 (7.04, 341.14) | 5.349 (4.760, 5.942) |
| Democratic Republic of the Congo | 2898.57 (1784.49, 4588.76) | 4453.27 (2968.94, 6612.99) | 53.64 | 494.44 (304.40, 782.75) | 331.39 (220.93, 492.10) | -1.397 (-1.618, -1.175) |
| Denmark | 61.87 (38.10, 94.60) | 715.53 (531.35, 964.06) | 1056.46 | 11.05 (6.81, 16.90) | 87.19 (64.75, 117.47) | 7.404 (7.177, 7.630) |
| Djibouti | 0.27 (0.11, 0.70) | 116.72 (59.63, 225.84) | 42989.59 | 6.26 (2.51, 16.07) | 558.69 (285.44, 1081.02) | 16.554 (15.749, 17.365) |
| Dominica | 4.21 (2.76, 7.35) | 5.88 (2.58, 9.70) | 39.76 | 83.18 (54.49, 145.29) | 108.30 (47.54, 178.52) | 0.912 (0.571, 1.255) |
| Dominican Republic | 145.28 (70.80, 267.04) | 1498.34 (951.48, 2247.09) | 931.37 | 78.11 (38.07, 143.58) | 296.79 (188.47, 445.10) | 4.570 (4.207, 4.933) |
| Ecuador | 25.12 (21.08, 31.06) | 625.85 (472.29, 799.02) | 2391.72 | 9.23 (7.75, 11.42) | 74.38 (56.13, 94.97) | 7.481 (7.163, 7.801) |
| Egypt | 11.27 (8.99, 15.58) | 101.02 (59.89, 151.03) | 796.74 | 0.89 (0.71, 1.23) | 3.94 (2.34, 5.89) | 5.212 (4.852, 5.574) |
| El Salvador | 36.08 (23.14, 60.11) | 1666.01 (740.36, 2734.80) | 4517.04 | 22.88 (14.68, 38.12) | 452.87 (201.25, 743.40) | 10.857 (10.651, 11.063) |
| Equatorial Guinea | 6.37 (3.00, 14.17) | 622.75 (383.86, 955.42) | 9672.13 | 80.27 (37.77, 178.48) | 3197.38 (1970.88, 4905.40) | 13.509 (13.155, 13.865) |
| Eritrea | 66.41 (19.77, 179.82) | 249.96 (160.68, 409.68) | 276.38 | 227.35 (67.66, 615.59) | 284.45 (182.85, 466.21) | 0.710 (0.254, 1.167) |
| Estonia | 0.06 (0.05, 0.08) | 8.16 (5.61, 11.96) | 14319.42 | 0.05 (0.04, 0.07) | 4.43 (3.05, 6.49) | 16.990 (16.654, 17.326) |
| Eswatini | 12.86 (8.25, 24.85) | 3702.85 (2408.64, 5038.28) | 28702.88 | 102.73 (65.94, 198.56) | 14611.18 (9504.32, 19880.70) | 18.652 (17.938, 19.370) |
| Ethiopia | 2920.59 (1495.10, 5325.37) | 12464.75 (9372.84, 16893.83) | 326.79 | 379.80 (194.43, 692.53) | 659.86 (496.18, 894.32) | 1.753 (1.165, 2.344) |
| Fiji | 1.43 (0.73, 3.75) | 9.78 (5.06, 17.68) | 581.63 | 10.55 (5.36, 27.59) | 32.05 (16.59, 57.96) | 3.989 (3.201, 4.783) |
| Finland | 3.06 (2.17, 4.38) | 164.44 (115.74, 236.69) | 5282.64 | 0.67 (0.47, 0.96) | 18.61 (13.10, 26.79) | 12.202 (11.661, 12.745) |
| France | 265.65 (163.95, 432.07) | 7635.73 (5920.31, 9291.04) | 2774.36 | 5.09 (3.14, 8.28) | 80.88 (62.71, 98.42) | 10.104 (9.943, 10.265) |
| Gabon | 51.36 (16.24, 151.62) | 841.60 (583.00, 1146.32) | 1538.52 | 192.06 (60.71, 566.95) | 1959.90 (1357.69, 2669.52) | 8.319 (7.846, 8.795) |
| Gambia | 6.88 (3.76, 12.14) | 332.08 (211.50, 491.42) | 4725.78 | 43.99 (24.06, 77.61) | 712.68 (453.91, 1054.65) | 10.067 (9.510, 10.627) |
| Georgia | 0.38 (0.28, 0.65) | 37.83 (20.00, 56.44) | 9952.54 | 0.12 (0.09, 0.20) | 10.12 (5.35, 15.10) | 16.616 (16.078, 17.157) |
| Germany | 415.33 (268.31, 611.33) | 7982.55 (6217.19, 10113.29) | 1821.96 | 5.14 (3.32, 7.57) | 60.20 (46.89, 76.27) | 8.890 (8.642, 9.139) |
| Ghana | 500.64 (330.54, 724.54) | 4120.66 (2907.52, 5859.47) | 723.08 | 196.65 (129.84, 284.60) | 604.45 (426.50, 859.51) | 3.914 (3.723, 4.106) |
| Greece | 10.81 (8.02, 14.42) | 213.60 (155.35, 320.22) | 1876.56 | 1.14 (0.84, 1.52) | 12.44 (9.05, 18.65) | 8.707 (8.345, 9.069) |
| Greenland | 1.02 (0.66, 1.53) | 8.86 (4.77, 14.78) | 767.33 | 79.47 (51.37, 119.29) | 282.89 (152.27, 471.84) | 4.491 (4.246, 4.737) |
| Grenada | 4.48 (2.83, 8.26) | 6.48 (3.07, 10.11) | 44.75 | 88.76 (56.16, 163.80) | 109.51 (51.85, 170.89) | 0.771 (0.495, 1.049) |
| Guam | 0.64 (0.20, 2.17) | 2.18 (0.91, 5.14) | 238.03 | 23.24 (7.23, 78.38) | 21.13 (8.85, 49.86) | -0.265 (-0.513, -0.017) |
| Guatemala | 47.03 (35.05, 77.39) | 967.51 (750.43, 1297.67) | 1957.02 | 30.72 (22.90, 50.55) | 158.54 (122.96, 212.64) | 5.812 (5.643, 5.981) |
| Guinea | 101.80 (64.52, 165.72) | 1918.60 (1378.00, 2704.19) | 1784.69 | 61.61 (39.05, 100.29) | 738.35 (530.30, 1040.67) | 8.887 (8.653, 9.123) |
| Guinea-Bissau | 18.49 (11.29, 29.60) | 328.80 (190.38, 525.12) | 1678.21 | 106.06 (64.75, 169.80) | 1174.58 (680.10, 1875.86) | 8.614 (8.338, 8.891) |
| Guyana | 35.24 (23.56, 60.22) | 331.05 (137.72, 548.40) | 839.53 | 201.28 (134.57, 343.98) | 1213.28 (504.72, 2009.85) | 6.566 (5.866, 7.270) |
| Haiti | 715.97 (285.25, 1412.03) | 2325.10 (1521.08, 3368.58) | 224.75 | 522.79 (208.28, 1031.04) | 795.81 (520.62, 1152.96) | 1.419 (1.238, 1.599) |
| Honduras | 0.02 (0.02, 0.03) | 344.29 (133.06, 613.53) | 1568880.40 | 0.02 (0.02, 0.03) | 111.70 (43.17, 199.05) | 32.225 (29.914, 34.576) |
| Hungary | 0.69 (0.56, 0.87) | 82.32 (57.47, 100.69) | 11808.01 | 0.08 (0.07, 0.10) | 6.31 (4.40, 7.72) | 16.177 (15.607, 16.751) |
| Iceland | 1.14 (0.60, 2.09) | 7.00 (5.50, 9.00) | 515.91 | 6.21 (3.27, 11.39) | 19.70 (15.47, 25.34) | 4.068 (3.927, 4.209) |
| India | 671.73 (413.67, 1514.79) | 15975.68 (11394.47, 21563.02) | 2278.30 | 4.11 (2.53, 9.26) | 28.27 (20.16, 38.15) | 6.982 (6.629, 7.336) |
| Indonesia | 104.07 (0.00, 425.62) | 2400.37 (2077.16, 2809.71) | 2206.47 | 2.73 (0.00, 11.17) | 26.72 (23.13, 31.28) | 2.957 (0.676, 5.290) |
| Iran (Islamic Republic of) | 45.72 (36.41, 67.57) | 426.46 (262.88, 704.88) | 832.74 | 4.76 (3.79, 7.03) | 12.28 (7.57, 20.30) | 3.388 (3.086, 3.691) |
| Iraq | 0.40 (0.10, 0.82) | 13.63 (7.14, 39.31) | 3284.11 | 0.11 (0.03, 0.21) | 1.40 (0.74, 4.05) | 9.282 (9.029, 9.536) |
| Ireland | 0.33 (0.24, 0.44) | 57.32 (43.64, 77.61) | 17299.83 | 0.12 (0.09, 0.17) | 11.89 (9.05, 16.09) | 16.897 (16.139, 17.660) |
| Israel | 57.69 (31.33, 103.91) | 1301.68 (604.27, 2354.16) | 2156.47 | 18.94 (10.29, 34.11) | 173.76 (80.66, 314.26) | 7.910 (7.157, 8.667) |
| Italy | 225.98 (141.38, 352.98) | 3666.06 (2770.24, 4917.26) | 1522.27 | 4.08 (2.55, 6.37) | 35.62 (26.92, 47.78) | 7.761 (7.621, 7.901) |
| Jamaica | 34.33 (20.47, 62.66) | 618.88 (450.55, 755.44) | 1702.98 | 29.51 (17.59, 53.87) | 350.43 (255.12, 427.75) | 8.931 (8.792, 9.071) |
| Japan | 46.47 (29.62, 71.07) | 9725.94 (4876.90, 15462.50) | 20831.16 | 0.47 (0.30, 0.72) | 35.28 (17.69, 56.09) | 16.059 (15.687, 16.432) |
| Jordan | 0.01 (0.01, 0.02) | 14.09 (6.39, 22.24) | 133113.34 | 0.02 (0.01, 0.04) | 5.11 (2.32, 8.06) | 20.884 (20.630, 21.139) |
| Kazakhstan | 0.25 (0.17, 0.43) | 9.70 (6.34, 13.12) | 3777.80 | 0.04 (0.03, 0.07) | 1.26 (0.83, 1.71) | 12.406 (10.705, 14.133) |
| Kenya | 4980.40 (4048.03, 6098.15) | 25529.22 (19886.33, 34236.98) | 412.59 | 1346.63 (1094.53, 1648.85) | 2884.38 (2246.83, 3868.21) | 2.575 (2.212, 2.940) |
| Kiribati | 0.14 (0.09, 0.23) | 0.54 (0.20, 0.94) | 284.98 | 10.12 (6.77, 16.99) | 23.86 (8.77, 41.75) | 3.086 (2.932, 3.240) |
| Kuwait | 0.13 (0.08, 0.21) | 7.15 (4.74, 9.55) | 5592.53 | 0.62 (0.38, 1.04) | 7.64 (5.07, 10.21) | 9.206 (8.591, 9.825) |
| Kyrgyzstan | 2.04 (1.14, 4.16) | 34.47 (24.10, 48.12) | 1592.31 | 1.32 (0.74, 2.71) | 18.98 (13.27, 26.49) | 9.853 (9.214, 10.495) |
| Lao People's Democratic Republic | 0.22 (0.00, 1.72) | 229.49 (78.28, 1096.87) | 103272.01 | 0.25 (0.00, 1.92) | 127.45 (43.47, 609.14) | 17.416 (15.201, 19.673) |
| Latvia | 1.69 (1.09, 2.96) | 17.71 (12.74, 22.08) | 949.72 | 0.81 (0.52, 1.42) | 6.29 (4.53, 7.85) | 7.295 (6.861, 7.732) |
| Lebanon | 19.06 (2.07, 116.60) | 51.25 (1.98, 216.22) | 168.92 | 18.82 (2.05, 115.12) | 15.99 (0.62, 67.47) | -0.569 (-0.800, -0.337) |
| Lesotho | 256.94 (173.28, 372.54) | 7286.59 (5552.03, 9441.32) | 2735.86 | 550.12 (371.00, 797.62) | 13378.33 (10193.65, 17334.45) | 11.604 (11.048, 12.163) |
| Liberia | 44.82 (9.79, 128.29) | 384.63 (252.37, 583.76) | 758.21 | 76.74 (16.76, 219.67) | 456.12 (299.27, 692.26) | 6.203 (5.368, 7.045) |
| Libya | 3.03 (1.45, 10.75) | 16.93 (2.27, 69.99) | 458.53 | 3.54 (1.70, 12.55) | 7.28 (0.98, 30.10) | 2.448 (2.247, 2.651) |
| Lithuania | 0.05 (0.00, 0.10) | 15.21 (9.93, 20.83) | 28703.28 | 0.02 (0.00, 0.04) | 3.80 (2.48, 5.21) | 20.218 (18.861, 21.590) |
| Luxembourg | 2.82 (1.55, 4.97) | 32.31 (20.88, 52.20) | 1044.52 | 8.25 (4.54, 14.52) | 51.32 (33.18, 82.93) | 6.499 (6.334, 6.665) |
| Madagascar | 0.60 (0.21, 1.16) | 287.89 (131.99, 538.75) | 47903.32 | 0.27 (0.09, 0.52) | 77.64 (35.59, 145.29) | 21.485 (20.507, 22.470) |
| Malawi | 3141.54 (1923.95, 4955.77) | 16896.32 (12096.48, 22184.49) | 437.84 | 1973.93 (1208.88, 3113.86) | 5265.38 (3769.61, 6913.33) | 3.411 (3.082, 3.740) |
| Malaysia | 40.21 (22.95, 84.80) | 533.42 (337.71, 783.90) | 1226.62 | 9.56 (5.46, 20.17) | 41.62 (26.35, 61.17) | 5.188 (4.802, 5.576) |
| Maldives | 0.02 (0.02, 0.03) | 0.36 (0.28, 0.43) | 1516.48 | 0.75 (0.64, 0.90) | 2.82 (2.16, 3.33) | 4.739 (4.252, 5.229) |
| Mali | 158.49 (50.45, 407.08) | 1581.62 (1049.22, 2299.28) | 897.90 | 89.73 (28.56, 230.47) | 421.38 (279.54, 612.58) | 5.360 (4.813, 5.910) |
| Malta | 0.89 (0.68, 1.12) | 24.68 (15.24, 33.32) | 2666.88 | 3.57 (2.73, 4.48) | 37.45 (23.12, 50.56) | 8.472 (8.294, 8.650) |
| Marshall Islands | 0.51 (0.02, 2.88) | 0.99 (0.03, 6.85) | 93.88 | 67.45 (3.28, 381.49) | 90.46 (2.54, 628.55) | 1.031 (0.740, 1.323) |
| Mauritania | 232.49 (136.79, 569.45) | 75.29 (29.95, 362.73) | -67.61 | 443.34 (260.85, 1085.89) | 74.79 (29.75, 360.29) | -6.019 (-6.307, -5.730) |
| Mauritius | 0.90 (0.67, 1.37) | 40.88 (28.54, 57.36) | 4439.29 | 2.66 (1.97, 4.05) | 45.16 (31.52, 63.36) | 10.320 (8.716, 11.947) |
| Mexico | 306.82 (153.43, 701.59) | 4458.77 (2910.15, 5784.97) | 1353.20 | 14.62 (7.31, 33.42) | 70.00 (45.69, 90.83) | 5.686 (5.290, 6.084) |
| Micronesia (Federated States of) | 1.02 (0.11, 5.98) | 6.48 (0.18, 51.37) | 534.62 | 49.89 (5.49, 292.29) | 285.71 (7.90, 2266.47) | 5.684 (2.579, 8.883) |
| Monaco | 0.79 (0.14, 3.20) | 2.91 (0.41, 11.00) | 267.92 | 15.87 (2.83, 64.25) | 41.69 (5.88, 157.70) | 3.436 (3.283, 3.589) |
| Mongolia | 0.00 (0.00, 0.00) | 7.71 (2.34, 16.96) | 2540838.88 | 0.02 (0.05, 0.00) | 9.22 (2.80, 20.28) | 24.056 (22.587, 25.543) |
| Montenegro | 0.06 (0.04, 0.10) | 3.12 (1.66, 4.69) | 5194.14 | 0.18 (0.13, 0.31) | 5.38 (2.86, 8.09) | 12.308 (11.988, 12.629) |
| Morocco | 50.23 (18.11, 211.24) | 320.59 (25.04, 1071.01) | 538.22 | 8.06 (2.91, 33.91) | 23.16 (1.81, 77.37) | 3.663 (3.496, 3.831) |
| Mozambique | 564.25 (335.87, 920.73) | 24619.76 (17983.87, 32506.71) | 4263.29 | 230.19 (137.03, 375.63) | 5490.45 (4010.58, 7249.32) | 11.520 (11.230, 11.811) |
| Myanmar | 91.09 (60.21, 122.80) | 4684.88 (3414.97, 6125.95) | 5043.09 | 9.19 (6.07, 12.38) | 222.53 (162.21, 290.99) | 11.335 (10.796, 11.876) |
| Namibia | 132.34 (97.10, 183.30) | 5010.35 (3786.40, 6310.26) | 3685.85 | 351.85 (258.15, 487.32) | 7275.72 (5498.37, 9163.37) | 11.060 (10.422, 11.701) |
| Nauru | 0.00 (0.00, 0.01) | 0.04 (0.00, 0.17) | 1384.36 | 2.38 (0.73, 10.61) | 49.30 (2.82, 216.29) | 10.969 (10.556, 11.383) |
| Nepal | 0.20 (0.00, 1.27) | 805.76 (34.76, 3509.86) | 393906.69 | 0.06 (0.00, 0.35) | 73.63 (3.18, 320.74) | 26.004 (23.788, 28.259) |
| Netherlands | 59.53 (37.41, 92.43) | 2130.81 (1485.14, 2723.21) | 3479.61 | 4.63 (2.91, 7.19) | 91.48 (63.76, 116.91) | 10.862 (10.643, 11.081) |
| New Zealand | 12.01 (7.37, 19.62) | 119.57 (90.66, 150.12) | 895.17 | 4.86 (2.98, 7.93) | 23.06 (17.49, 28.96) | 5.538 (5.324, 5.752) |
| Nicaragua | 0.54 (0.43, 0.74) | 298.57 (111.47, 481.72) | 55555.73 | 0.73 (0.58, 1.00) | 138.73 (51.80, 223.84) | 19.909 (19.603, 20.215) |
| Niger | 68.09 (32.88, 135.97) | 527.62 (346.33, 820.04) | 674.84 | 65.83 (31.78, 131.44) | 170.05 (111.62, 264.29) | 3.279 (2.739, 3.821) |
| Nigeria | 2167.98 (1525.78, 3154.98) | 26559.38 (21918.65, 32376.90) | 1125.07 | 105.93 (74.55, 154.15) | 752.17 (620.75, 916.93) | 6.888 (6.537, 7.239) |
| Niue | 0.00 (0.00, 0.01) | 0.05 (0.00, 0.20) | 2037.99 | 1.51 (0.43, 6.71) | 37.39 (2.13, 160.94) | 11.701 (11.374, 12.030) |
| North Macedonia | 0.03 (0.02, 0.03) | 2.68 (1.44, 4.16) | 10598.89 | 0.03 (0.02, 0.03) | 1.50 (0.80, 2.33) | 14.712 (14.536, 14.887) |
| Northern Mariana Islands | 0.02 (0.01, 0.06) | 0.21 (0.09, 0.51) | 1086.12 | 3.65 (1.47, 11.56) | 13.33 (5.72, 32.51) | 4.551 (4.252, 4.851) |
| Norway | 13.32 (7.74, 23.05) | 158.10 (122.81, 209.29) | 1086.78 | 2.73 (1.58, 4.72) | 24.25 (18.84, 32.10) | 7.841 (7.735, 7.948) |
| Oman | 0.32 (0.23, 0.50) | 22.44 (14.35, 34.53) | 6850.64 | 1.40 (1.02, 2.16) | 41.65 (26.63, 64.09) | 12.431 (11.724, 13.142) |
| Pakistan | 394.61 (109.31, 1624.77) | 750.99 (54.61, 3816.30) | 90.31 | 13.38 (3.71, 55.11) | 16.61 (1.21, 84.43) | 0.759 (0.302, 1.218) |
| Palau | 0.01 (0.00, 0.05) | 0.35 (0.02, 1.63) | 3212.57 | 2.22 (0.53, 10.49) | 43.55 (2.12, 201.70) | 10.807 (10.607, 11.007) |
| Palestine | 0.01 (0.01, 0.01) | 0.08 (0.05, 0.12) | 730.66 | 0.02 (0.02, 0.03) | 0.09 (0.05, 0.12) | 4.568 (4.361, 4.775) |
| Panama | 32.04 (21.45, 53.79) | 361.60 (244.37, 500.78) | 1028.69 | 39.23 (26.26, 65.86) | 147.71 (99.82, 204.56) | 4.625 (4.522, 4.728) |
| Papua New Guinea | 0.92 (0.28, 2.26) | 877.26 (24.60, 2444.25) | 95011.01 | 1.41 (0.43, 3.47) | 534.33 (14.98, 1488.76) | 22.684 (22.261, 23.108) |
| Paraguay | 3.98 (2.96, 6.97) | 321.09 (139.24, 527.57) | 7974.01 | 3.42 (2.55, 5.99) | 110.69 (48.00, 181.87) | 12.749 (12.387, 13.112) |
| Peru | 117.18 (91.78, 170.27) | 2643.02 (1182.45, 4199.12) | 2155.48 | 19.60 (15.35, 28.48) | 143.14 (64.04, 227.41) | 7.013 (6.760, 7.265) |
| Philippines | 0.00 (0.00, 0.00) | 1256.98 (719.58, 2147.63) | - | 0.95 (5.17, 0.10) | 37.34 (21.37, 63.79) | 13.308 (11.720, 14.919) |
| Poland | 1.02 (0.67, 1.61) | 425.61 (266.97, 645.02) | 41516.11 | 0.04 (0.03, 0.07) | 9.67 (6.07, 14.66) | 20.597 (20.072, 21.124) |
| Portugal | 170.27 (99.80, 285.36) | 1388.31 (1187.69, 1600.21) | 715.37 | 19.73 (11.57, 33.07) | 81.43 (69.66, 93.86) | 5.002 (4.731, 5.275) |
| Puerto Rico | 165.15 (94.74, 302.85) | 298.10 (163.09, 419.70) | 80.51 | 73.42 (42.12, 134.63) | 58.77 (32.15, 82.74) | -0.699 (-1.000, -0.397) |
| Qatar | 0.00 (0.00, 0.00) | 1.11 (0.78, 1.48) | 183646.63 | 0.03 (0.02, 0.04) | 7.30 (5.09, 9.75) | 21.737 (20.697, 22.786) |
| Republic of Korea | 40.54 (0.00, 115.38) | 4261.42 (2258.50, 7729.62) | 10412.26 | 3.19 (0.00, 9.08) | 81.13 (43.00, 147.15) | 11.968 (11.210, 12.731) |
| Republic of Moldova | 1.41 (0.74, 2.84) | 57.66 (43.19, 74.42) | 3978.78 | 0.67 (0.35, 1.35) | 17.92 (13.42, 23.13) | 11.767 (10.365, 13.187) |
| Romania | 22.75 (15.74, 35.31) | 544.83 (277.39, 886.70) | 2294.81 | 1.59 (1.10, 2.46) | 22.22 (11.31, 36.16) | 9.484 (9.199, 9.771) |
| Russian Federation | 32.23 (21.35, 51.67) | 1527.68 (891.69, 2306.63) | 4640.51 | 0.33 (0.22, 0.54) | 11.21 (6.54, 16.93) | 12.945 (12.402, 13.491) |
| Rwanda | 525.05 (169.78, 1369.16) | 3819.96 (2696.59, 5222.42) | 627.55 | 428.15 (138.45, 1116.48) | 1633.88 (1153.39, 2233.74) | 4.577 (4.296, 4.858) |
| Saint Kitts and Nevis | 1.34 (0.14, 4.28) | 9.96 (1.77, 31.86) | 641.98 | 50.22 (5.29, 159.98) | 372.82 (66.39, 1192.54) | 7.158 (6.993, 7.323) |
| Saint Lucia | 3.65 (2.44, 6.26) | 8.81 (4.13, 14.08) | 141.29 | 69.32 (46.31, 118.87) | 71.59 (33.54, 114.42) | 0.169 (-0.298, 0.638) |
| Saint Vincent and the Grenadines | 7.63 (4.72, 14.13) | 8.00 (5.45, 10.60) | 4.87 | 172.65 (106.88, 319.74) | 106.55 (72.59, 141.12) | -1.546 (-2.096, -0.993) |
| Samoa | 2.13 (0.19, 8.67) | 4.88 (0.25, 34.66) | 128.67 | 51.72 (4.68, 210.32) | 71.97 (3.65, 511.45) | 1.133 (0.803, 1.463) |
| San Marino | 0.41 (0.05, 1.72) | 1.82 (0.27, 6.76) | 344.59 | 19.02 (2.28, 79.94) | 41.00 (6.06, 152.19) | 2.691 (2.610, 2.771) |
| Sao Tome and Principe | 1.53 (1.24, 1.98) | 0.85 (0.60, 1.84) | -44.79 | 46.12 (37.40, 59.60) | 18.98 (13.49, 41.22) | -3.410 (-3.868, -2.951) |
| Saudi Arabia | 19.83 (10.00, 33.82) | 73.41 (31.29, 241.83) | 270.17 | 8.07 (4.07, 13.76) | 15.09 (6.43, 49.71) | 2.177 (1.917, 2.437) |
| Senegal | 75.07 (42.89, 123.90) | 839.86 (602.98, 1147.44) | 1018.80 | 49.64 (28.36, 81.92) | 242.66 (174.22, 331.53) | 5.530 (5.140, 5.921) |
| Serbia | 4.00 (0.00, 6.63) | 84.92 (29.56, 136.62) | 2021.68 | 0.76 (0.00, 1.26) | 8.26 (2.87, 13.28) | 8.482 (7.008, 9.978) |
| Seychelles | 0.27 (0.13, 0.64) | 2.25 (1.75, 2.82) | 721.67 | 7.96 (3.64, 18.54) | 42.82 (33.33, 53.77) | 5.983 (5.760, 6.206) |
| Sierra Leone | 44.71 (14.18, 123.84) | 924.81 (603.26, 1392.79) | 1968.47 | 43.75 (13.87, 121.17) | 559.55 (365.00, 842.69) | 9.132 (8.843, 9.422) |
| Singapore | 11.80 (6.13, 20.90) | 475.81 (311.19, 665.90) | 3932.25 | 11.27 (5.86, 19.97) | 113.85 (74.46, 159.33) | 8.293 (8.114, 8.473) |
| Slovakia | 0.10 (0.00, 0.35) | 9.83 (5.42, 15.13) | 10181.16 | 0.03 (0.00, 0.11) | 1.75 (0.96, 2.69) | 15.203 (14.583, 15.826) |
| Slovenia | 0.03 (0.00, 0.09) | 11.65 (5.60, 19.73) | 37906.54 | 0.02 (0.00, 0.06) | 4.06 (1.95, 6.87) | 19.814 (18.983, 20.651) |
| Solomon Islands | 2.86 (0.23, 15.24) | 9.22 (0.43, 68.00) | 221.93 | 67.38 (5.31, 358.63) | 89.59 (4.14, 661.06) | 1.021 (0.641, 1.403) |
| Somalia | 7.21 (2.49, 21.10) | 265.61 (104.35, 527.64) | 3581.98 | 8.88 (3.07, 25.96) | 116.74 (45.86, 231.91) | 9.049 (8.045, 10.062) |
| South Africa | 1072.62 (887.54, 1291.72) | 164017.23 (142670.43, 188541.90) | 15191.20 | 104.18 (86.21, 125.46) | 7593.32 (6605.05, 8728.71) | 15.961 (15.653, 16.269) |
| South Sudan | 75.00 (17.02, 264.43) | 1083.58 (415.75, 2371.87) | 1344.85 | 66.69 (15.13, 235.14) | 704.54 (270.32, 1542.17) | 8.373 (7.848, 8.901) |
| Spain | 248.45 (156.06, 376.22) | 2502.58 (2087.15, 3178.74) | 907.28 | 7.21 (4.53, 10.92) | 37.77 (31.50, 47.97) | 5.904 (5.467, 6.342) |
| Sri Lanka | 1.11 (0.60, 2.34) | 22.98 (16.46, 30.85) | 1978.49 | 0.22 (0.12, 0.47) | 1.69 (1.21, 2.26) | 7.435 (6.981, 7.890) |
| Sudan | 72.61 (11.79, 258.37) | 1070.60 (312.70, 3113.33) | 1374.49 | 15.43 (2.50, 54.90) | 127.68 (37.29, 371.31) | 7.429 (6.882, 7.979) |
| Suriname | 25.48 (14.57, 51.50) | 70.69 (45.12, 96.44) | 177.37 | 197.67 (113.01, 399.46) | 224.38 (143.22, 306.13) | 0.412 (-0.347, 1.177) |
| Sweden | 33.42 (20.15, 55.36) | 365.88 (186.79, 615.27) | 994.83 | 3.06 (1.84, 5.07) | 23.79 (12.15, 40.01) | 7.339 (7.235, 7.442) |
| Switzerland | 130.64 (81.19, 206.35) | 2098.95 (1223.75, 3016.31) | 1506.63 | 18.71 (11.63, 29.56) | 175.54 (102.34, 252.25) | 8.048 (7.839, 8.256) |
| Syrian Arab Republic | 0.16 (0.13, 0.24) | 3.28 (2.27, 4.92) | 1906.80 | 0.07 (0.06, 0.11) | 0.61 (0.42, 0.92) | 7.698 (7.004, 8.396) |
| Taiwan (Province of China) | 7.45 (4.80, 11.68) | 192.87 (98.33, 330.96) | 2490.61 | 1.02 (0.66, 1.60) | 8.61 (4.39, 14.78) | 7.475 (7.020, 7.931) |
| Tajikistan | 0.05 (0.03, 0.09) | 8.50 (6.14, 11.35) | 18671.17 | 0.03 (0.02, 0.07) | 5.92 (4.28, 7.91) | 19.809 (19.129, 20.492) |
| Thailand | 770.82 (321.72, 2031.21) | 24999.64 (12686.00, 40244.56) | 3143.25 | 50.41 (21.04, 132.85) | 441.94 (224.26, 711.44) | 7.883 (7.428, 8.340) |
| Timor-Leste | 17.73 (0.20, 158.80) | 109.55 (3.85, 737.76) | 517.85 | 205.14 (2.29, 1837.30) | 277.21 (9.74, 1866.84) | 0.964 (0.642, 1.286) |
| Togo | 69.49 (22.40, 186.19) | 1469.80 (1020.14, 2109.91) | 2015.10 | 135.00 (43.52, 361.72) | 1045.63 (725.74, 1501.02) | 7.174 (6.489, 7.864) |
| Tokelau | 0.00 (0.00, 0.01) | 0.03 (0.00, 0.12) | 1269.71 | 2.37 (0.77, 10.25) | 43.68 (2.53, 193.92) | 10.544 (10.271, 10.819) |
| Tonga | 0.27 (0.10, 0.86) | 1.44 (0.46, 3.66) | 442.26 | 10.12 (3.92, 32.72) | 32.55 (10.32, 82.92) | 4.177 (3.906, 4.449) |
| Trinidad and Tobago | 12.30 (7.73, 21.24) | 199.01 (127.75, 273.40) | 1518.61 | 26.05 (16.38, 45.01) | 190.65 (122.38, 261.91) | 7.110 (7.005, 7.216) |
| Tunisia | 3.07 (0.68, 13.32) | 60.84 (9.32, 209.06) | 1879.73 | 1.26 (0.28, 5.47) | 9.28 (1.42, 31.87) | 7.138 (6.814, 7.464) |
| Turkey | 9.29 (0.00, 20.55) | 186.67 (153.62, 227.65) | 1908.82 | 0.59 (0.00, 1.29) | 3.90 (3.21, 4.76) | 6.596 (5.517, 7.686) |
| Turkmenistan | 0.02 (0.01, 0.04) | 4.53 (3.34, 6.50) | 23625.05 | 0.02 (0.01, 0.04) | 3.00 (2.21, 4.30) | 18.362 (17.514, 19.216) |
| Tuvalu | 0.01 (0.00, 0.03) | 0.24 (0.01, 1.01) | 3732.48 | 2.12 (0.77, 8.82) | 47.04 (2.70, 202.60) | 11.287 (11.131, 11.443) |
| Uganda | 10731.74 (7158.12, 15159.01) | 18717.15 (13623.33, 24360.07) | 74.41 | 3627.92 (2419.84, 5124.59) | 3139.43 (2285.04, 4085.91) | -0.513 (-0.601, -0.425) |
| Ukraine | 3.28 (2.22, 5.00) | 349.19 (233.11, 467.62) | 10559.36 | 0.08 (0.06, 0.12) | 7.40 (4.94, 9.92) | 16.751 (15.750, 17.761) |
| United Arab Emirates | 0.22 (0.04, 1.31) | 301.41 (13.26, 2085.72) | 135195.50 | 2.36 (0.47, 13.84) | 747.56 (32.89, 5172.96) | 22.044 (20.406, 23.705) |
| United Kingdom | 180.86 (114.01, 290.58) | 8761.01 (4705.57, 12441.40) | 4744.12 | 2.91 (1.83, 4.68) | 99.80 (53.60, 141.72) | 13.101 (12.778, 13.424) |
| United Republic of Tanzania | 8489.18 (5017.67, 13298.42) | 24623.24 (16249.06, 35288.16) | 190.05 | 1725.19 (1019.70, 2702.54) | 2248.00 (1483.47, 3221.66) | 0.886 (0.760, 1.012) |
| United States of America | 5131.93 (3272.03, 7890.53) | 112026.86 (55881.60, 176696.37) | 2082.94 | 24.05 (15.33, 36.97) | 311.57 (155.42, 491.43) | 9.255 (9.146, 9.364) |
| United States Virgin Islands | 2.50 (1.44, 4.47) | 13.03 (9.39, 17.19) | 420.93 | 61.46 (35.49, 109.89) | 105.53 (76.10, 139.28) | 1.895 (1.763, 2.026) |
| Uruguay | 431.50 (252.84, 726.20) | 625.31 (516.77, 753.96) | 44.91 | 175.70 (102.95, 295.69) | 169.44 (140.03, 204.30) | -0.094 (-0.312, 0.124) |
| Uzbekistan | 6.31 (2.94, 19.62) | 37.62 (17.89, 69.54) | 496.44 | 1.17 (0.54, 3.63) | 6.87 (3.27, 12.70) | 6.761 (6.010, 7.518) |
| Vanuatu | 1.61 (0.18, 8.66) | 9.26 (0.59, 64.04) | 474.57 | 57.85 (6.41, 310.86) | 116.47 (7.46, 805.59) | 2.079 (-0.245, 4.457) |
| Venezuela (Bolivarian Republic of) | 210.86 (101.98, 471.70) | 2465.18 (1660.26, 3320.81) | 1069.08 | 44.36 (21.45, 99.24) | 161.20 (108.57, 217.15) | 4.660 (4.199, 5.123) |
| Viet Nam | 238.85 (137.34, 497.34) | 794.70 (329.48, 1602.70) | 232.73 | 11.72 (6.74, 24.41) | 19.28 (7.99, 38.87) | 1.735 (1.197, 2.275) |
| Yemen | 17.45 (4.94, 90.98) | 82.45 (6.47, 369.20) | 372.57 | 8.98 (2.54, 46.80) | 14.58 (1.15, 65.30) | 1.657 (1.474, 1.840) |
| Zambia | 2432.21 (1460.38, 3760.56) | 17522.97 (12058.66, 23978.92) | 620.45 | 2092.85 (1256.61, 3235.86) | 6371.44 (4384.59, 8718.86) | 3.905 (3.697, 4.114) |
| Zimbabwe | 2977.38 (812.08, 7257.24) | 20577.96 (14164.99, 29302.20) | 591.14 | 1659.15 (452.53, 4044.12) | 7109.49 (4893.87, 10123.63) | 5.045 (4.680, 5.411) |

Note: ASR: age-standardized rates; AAPC, average annual percentage change; CI, confidence interval; UI, uncertainty interval


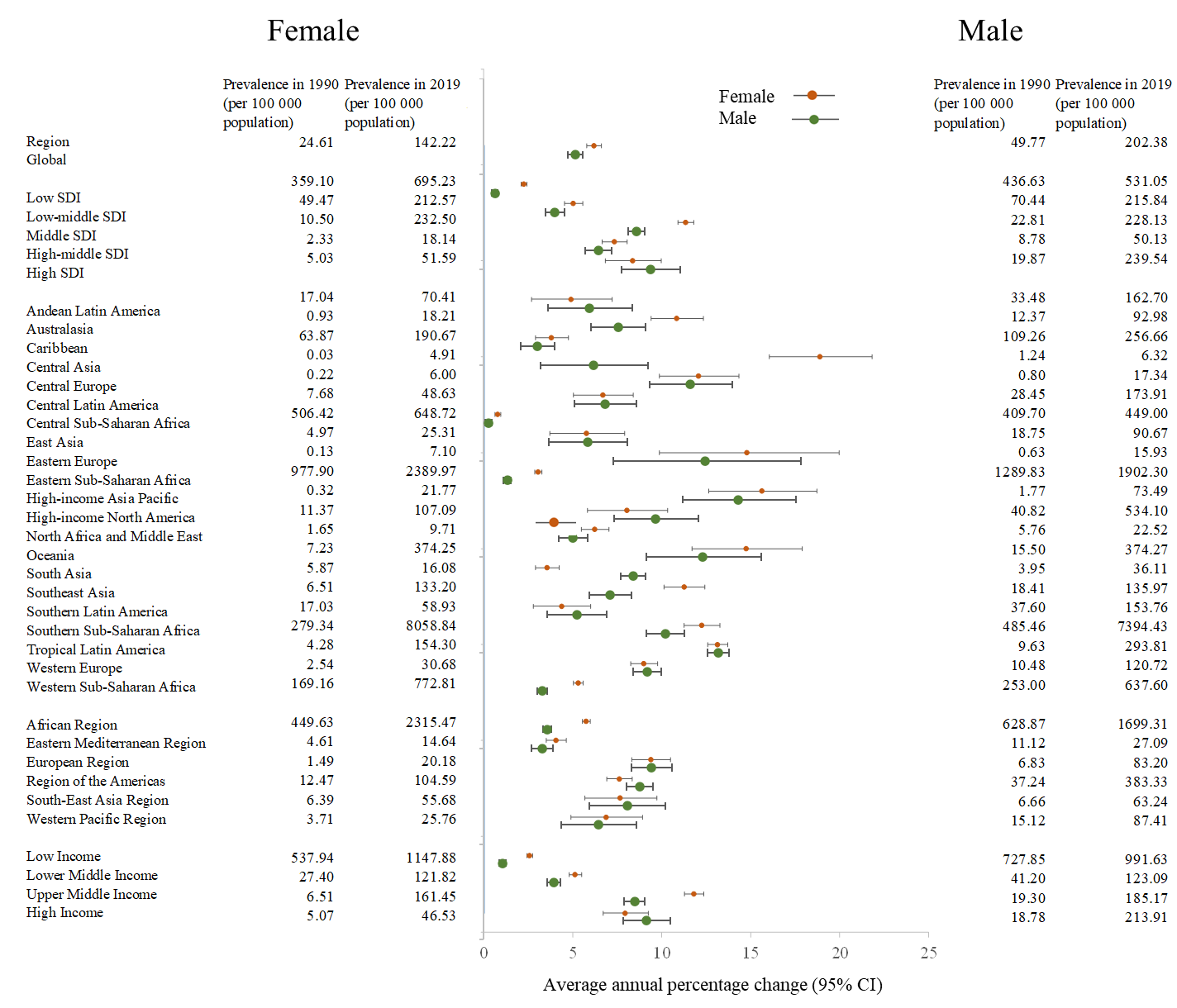


**Supplementary Figure S3**. The prevalence and its trend of HIV/AIDs from 1990 to 2019 by global, SDI, GBD, WHO and World Bank income regions.

Note: AAPC: average annual percentage changes; CI: confidence interval; GBD: Global Burden of Diseases, Injuries, and Risk Factors Study; SDI: Sociodemographic Index; WHO: World Health Organization

**Supplementary Table S7**. The HIV/AIDs prevalence and its trends among older adults by region and sex level from 1990 to 2019.

|  | Female |  |  | Male |  |  |
| --- | --- | --- | --- | --- | --- | --- |
|  | 1990 (95% UI) | 2019 (95% UI) | AAPC (95% CI) | 1990 (95% UI) | 2019 (95% UI) | AAPC (95% CI) |
| **Global** | 24.61 (20.81, 29.05) | 142.22 (125.45, 161.98) | 6.203 (6.058, 6.349) | 49.77 (40.99, 61.27) | 202.38 (167.90, 238.69) | 4.938 (4.669, 5.207) |
| **SDI region** |  |  |  |  |  |  |
| Low SDI | 359.10 (289.55, 442.87) | 695.23 (572.54, 849.22) | 2.273 (2.105, 2.442) | 436.63 (323.31, 590.99) | 531.05 (428.36, 658.55) | 0.615 (0.404, 0.826) |
| Low-middle SDI | 49.47 (38.41, 67.81) | 212.57 (179.87, 256.73) | 5.048 (4.668, 5.429) | 70.44 (52.67, 102.97) | 215.84 (182.55, 259.93) | 3.844 (3.562, 4.126) |
| Middle SDI | 10.50 (7.78, 17.13) | 232.50 (201.47, 269.07) | 11.368 (11.133, 11.604) | 22.81 (15.67, 39.37) | 228.13 (195.57, 269.10) | 8.239 (7.768, 8.712) |
| High-middle SDI | 2.33 (1.66, 3.49) | 18.14 (15.56, 20.95) | 7.360 (7.166, 7.555) | 8.78 (6.40, 12.87) | 50.13 (44.41, 56.81) | 6.191 (6.014, 6.368) |
| High SDI | 5.03 (3.23, 7.78) | 51.59 (29.16, 74.27) | 8.387 (8.204, 8.570) | 19.87 (13.21, 29.77) | 239.54 (136.38, 356.45) | 8.992 (8.870, 9.113) |
| **GBD region** |  |  |  |  |  |  |
| Andean Latin America | 17.04 (8.20, 40.78) | 70.41 (35.35, 113.80) | 4.927 (4.220, 5.639) | 33.48 (18.99, 76.77) | 162.70 (85.19, 249.38) | 5.697 (5.211, 6.186) |
| Australasia | 0.93 (0.61, 1.37) | 18.21 (12.26, 25.33) | 10.869 (10.589, 11.149) | 12.37 (9.04, 17.05) | 92.98 (70.70, 113.11) | 7.243 (6.995, 7.491) |
| Caribbean | 63.87 (37.55, 106.85) | 190.67 (148.05, 244.32) | 3.825 (3.538, 4.113) | 109.26 (65.38, 179.66) | 256.66 (189.54, 334.42) | 2.920 (2.668, 3.172) |
| Central Asia | 0.03 (0.02, 0.06) | 4.91 (3.16, 7.02) | 18.902 (18.042, 19.768) | 1.24 (0.65, 3.17) | 6.32 (4.25, 8.35) | 5.942 (5.444, 6.442) |
| Central Europe | 0.22 (0.15, 0.34) | 6.00 (3.84, 8.51) | 12.082 (11.563, 12.603) | 0.80 (0.54, 1.21) | 17.34 (10.58, 25.33) | 11.142 (10.772, 11.513) |
| Central Latin America | 7.68 (4.06, 16.87) | 48.63 (34.27, 61.84) | 6.713 (6.381, 7.047) | 28.45 (15.98, 57.12) | 173.91 (111.25, 228.57) | 6.560 (6.278, 6.843) |
| Central Sub-Saharan Africa | 506.42 (332.07, 754.62) | 648.72 (480.31, 871.86) | 0.804 (0.605, 1.004) | 409.70 (253.03, 659.24) | 449.00 (337.91, 595.12) | 0.281 (0.042, 0.520) |
| East Asia | 4.97 (3.00, 11.86) | 25.31 (11.87, 48.05) | 5.797 (5.429, 6.165) | 18.75 (12.06, 42.91) | 90.67 (44.08, 159.89) | 5.603 (5.231, 5.977) |
| Eastern Europe | 0.13 (0.09, 0.21) | 7.10 (4.56, 10.66) | 14.808 (14.442, 15.175) | 0.63 (0.41, 1.00) | 15.93 (9.29, 23.97) | 11.918 (11.127, 12.715) |
| Eastern Sub-Saharan Africa | 977.90 (781.10, 1212.42) | 2389.97 (1985.80, 2914.17) | 3.073 (2.879, 3.269) | 1289.83 (946.09, 1717.44) | 1902.30 (1532.35, 2379.83) | 1.277 (1.022, 1.533) |
| High-income Asia Pacific | 0.32 (0.19, 0.52) | 21.77 (11.17, 32.53) | 15.641 (15.416, 15.867) | 1.77 (0.77, 3.29) | 73.49 (39.43, 111.58) | 13.701 (13.356, 14.047) |
| High-income North America | 11.37 (7.11, 17.95) | 107.09 (55.00, 162.59) | 8.062 (7.908, 8.217) | 40.82 (26.15, 62.25) | 534.10 (274.30, 818.76) | 9.270 (9.075, 9.466) |
| North Africa and Middle East | 1.65 (0.75, 4.49) | 9.71 (5.25, 18.21) | 6.257 (6.035, 6.479) | 5.76 (2.62, 17.36) | 22.52 (9.27, 60.37) | 4.830 (4.699, 4.962) |
| Oceania | 7.23 (1.86, 34.07) | 374.25 (19.11, 956.14) | 14.764 (14.131, 15.400) | 15.50 (3.71, 51.59) | 374.27 (18.41, 1170.66) | 11.808 (10.825, 12.799) |
| South Asia | 5.87 (2.97, 13.91) | 16.08 (11.22, 24.09) | 3.569 (3.346, 3.792) | 3.95 (1.89, 13.08) | 36.11 (22.41, 57.75) | 8.058 (7.868, 8.248) |
| Southeast Asia | 6.51 (2.94, 16.63) | 133.20 (80.47, 211.24) | 11.278 (10.646, 11.913) | 18.41 (8.69, 43.62) | 135.97 (92.96, 188.31) | 6.819 (6.214, 7.428) |
| Southern Latin America | 17.03 (10.60, 28.72) | 58.93 (46.96, 73.25) | 4.392 (4.215, 4.569) | 37.60 (25.68, 54.28) | 153.76 (133.18, 176.05) | 5.019 (4.874, 5.165) |
| Southern Sub-Saharan Africa | 279.34 (154.80, 527.11) | 8058.84 (6882.06, 9363.89) | 12.263 (11.875, 12.654) | 485.46 (245.27, 1011.35) | 7394.43 (6107.88, 8909.09) | 9.788 (9.349, 10.229) |
| Tropical Latin America | 4.28 (2.81, 7.06) | 154.30 (99.15, 214.89) | 13.154 (12.565, 13.747) | 9.63 (6.24, 15.95) | 293.81 (166.84, 444.55) | 12.620 (12.279, 12.963) |
| Western Europe | 2.54 (1.63, 3.94) | 30.68 (20.78, 38.99) | 9.010 (8.784, 9.236) | 10.48 (6.82, 16.24) | 120.72 (90.36, 146.29) | 8.814 (8.528, 9.101) |
| Western Sub-Saharan Africa | 169.16 (123.56, 242.42) | 772.81 (632.21, 954.77) | 5.317 (5.068, 5.566) | 253.00 (174.15, 388.81) | 637.60 (514.56, 807.86) | 3.182 (2.861, 3.505) |
| **WHO region** |  |  |  |  |  |  |
| African Region | 449.63 (369.66, 535.29) | 2315.47 (1996.36, 2691.28) | 5.766 (5.562, 5.969) | 628.87 (487.03, 800.83) | 1699.31 (1478.59, 1981.08) | 3.438 (3.226, 3.650) |
| Eastern Mediterranean Region | 4.61 (1.68, 9.52) | 14.64 (7.60, 32.01) | 4.073 (3.881, 4.264) | 11.12 (4.21, 34.91) | 27.09 (10.00, 78.95) | 3.165 (2.988, 3.343) |
| European Region | 1.49 (0.98, 2.27) | 20.18 (14.54, 25.19) | 9.406 (9.250, 9.563) | 6.83 (4.56, 10.52) | 83.20 (63.52, 100.08) | 9.037 (8.871, 9.203) |
| Region of the Americas | 12.47 (8.79, 17.60) | 104.59 (73.04, 136.54) | 7.632 (7.557, 7.706) | 37.24 (26.87, 52.95) | 383.33 (240.98, 539.08) | 8.397 (8.295, 8.500) |
| South-East Asia Region | 6.39 (3.29, 15.14) | 55.68 (36.72, 82.51) | 7.689 (7.453, 7.926) | 6.66 (3.28, 15.56) | 63.24 (41.95, 87.08) | 7.726 (7.163, 8.291) |
| Western Pacific Region | 3.71 (2.28, 8.56) | 25.76 (16.27, 41.03) | 6.891 (6.554, 7.229) | 15.12 (9.84, 32.91) | 87.41 (53.36, 138.06) | 6.192 (5.746, 6.640) |
| **World Bank Income region** |  |  |  |  |  |  |
| Low Income | 537.94 (424.68, 662.64) | 1147.88 (947.65, 1409.79) | 2.588 (2.327, 2.851) | 727.85 (534.05, 974.06) | 991.63 (794.73, 1252.11) | 1.020 (0.753, 1.288) |
| Lower Middle Income | 27.40 (21.98, 35.67) | 121.82 (103.42, 146.18) | 5.150 (4.751, 5.550) | 41.20 (32.32, 56.18) | 123.09 (104.17, 145.64) | 3.785 (3.443, 4.129) |
| Upper Middle Income | 6.51 (4.68, 11.00) | 161.45 (139.80, 186.50) | 11.823 (11.437, 12.209) | 19.30 (13.27, 34.30) | 185.17 (158.63, 216.81) | 8.122 (7.688, 8.557) |
| High Income | 5.07 (3.25, 7.82) | 46.53 (27.12, 65.39) | 7.971 (7.842, 8.100) | 18.78 (12.38, 28.23) | 213.91 (127.04, 312.69) | 8.776 (8.623, 8.928) |

Note: AAPC, average annual percentage change; CIs, confidence interval; UIs, uncertainty interval

**Supplementary Table S8**. The HIV/AIDs cases and its changes among older adults by region and sex level in 1990 and 2019.

|  | Female |  |  | Male |  |  |
| --- | --- | --- | --- | --- | --- | --- |
|  | 1990 (95% UI) | 2019 (95% UI) | Percentage change (%) | 1990 (95% UI) | 2019 (95% UI) | Percentage change (%) |
| **Global** | 29366.20 (24827.56, 34654.98) | 370155.36 (326486.86, 421561.91) | 1160.48 | 40929.97 (33708.39, 50382.47) | 411678.34 (341533.30, 485535.24) | 905.81 |
| **SDI region** | | | | | | |
| Low SDI | 17379.10 (14012.89, 21433.26) | 81935.97 (67475.57, 100083.59) | 371.46 | 21008.36 (15556.04, 28435.56) | 57097.31 (46055.89, 70805.55) | 171.78 |
| Low-middle SDI | 6417.38 (4982.75, 8795.54) | 78307.50 (66262.71, 94575.47) | 1120.24 | 8627.27 (6451.14, 12611.83) | 67825.62 (57363.54, 81681.25) | 686.18 |
| Middle SDI | 2652.46 (1965.44, 4326.07) | 159265.04 (138009.31, 184320.41) | 5904.43 | 4559.20 (3131.33, 7868.01) | 129646.92 (111142.37, 152929.49) | 2743.63 |
| High-middle SDI | 813.52 (581.28, 1219.96) | 12675.15 (10876.35, 14638.64) | 1458.07 | 1801.56 (1313.75, 2641.25) | 24610.31 (21800.01, 27886.71) | 1266.05 |
| High SDI | 2074.00 (1333.82, 3207.46) | 37728.27 (21325.78, 54311.67) | 1719.11 | 4890.22 (3251.95, 7328.29) | 132254.36 (75295.64, 196801.66) | 2604.47 |
| **GBD region** | | | | | | |
| Andean Latin America | 91.33 (43.94, 218.58) | 1167.71 (586.30, 1887.31) | 1178.55 | 162.33 (92.07, 372.18) | 2414.37 (1264.23, 3700.68) | 1387.35 |
| Australasia | 8.13 (5.30, 11.97) | 328.01 (220.88, 456.39) | 3936.23 | 72.38 (52.87, 99.74) | 1409.24 (1071.52, 1714.40) | 1846.90 |
| Caribbean | 505.28 (297.05, 845.29) | 3224.78 (2503.94, 4132.03) | 538.22 | 763.87 (457.13, 1256.09) | 3569.74 (2636.22, 4651.36) | 367.33 |
| Central Asia | 0.48 (0.33, 0.84) | 86.41 (55.61, 123.51) | 17938.99 | 8.83 (4.61, 22.56) | 65.81 (44.20, 86.90) | 644.93 |
| Central Europe | 10.69 (7.37, 16.43) | 516.62 (330.56, 732.81) | 4734.53 | 23.75 (15.84, 35.80) | 931.25 (568.11, 1360.47) | 3820.89 |
| Central Latin America | 165.14 (87.26, 362.61) | 3493.12 (2462.07, 4442.29) | 2015.23 | 545.00 (306.06, 1094.12) | 10160.08 (6499.59, 13353.68) | 1764.23 |
| Central Sub-Saharan Africa | 2213.64 (1451.54, 3298.58) | 7718.80 (5714.96, 10373.78) | 248.69 | 1637.42 (1011.28, 2634.74) | 3419.13 (2573.17, 4531.83) | 108.81 |
| East Asia | 1122.43 (677.55, 2676.06) | 15495.91 (7264.48, 29410.57) | 1280.57 | 3198.46 (2057.88, 7321.54) | 46064.54 (22396.72, 81231.86) | 1340.21 |
| Eastern Europe | 14.62 (9.73, 23.94) | 1004.63 (645.08, 1508.74) | 6772.16 | 24.38 (16.14, 39.08) | 1005.07 (586.20, 1512.38) | 4022.01 |
| Eastern Sub-Saharan Africa | 15614.81 (12472.29, 19359.55) | 88988.02 (73939.19, 108505.86) | 469.90 | 20027.96 (14690.43, 26667.68) | 58508.15 (47129.90, 73195.46) | 192.13 |
| High-income Asia Pacific | 22.45 (13.11, 36.00) | 4193.60 (2151.57, 6265.91) | 18577.63 | 76.67 (33.36, 142.34) | 10277.65 (5514.51, 15605.60) | 13305.69 |
| High-income North America | 1637.88 (1024.14, 2586.14) | 24494.41 (12579.81, 37189.78) | 1395.49 | 3651.70 (2339.29, 5567.95) | 93905.80 (48228.20, 143956.28) | 2471.56 |
| North Africa and Middle East | 62.94 (28.72, 171.43) | 950.60 (513.82, 1782.16) | 1410.29 | 210.99 (96.04, 635.77) | 2190.79 (901.85, 5874.25) | 938.32 |
| Oceania | 3.95 (1.02, 18.63) | 491.21 (25.09, 1254.95) | 12324.31 | 8.37 (2.00, 27.87) | 478.34 (23.53, 1496.17) | 5611.82 |
| South Asia | 627.66 (317.93, 1487.50) | 5802.95 (4048.86, 8693.90) | 824.54 | 442.98 (211.84, 1465.30) | 11928.20 (7401.88, 19074.78) | 2592.73 |
| Southeast Asia | 399.98 (180.62, 1021.53) | 21537.88 (13010.71, 34155.11) | 5284.72 | 880.73 (415.78, 2086.33) | 15782.29 (10790.34, 21856.82) | 1691.97 |
| Southern Latin America | 265.36 (165.05, 447.45) | 1826.32 (1455.25, 2270.02) | 588.24 | 403.44 (275.56, 582.39) | 3278.66 (2839.72, 3753.79) | 712.68 |
| Southern Sub-Saharan Africa | 2250.79 (1247.34, 4247.21) | 134385.15 (114761.77, 156147.45) | 5870.57 | 2548.01 (1287.35, 5308.19) | 72743.79 (60087.14, 87644.42) | 2754.92 |
| Tropical Latin America | 103.22 (67.71, 170.10) | 11898.60 (7645.58, 16570.80) | 11427.47 | 186.48 (120.79, 309.02) | 16651.44 (9455.62, 25194.33) | 8829.56 |
| Western Europe | 601.48 (386.21, 934.71) | 11175.09 (7569.16, 14200.00) | 1757.93 | 1430.46 (930.64, 2216.95) | 32831.90 (24575.27, 39785.89) | 2195.20 |
| Western Sub-Saharan Africa | 3643.93 (2661.75, 5222.17) | 31375.53 (25667.51, 38763.27) | 761.03 | 4625.76 (3183.99, 7108.82) | 24062.06 (19418.52, 30487.51) | 420.18 |
| **WHO region** | | | | | | |
| African Region | 23708.68 (19491.45, 28225.24) | 262218.68 (226079.79, 304776.74) | 1006.00 | 28823.56 (22322.26, 36704.76) | 158867.25 (138232.79, 185210.04) | 451.17 |
| Eastern Mediterranean Region | 181.70 (66.34, 374.92) | 1269.31 (659.00, 2774.43) | 598.58 | 482.19 (182.56, 1513.00) | 2472.27 (912.38, 7205.22) | 412.72 |
| European Region | 628.04 (412.88, 959.40) | 12814.70 (9235.25, 15996.40) | 1940.43 | 1494.26 (997.99, 2301.25) | 34942.68 (26674.94, 42029.37) | 2238.46 |
| Region of the Americas | 2705.15 (1907.88, 3819.51) | 45866.11 (32029.42, 59880.85) | 1595.51 | 5558.28 (4010.74, 7901.98) | 129658.41 (81511.31, 182341.23) | 2232.71 |
| South-East Asia Region | 870.58 (448.55, 2062.03) | 25340.18 (16711.56, 37548.55) | 2810.72 | 853.32 (419.97, 1992.20) | 24705.73 (16389.36, 34020.69) | 2795.25 |
| Western Pacific Region | 1194.54 (733.89, 2753.42) | 22257.77 (14059.17, 35452.14) | 1763.29 | 3537.35 (2303.05, 7699.86) | 60534.05 (36949.90, 95605.65) | 1611.28 |
| **World Bank Income region** | | | | | | |
| Low Income | 17649.23 (13933.36, 21740.57) | 88442.04 (73014.65, 108621.69) | 401.11 | 21380.45 (15687.61, 28612.88) | 58659.80 (47012.16, 74068.64) | 174.36 |
| Lower Middle Income | 6379.23 (5118.57, 8305.81) | 73403.82 (62316.52, 88081.70) | 1050.67 | 8222.15 (6449.05, 11211.19) | 62873.84 (53208.66, 74392.29) | 664.69 |
| Upper Middle Income | 2753.89 (1978.88, 4654.50) | 166691.63 (144345.02, 192559.53) | 5952.96 | 5601.16 (3851.88, 9954.83) | 148670.21 (127360.44, 174072.94) | 2554.28 |
| High Income | 2554.10 (1635.95, 3938.94) | 41374.27 (24114.45, 58144.69) | 1519.91 | 5682.83 (3748.50, 8544.98) | 141230.19 (83877.20, 206450.73) | 2385.21 |

Note: UI, uncertainty interval

**Supplementary Table S9**. Average annual percentage changes of prevalence by region and age from 2010 to 2019.

|  | 70-74 years | 75-79 years | 80-84 years | 85-89 years | 90-94 years | 95+ years |
| --- | --- | --- | --- | --- | --- | --- |
| **Global** | 5.743 (5.475, 6.011) | 5.019 (4.688, 5.351) | 9.302 (9.027, 9.578) | 9.480 (9.166, 9.795) | 9.400 (8.643, 10.163) | 9.787 (9.319, 10.256) |
| **SDI region** |  |  |  |  |  |  |
| Low SDI | 1.279 (1.072, 1.487) | 1.401 (1.189, 1.614) | 5.210 (4.843, 5.579) | 5.172 (4.865, 5.480) | 5.698 (5.410, 5.987) | 6.808 (6.430, 7.187) |
| Low-middle SDI | 4.600 (4.201, 5.001) | 3.760 (3.432, 4.088) | 8.958 (8.520, 9.398) | 8.745 (8.068, 9.426) | 8.816 (8.171, 9.466) | 9.764 (9.246, 10.285) |
| Middle SDI | 11.833 (11.501, 12.165) | 7.525 (7.093, 7.958) | 11.647 (10.239, 13.072) | 12.084 (11.675, 12.495) | 12.053 (10.455, 13.675) | 11.656 (11.042, 12.274) |
| High-middle SDI | 9.363 (9.131, 9.596) | 4.333 (3.662, 5.008) | 9.487 (8.935, 10.043) | 9.660 (9.060, 10.263) | 9.552 (8.612, 10.500) | 9.666 (8.511, 10.834) |
| High SDI | 10.990 (10.748, 11.232) | 7.102 (6.796, 7.410) | 12.153 (11.826, 12.481) | 11.962 (11.619, 12.307) | 11.610 (11.310, 11.911) | 11.192 (10.860, 11.525) |
| **GBD region** |  |  |  |  |  |  |
| Andean Latin America | 7.751 (7.525, 7.979) | 2.262 (1.595, 2.933) | 8.228 (7.493, 8.969) | 9.100 (8.479, 9.724) | 9.674 (8.899, 10.453) | 10.542 (9.888, 11.200) |
| Australasia | 9.198 (8.782, 9.616) | 6.325 (5.624, 7.031) | 14.401 (13.668, 15.138) | 14.710 (13.932, 15.492) | 15.151 (14.410, 15.898) | 15.217 (14.560, 15.878) |
| Caribbean | 3.591 (3.098, 4.085) | 2.163 (1.839, 2.489) | 8.187 (7.713, 8.662) | 8.169 (7.561, 8.781) | 7.646 (7.070, 8.225) | 5.268 (4.290, 6.255) |
| Central Asia | 15.995 (15.608, 16.383) | 4.704 (3.887, 5.527) | 5.167 (4.320, 6.022) | 5.302 (4.876, 5.729) | 5.522 (4.826, 6.222) | 5.348 (4.050, 6.661) |
| Central Europe | 13.953 (13.606, 14.302) | 9.116 (8.768, 9.466) | 14.624 (13.829, 15.425) | 14.703 (13.822, 15.591) | 15.252 (14.053, 16.463) | 15.645 (14.902, 16.392) |
| Central Latin America | 12.419 (12.088, 12.751) | 3.188 (2.579, 3.801) | 6.587 (5.795, 7.384) | 6.597 (5.798, 7.401) | 6.478 (5.671, 7.292) | 7.018 (6.163, 7.879) |
| Central Sub-Saharan Africa | 0.591 (0.440, 0.741) | 1.163 (0.915, 1.411) | 4.969 (4.515, 5.426) | 5.220 (4.868, 5.573) | 5.349 (4.971, 5.727) | 5.610 (5.203, 6.019) |
| East Asia | 9.063 (8.647, 9.481) | 2.670 (1.449, 3.905) | 8.352 (6.736, 9.993) | 8.159 (6.468, 9.877) | 7.312 (5.581, 9.071) | 6.608 (4.887, 8.356) |
| Eastern Europe | 17.193 (16.832, 17.555) | 9.074 (8.168, 9.988) | 13.965 (12.652, 15.293) | 14.343 (12.807, 15.900) | 14.466 (13.614, 15.325) | 14.610 (13.589, 15.640) |
| Eastern Sub-Saharan Africa | 2.049 (1.863, 2.235) | 2.160 (1.970, 2.350) | 5.896 (5.493, 6.301) | 5.963 (5.647, 6.279) | 6.215 (5.828, 6.604) | 6.950 (6.616, 7.286) |
| High-income Asia Pacific | 16.441 (16.184, 16.698) | 12.785 (11.655, 13.926) | 17.633 (17.062, 18.208) | 17.613 (16.831, 18.401) | 17.256 (16.519, 17.997) | 17.430 (17.074, 17.787) |
| High-income North America | 10.854 (10.715, 10.993) | 7.116 (6.593, 7.641) | 12.003 (11.680, 12.327) | 12.006 (11.710, 12.303) | 11.962 (11.648, 12.278) | 11.919 (11.646, 12.192) |
| North Africa and Middle East | 6.423 (6.187, 6.659) | 3.130 (2.699, 3.562) | 9.124 (8.727, 9.522) | 9.194 (8.594, 9.797) | 9.599 (9.120, 10.080) | 10.484 (9.944, 11.027) |
| Oceania | 17.767 (17.250, 18.287) | 9.346 (8.161, 10.544) | 14.326 (13.658, 14.998) | 14.205 (13.382, 15.035) | 14.572 (13.904, 15.244) | 16.533 (15.949, 17.120) |
| South Asia | 11.399 (11.062, 11.736) | 1.636 (0.945, 2.331) | 3.517 (2.951, 4.085) | 3.461 (3.030, 3.893) | 3.009 (2.329, 3.692) | 2.569 (2.058, 3.082) |
| Southeast Asia | 16.520 (15.967, 17.075) | 4.568 (4.152, 4.986) | 4.946 (2.640, 7.303) | 5.266 (3.004, 7.578) | 5.199 (3.251, 7.185) | 6.030 (4.977, 7.095) |
| Southern Latin America | 6.954 (6.844, 7.063) | 2.194 (1.651, 2.740) | 8.525 (7.815, 9.238) | 8.455 (7.931, 8.983) | 8.008 (7.151, 8.872) | 7.536 (6.674, 8.405) |
| Southern Sub-Saharan Africa | 9.993 (9.592, 10.396) | 11.572 (10.885, 12.264) | 20.118 (19.217, 21.027) | 21.061 (20.733, 21.391) | 21.816 (21.136, 22.499) | 22.582 (22.179, 22.987) |
| Tropical Latin America | 14.355 (14.097, 14.613) | 10.783 (9.974, 11.598) | 15.385 (13.651, 17.146) | 15.396 (13.763, 17.053) | 15.538 (13.724, 17.380) | 16.030 (14.594, 17.483) |
| Western Europe | 11.482 (11.077, 11.888) | 7.036 (6.583, 7.491) | 13.498 (12.744, 14.257) | 13.593 (12.786, 14.407) | 13.561 (12.788, 14.340) | 13.690 (12.889, 14.497) |
| Western Sub-Saharan Africa | 3.930 (3.706, 4.154) | 4.119 (3.687, 4.553) | 9.211 (8.663, 9.762) | 9.606 (9.233, 9.981) | 10.310 (9.967, 10.654) | 10.900 (10.590, 11.211) |
| **WHO region** |  |  |  |  |  |  |
| African Region | 4.414 (4.166, 4.663) | 4.729 (4.374, 5.085) | 8.956 (8.579, 9.335) | 9.434 (9.208, 9.660) | 10.228 (9.919, 10.538) | 10.895 (10.517, 11.275) |
| Eastern Mediterranean Region | 6.754 (6.514, 6.994) | 0.315 (0.029, 0.602) | 4.494 (4.217, 4.773) | 4.647 (4.250, 5.046) | 4.702 (4.338, 5.067) | 5.552 (4.991, 6.116) |
| European Region | 11.710 (11.479, 11.941) | 7.307 (6.918, 7.698) | 13.447 (12.308, 14.598) | 13.634 (12.751, 14.525) | 13.620 (12.741, 14.506) | 14.105 (13.257, 14.959) |
| Region of the Americas | 10.000 (9.902, 10.097) | 6.113 (5.656, 6.572) | 10.761 (10.495, 11.028) | 10.904 (10.664, 11.145) | 10.949 (10.705, 11.194) | 11.020 (10.765, 11.277) |
| South-East Asia Region | 14.134 (13.475, 14.796) | 3.639 (3.325, 3.954) | 3.938 (2.417, 5.481) | 3.799 (2.418, 5.199) | 3.288 (0.790, 5.847) | 3.964 (2.401, 5.551) |
| Western Pacific Region | 10.088 (9.840, 10.336) | 3.806 (2.507, 5.121) | 9.234 (8.036, 10.444) | 9.038 (8.020, 10.066) | 8.343 (7.046, 9.654) | 7.411 (6.674, 8.152) |
| **World Bank Income region** |  |  |  |  |  |  |
| Low Income | 1.700 (1.419, 1.982) | 1.730 (1.506, 1.953) | 5.417 (5.161, 5.673) | 5.648 (5.330, 5.968) | 6.374 (6.061, 6.687) | 7.457 (7.000, 7.916) |
| Lower Middle Income | 4.522 (4.232, 4.813) | 3.821 (3.547, 4.097) | 8.817 (8.422, 9.213) | 8.712 (8.348, 9.077) | 8.652 (8.254, 9.051) | 10.415 (9.684, 11.151) |
| Upper Middle Income | 12.020 (11.550, 12.491) | 7.713 (7.035, 8.395) | 11.732 (11.095, 12.372) | 12.020 (11.572, 12.471) | 11.904 (11.003, 12.813) | 11.419 (10.818, 12.025) |
| High Income | 10.839 (10.558, 11.120) | 6.733 (6.446, 7.020) | 11.761 (11.440, 12.084) | 11.533 (11.198, 11.868) | 11.189 (10.810, 11.570) | 10.880 (10.498, 11.264) |

Note: data was presented as average annual percentage change and its 95% confidence interval


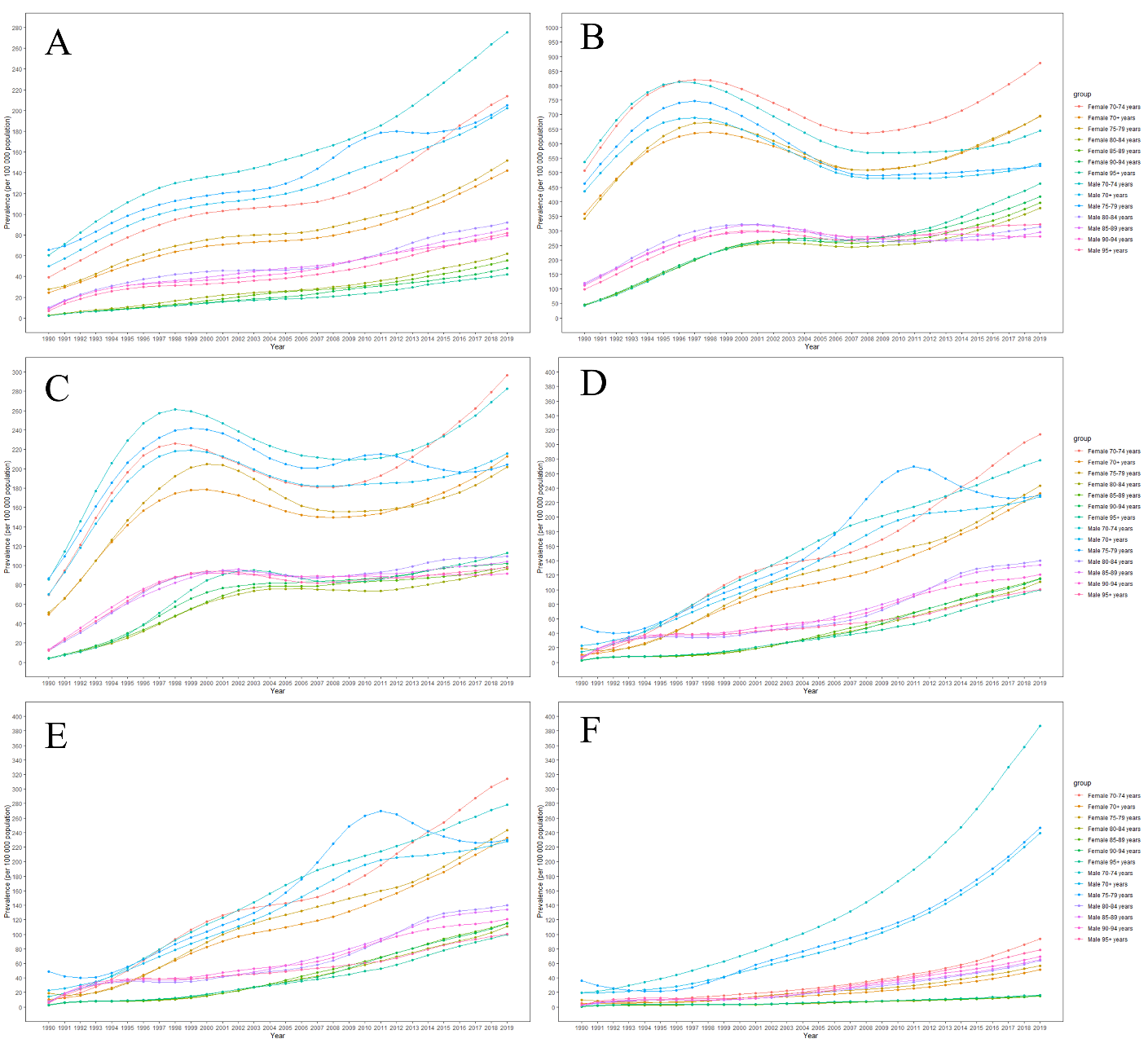


**Supplementary Figure S4**. Sex and age group distribution of HIV/AIDs prevalence by SDI region from1990 to 2019.

(A) Global; (B) Low SDI; (C) Low-middle SDI; (D) Middle SDI; (E) High-middle SDI; (F) High SDI. SDI: Socio-demographic Index

**Supplementary Table S10**. The number of HIV/AIDs deaths and DALYs among older adults from 1990 to 2019.

|  | Deaths |  |  | DALYs |  |  |
| --- | --- | --- | --- | --- | --- | --- |
|  | 1990 (95% UI) | 2019 (95% UI) | Percentage change (%) | 1990 (95% UI) | 2019 (95% UI) | Percentage change (%) |
| **Global** | 3954.08 (2619.34, 5545.47) | 16955.51 (14811.81, 19876.02) | 328.81 | 78835.14 (56016.37, 105782.21) | 363463.28 (313429.57, 434717.84) | 361.04 |
| **SDI region** |  |  |  |  |  |  |
| Low SDI | 2768.05 (1782.19, 3895.48) | 3738.23 (3026.32, 4786.68) | 35.05 | 54189.57 (36757.96, 73941.46) | 78309.48 (63130.82, 101817.40) | 44.51 |
| Low-middle SDI | 679.65 (377.03, 1117.14) | 4264.33 (3520.40, 5480.89) | 527.43 | 13965.63 (8632.81, 22093.11) | 88818.10 (72814.84, 114324.94) | 535.98 |
| Middle SDI | 213.21 (129.51, 295.90) | 7794.37 (6842.63, 8752.89) | 3555.74 | 4701.74 (3394.34, 6128.18) | 160298.62 (140078.59, 184318.35) | 3309.35 |
| High-middle SDI | 67.91 (53.99, 82.06) | 794.18 (662.54, 922.53) | 1069.54 | 1425.72 (1142.42, 1712.55) | 16932.79 (13963.98, 20457.36) | 1087.66 |
| High SDI | 221.34 (217.91, 224.82) | 350.99 (313.13, 549.66) | 58.57 | 4475.17 (4034.54, 5184.05) | 18826.18 (12569.47, 27971.37) | 320.68 |
| **GBD region** |  |  |  |  |  |  |
| Andean Latin America | 15.41 (9.06, 31.72) | 87.42 (46.72, 188.96) | 467.22 | 288.97 (177.46, 597.89) | 1787.40 (1023.86, 3952.51) | 518.53 |
| Australasia | 3.71 (3.53, 3.92) | 1.96 (1.87, 2.07) | -47.10 | 71.13 (64.16, 81.58) | 158.91 (108.07, 223.68) | 123.41 |
| Caribbean | 61.22 (37.53, 102.93) | 186.48 (143.65, 244.49) | 204.60 | 1204.06 (760.68, 2005.31) | 3769.33 (2876.89, 4944.27) | 213.05 |
| Central Asia | 0.16 (0.15, 0.17) | 1.27 (0.77, 2.21) | 692.40 | 3.87 (3.24, 5.15) | 35.81 (22.93, 55.91) | 824.47 |
| Central Europe | 1.34 (1.00, 1.60) | 6.62 (5.73, 9.53) | 393.05 | 27.03 (19.67, 32.04) | 221.98 (161.38, 296.74) | 721.09 |
| Central Latin America | 21.26 (20.48, 22.19) | 228.77 (192.20, 285.41) | 976.22 | 416.36 (375.26, 499.91) | 4907.79 (3998.74, 6037.84) | 1078.75 |
| Central Sub-Saharan Africa | 292.01 (185.23, 437.21) | 475.84 (363.42, 613.13) | 62.95 | 5724.99 (3819.45, 8477.93) | 9526.77 (7313.41, 12367.16) | 66.41 |
| East Asia | 131.51 (51.89, 188.68) | 3462.70 (2574.48, 4261.23) | 2532.95 | 2883.76 (1612.89, 3850.64) | 65493.10 (46936.25, 86246.93) | 2171.10 |
| Eastern Europe | 2.47 (2.42, 2.52) | 18.23 (17.96, 18.51) | 638.89 | 46.70 (42.58, 54.46) | 504.40 (417.15, 584.93) | 980.06 |
| Eastern Sub-Saharan Africa | 2437.29 (1525.19, 3524.57) | 3543.30 (2906.09, 4569.28) | 45.38 | 47785.01 (31723.36, 66603.79) | 75220.19 (60952.44, 96669.59) | 57.41 |
| High-income Asia Pacific | 2.30 (2.11, 2.52) | 21.93 (20.12, 24.07) | 853.96 | 51.42 (41.90, 62.77) | 1707.78 (967.81, 2853.26) | 3221.15 |
| High-income North America | 175.02 (171.89, 178.29) | 220.16 (214.61, 225.82) | 25.79 | 3556.44 (3208.38, 4104.48) | 12435.99 (7913.02, 18919.17) | 249.68 |
| North Africa and Middle East | 12.60 (3.44, 47.03) | 199.41 (88.83, 573.07) | 1482.02 | 269.06 (95.45, 897.40) | 3940.73 (1727.03, 11353.81) | 1364.60 |
| Oceania | 0.25 (0.04, 1.20) | 55.62 (20.91, 152.05) | 22101.75 | 5.49 (1.54, 23.59) | 1097.89 (395.20, 3108.67) | 19891.78 |
| South Asia | 13.76 (2.00, 58.81) | 341.11 (119.76, 1178.04) | 2378.50 | 436.40 (184.25, 1260.45) | 8310.80 (4002.66, 25688.36) | 1804.39 |
| Southeast Asia | 8.17 (5.89, 12.36) | 994.87 (645.97, 1787.12) | 12070.63 | 290.27 (218.94, 438.74) | 22381.86 (14929.84, 38519.17) | 7610.80 |
| Southern Latin America | 12.55 (12.30, 12.83) | 55.23 (53.86, 56.56) | 339.91 | 255.22 (224.79, 308.05) | 1420.67 (1194.33, 1751.83) | 456.64 |
| Southern Sub-Saharan Africa | 195.16 (79.34, 427.21) | 4110.66 (3479.35, 4951.82) | 2006.33 | 4183.83 (1971.73, 8581.47) | 87801.82 (74873.47, 104131.01) | 1998.60 |
| Tropical Latin America | 7.74 (7.48, 8.01) | 328.44 (312.80, 345.90) | 4142.23 | 150.67 (126.99, 205.45) | 8287.07 (6996.75, 9872.06) | 5400.21 |
| Western Europe | 50.05 (48.94, 51.29) | 91.58 (89.07, 94.60) | 82.98 | 1001.90 (875.56, 1218.80) | 4756.50 (3481.44, 6227.82) | 374.75 |
| Western Sub-Saharan Africa | 510.07 (310.69, 801.80) | 2523.92 (2146.39, 2967.56) | 394.82 | 10182.53 (6648.61, 15469.42) | 49696.48 (41598.35, 60254.24) | 388.06 |
| **WHO region** |  |  |  |  |  |  |
| African Region | 3432.95 (2150.89, 4937.21) | 10624.40 (9198.03, 12779.91) | 209.48 | 67844.36 (45545.04, 93316.35) | 221685.03 (191069.41, 267428.60) | 226.76 |
| Eastern Mediterranean Region | 22.84 (4.35, 82.20) | 283.05 (120.30, 905.29) | 1139.52 | 498.19 (147.71, 1616.83) | 5601.29 (2291.59, 17654.40) | 1024.32 |
| European Region | 54.15 (52.97, 55.44) | 128.06 (123.90, 132.86) | 136.48 | 1083.59 (949.23, 1304.19) | 5719.12 (4355.53, 7351.27) | 427.79 |
| Region of the Americas | 284.75 (256.90, 330.72) | 1090.37 (1000.88, 1221.95) | 282.92 | 5716.53 (5016.86, 6797.04) | 32287.14 (26153.59, 39980.07) | 464.80 |
| South-East Asia Region | 10.15 (6.08, 21.07) | 1244.48 (833.88, 2111.46) | 12165.10 | 429.71 (308.68, 737.53) | 28376.33 (20267.17, 46043.11) | 6503.66 |
| Western Pacific Region | 138.42 (58.99, 195.18) | 3558.21 (2656.14, 4363.83) | 2470.66 | 3060.14 (1786.01, 4018.89) | 69255.73 (50353.66, 89868.17) | 2163.16 |
| **World Bank Income region** |  |  |  |  |  |  |
| Low Income | 2783.62 (1792.24, 3964.87) | 3698.14 (3015.68, 4787.56) | 32.85 | 5053.00 (4543.89, 5894.05) | 21035.83 (14475.89, 30456.00) | 316.30 |
| Lower Middle Income | 671.21 (372.89, 1106.81) | 4370.46 (3610.89, 5502.14) | 551.13 | 54559.55 (36741.41, 74998.12) | 78131.42 (63925.54, 101967.92) | 43.20 |
| Upper Middle Income | 244.54 (143.82, 336.06) | 8443.68 (7370.62, 9510.71) | 3352.92 | 13807.14 (8656.02, 21858.75) | 90837.63 (74144.96, 116419.10) | 557.90 |
| High Income | 250.79 (247.44, 254.28) | 429.76 (391.37, 628.94) | 71.36 | 5338.12 (3765.94, 6815.13) | 173179.05 (149798.51, 199127.13) | 3144.20 |

Notes: DALYs: disability-adjusted life years; UI, uncertainty interval

**Supplementary Table S11**. The number of HIV/AIDs deaths and mortality among older adults in 204 countries or territories from 1990 to 2019.

|  | Deaths |  |  | Age-standardized mortality rate (per 100,000 population) | | |
| --- | --- | --- | --- | --- | --- | --- |
| **Countries or territories** | 1990 (95% UI) | 2019 (95% UI) | Percentage change (%) | 1990 (95% UI) | 2019 (95% UI) | AAPC (95% CI) |
| Afghanistan | 1.37 (0.01, 9.05) | 2.99 (0.01, 18.88) | 118.15 | 0.42 (0.00, 2.76) | 0.60 (0.00, 3.79) | 1.263 (1.004, 1.523) |
| Albania | 0.02 (0.01, 0.04) | 0.08 (0.06, 0.11) | 274.33 | 0.02 (0.01, 0.04) | 0.03 (0.02, 0.04) | 1.621 (0.992, 2.253) |
| Algeria | 0.27 (0.01, 1.80) | 13.62 (0.13, 96.53) | 4994.52 | 0.04 (0.00, 0.30) | 0.85 (0.01, 6.05) | 10.672 (9.913, 11.436) |
| American Samoa | 0.00 (0.00, 0.00) | 0.01 (0.00, 0.05) | 1182.00 | 0.12 (0.05, 0.24) | 0.63 (0.06, 2.08) | 5.898 (5.007, 6.797) |
| Andorra | 0.04 (0.01, 0.16) | 0.37 (0.01, 2.16) | 776.41 | 1.46 (0.25, 5.56) | 4.43 (0.16, 25.50) | 3.945 (3.587, 4.305) |
| Angola | 1.95 (0.96, 4.15) | 181.34 (126.27, 255.15) | 9195.23 | 1.48 (0.73, 3.15) | 47.96 (33.39, 67.48) | 12.725 (11.606, 13.856) |
| Antigua and Barbuda | 0.21 (0.20, 0.21) | 0.21 (0.21, 0.22) | 4.33 | 5.66 (5.49, 5.85) | 4.06 (3.93, 4.20) | -0.191 (-1.790, 1.434) |
| Argentina | 4.13 (3.99, 4.28) | 26.05 (24.98, 27.15) | 530.88 | 0.22 (0.22, 0.23) | 0.76 (0.73, 0.79) | 4.559 (3.210, 5.927) |
| Armenia | 0.00 (0.00, 0.00) | 0.01 (0.01, 0.01) | 909.22 | 0.00 (0.00, 0.00) | 0.00 (0.00, 0.01) | 5.855 (5.431, 6.280) |
| Australia | 3.03 (2.86, 3.21) | 1.75 (1.65, 1.85) | -42.30 | 0.25 (0.24, 0.27) | 0.06 (0.06, 0.07) | -5.187 (-6.428, -3.930) |
| Austria | 1.03 (0.93, 1.13) | 1.03 (0.93, 1.14) | 0.57 | 0.13 (0.12, 0.15) | 0.08 (0.08, 0.09) | -1.354 (-4.562, 1.962) |
| Azerbaijan | 0.00 (0.00, 0.01) | 0.08 (0.05, 0.18) | 1948.58 | 0.00 (0.00, 0.00) | 0.03 (0.01, 0.05) | 9.768 (7.868, 11.701) |
| Bahamas | 0.57 (0.55, 0.59) | 1.78 (1.73, 1.83) | 212.07 | 7.08 (6.85, 7.31) | 9.49 (9.22, 9.76) | 1.092 (-0.956, 3.181) |
| Bahrain | 0.01 (0.01, 0.02) | 0.07 (0.05, 0.11) | 499.79 | 0.20 (0.16, 0.27) | 0.31 (0.19, 0.48) | 2.005 (0.850, 3.173) |
| Bangladesh | 0.00 (0.00, 0.00) | 11.19 (0.02, 97.41) | - | 0.00 (0.00, 0.00) | 0.16 (0.00, 1.40) | 190.910 (169.862, 213.600) |
| Barbados | 1.59 (1.54, 1.64) | 1.18 (1.15, 1.22) | -25.71 | 7.45 (7.22, 7.68) | 3.89 (3.77, 4.01) | -1.588 (-2.544, -0.623) |
| Belarus | 0.01 (0.01, 0.01) | 0.04 (0.03, 0.04) | 425.65 | 0.00 (0.00, 0.00) | 0.00 (0.00, 0.00) | 4.882 (4.508, 5.258) |
| Belgium | 2.63 (2.43, 2.85) | 1.63 (1.51, 1.76) | -38.04 | 0.27 (0.25, 0.30) | 0.10 (0.10, 0.11) | -2.983 (-6.670, 0.849) |
| Belize | 0.56 (0.55, 0.58) | 0.83 (0.80, 0.85) | 46.86 | 10.78 (10.47, 11.10) | 6.41 (6.21, 6.61) | -0.654 (-2.501, 1.229) |
| Benin | 0.34 (0.11, 0.78) | 32.24 (20.09, 50.48) | 9265.70 | 0.33 (0.10, 0.76) | 14.80 (9.23, 23.18) | 13.944 (10.711, 17.271) |
| Bermuda | 0.38 (0.37, 0.40) | 0.43 (0.42, 0.45) | 12.60 | 11.00 (10.64, 11.37) | 5.11 (4.96, 5.27) | -1.616 (-3.435, 0.238) |
| Bhutan | 0.09 (0.00, 0.56) | 0.90 (0.00, 6.70) | 889.98 | 0.99 (0.01, 6.16) | 3.13 (0.01, 23.33) | 3.963 (3.613, 4.315) |
| Bolivia (Plurinational State of) | 4.96 (0.13, 21.90) | 15.15 (0.49, 111.09) | 205.38 | 3.28 (0.09, 14.50) | 3.33 (0.11, 24.44) | -0.061 (-0.543, 0.423) |
| Bosnia and Herzegovina | 0.00 (0.00, 0.00) | 0.16 (0.04, 0.50) | 6198.04 | 0.00 (0.00, 0.00) | 0.04 (0.01, 0.14) | 10.444 (7.323, 13.656) |
| Botswana | 14.33 (6.14, 26.50) | 114.73 (82.16, 154.95) | 700.68 | 57.82 (24.78, 106.94) | 215.76 (154.50, 291.39) | 4.476 (3.282, 5.684) |
| Brazil | 7.52 (7.28, 7.76) | 317.72 (304.27, 332.49) | 4126.47 | 0.18 (0.17, 0.18) | 2.43 (2.32, 2.54) | 9.755 (8.121, 11.414) |
| Brunei Darussalam | 0.01 (0.01, 0.01) | 0.18 (0.15, 0.22) | 1465.62 | 0.31 (0.24, 0.40) | 1.84 (1.49, 2.25) | 6.013 (5.298, 6.733) |
| Bulgaria | 0.03 (0.02, 0.03) | 0.08 (0.07, 0.09) | 192.22 | 0.00 (0.00, 0.00) | 0.01 (0.01, 0.01) | 2.591 (-1.821, 7.201) |
| Burkina Faso | 232.00 (134.71, 375.13) | 53.91 (31.19, 91.58) | -76.76 | 117.96 (68.49, 190.74) | 13.78 (7.98, 23.42) | -7.280 (-7.751, -6.806) |
| Burundi | 92.57 (29.78, 276.38) | 28.67 (14.58, 58.50) | -69.03 | 80.66 (25.95, 240.82) | 17.52 (8.91, 35.76) | -5.426 (-6.596, -4.241) |
| Cabo Verde | 0.74 (0.30, 1.57) | 1.13 (0.49, 2.55) | 53.29 | 4.85 (1.99, 10.32) | 5.38 (2.33, 12.16) | 0.212 (-1.096, 1.538) |
| Cambodia | 0.07 (0.02, 0.20) | 20.89 (7.71, 48.23) | 28676.32 | 0.04 (0.01, 0.11) | 3.91 (1.45, 9.04) | 17.204 (14.514, 19.956) |
| Cameroon | 12.73 (6.11, 23.08) | 357.91 (248.67, 496.73) | 2711.53 | 6.90 (3.31, 12.51) | 73.17 (50.83, 101.55) | 8.194 (7.421, 8.973) |
| Canada | 3.56 (3.28, 3.86) | 5.35 (4.91, 5.82) | 50.38 | 0.18 (0.16, 0.19) | 0.12 (0.11, 0.13) | -1.658 (-2.615, -0.692) |
| Central African Republic | 14.28 (4.63, 31.96) | 63.82 (34.72, 102.31) | 346.83 | 35.99 (11.68, 80.51) | 94.76 (51.55, 151.91) | 2.933 (1.871, 4.005) |
| Chad | 12.41 (6.05, 28.65) | 75.45 (47.71, 109.82) | 507.84 | 8.23 (4.01, 19.00) | 30.99 (19.59, 45.10) | 4.749 (3.310, 6.209) |
| Chile | 1.24 (1.19, 1.30) | 11.89 (11.31, 12.47) | 857.36 | 0.23 (0.22, 0.24) | 0.82 (0.78, 0.86) | 4.515 (0.591, 8.593) |
| China | 130.35 (51.79, 186.60) | 3418.52 (2549.81, 4152.05) | 2522.63 | 0.34 (0.14, 0.49) | 3.17 (2.36, 3.85) | 7.819 (6.194, 9.468) |
| Colombia | 2.90 (2.66, 3.15) | 52.47 (47.83, 57.76) | 1711.23 | 0.35 (0.32, 0.38) | 1.70 (1.55, 1.87) | 6.520 (2.843, 10.329) |
| Comoros | 0.01 (0.00, 0.02) | 0.07 (0.00, 0.31) | 1064.82 | 0.05 (0.00, 0.20) | 0.29 (0.00, 1.22) | 5.747 (4.636, 6.870) |
| Congo | 43.01 (24.88, 67.21) | 54.37 (37.23, 81.74) | 26.40 | 97.30 (56.29, 152.03) | 54.57 (37.37, 82.05) | -2.160 (-3.007, -1.306) |
| Cook Islands | 0.00 (0.00, 0.00) | 0.06 (0.00, 0.32) | 7008.15 | 0.14 (0.01, 0.51) | 4.24 (0.04, 23.09) | 12.527 (12.186, 12.868) |
| Costa Rica | 0.62 (0.57, 0.69) | 2.08 (1.88, 2.31) | 234.48 | 0.66 (0.60, 0.72) | 0.72 (0.65, 0.80) | 0.054 (-1.015, 1.135) |
| Croatia | 0.09 (0.08, 0.10) | 0.24 (0.22, 0.27) | 164.15 | 0.03 (0.02, 0.03) | 0.04 (0.04, 0.05) | 0.402 (-1.196, 2.026) |
| Cuba | 0.49 (0.47, 0.51) | 5.32 (5.09, 5.56) | 978.49 | 0.08 (0.08, 0.08) | 0.44 (0.42, 0.46) | 6.438 (4.288, 8.631) |
| Cyprus | 0.02 (0.01, 0.02) | 0.62 (0.51, 0.74) | 3846.77 | 0.03 (0.02, 0.04) | 0.50 (0.41, 0.60) | 10.004 (8.970, 11.048) |
| Czechia | 0.09 (0.08, 0.10) | 0.83 (0.74, 0.92) | 842.76 | 0.01 (0.01, 0.01) | 0.06 (0.05, 0.06) | 7.452 (0.822, 14.519) |
| Cote d'Ivoire | 75.70 (28.20, 208.65) | 186.03 (118.55, 286.27) | 145.74 | 56.67 (21.11, 156.18) | 46.86 (29.86, 72.11) | -1.039 (-2.045, -0.022) |
| Democratic People's Republic of Korea | 1.06 (0.00, 7.98) | 37.17 (0.30, 263.90) | 3419.64 | 0.17 (0.00, 1.25) | 2.05 (0.02, 14.54) | 9.392 (7.726, 11.083) |
| Democratic Republic of the Congo | 229.89 (146.27, 356.66) | 129.92 (77.24, 228.11) | -43.49 | 39.22 (24.95, 60.84) | 9.67 (5.75, 16.97) | -4.877 (-5.442, -4.308) |
| Denmark | 0.01 (0.01, 0.01) | 1.21 (1.09, 1.34) | 16652.76 | 0.00 (0.00, 0.00) | 0.15 (0.13, 0.16) | 16.846 (7.281, 27.264) |
| Djibouti | 0.00 (0.00, 0.01) | 11.05 (6.57, 17.86) | 371726.24 | 0.07 (0.01, 0.23) | 52.89 (31.43, 85.47) | 26.349 (23.725, 29.029) |
| Dominica | 0.22 (0.21, 0.22) | 0.17 (0.16, 0.17) | -22.34 | 4.29 (4.15, 4.44) | 3.10 (2.99, 3.21) | -0.057 (-1.885, 1.805) |
| Dominican Republic | 4.27 (1.28, 10.36) | 40.54 (23.63, 66.13) | 849.94 | 2.29 (0.69, 5.57) | 8.03 (4.68, 13.10) | 4.365 (3.638, 5.098) |
| Ecuador | 0.64 (0.56, 0.74) | 17.98 (15.75, 20.65) | 2705.41 | 0.24 (0.21, 0.27) | 2.14 (1.87, 2.45) | 8.155 (6.812, 9.515) |
| Egypt | 0.52 (0.32, 0.65) | 0.94 (0.65, 1.46) | 81.19 | 0.04 (0.02, 0.05) | 0.04 (0.03, 0.06) | -0.881 (-3.474, 1.783) |
| El Salvador | 2.43 (2.00, 3.14) | 41.74 (14.23, 89.10) | 1618.07 | 1.54 (1.27, 1.99) | 11.34 (3.87, 24.22) | 7.175 (6.684, 7.667) |
| Equatorial Guinea | 0.27 (0.13, 0.55) | 23.91 (14.08, 37.52) | 8808.68 | 3.38 (1.61, 6.96) | 122.77 (72.28, 192.63) | 12.961 (11.783, 14.152) |
| Eritrea | 3.10 (1.32, 7.45) | 15.90 (10.04, 24.69) | 412.18 | 10.62 (4.54, 25.49) | 18.09 (11.42, 28.10) | 1.637 (0.700, 2.583) |
| Estonia | 0.00 (0.00, 0.00) | 0.01 (0.01, 0.01) | 647.02 | 0.00 (0.00, 0.00) | 0.00 (0.00, 0.00) | 5.570 (5.235, 5.906) |
| Eswatini | 0.13 (0.04, 0.40) | 64.01 (39.79, 95.69) | 48997.66 | 1.04 (0.33, 3.18) | 252.59 (157.00, 377.57) | 20.623 (19.070, 22.196) |
| Ethiopia | 125.48 (59.40, 252.23) | 386.18 (263.47, 563.70) | 207.75 | 16.32 (7.72, 32.80) | 20.44 (13.95, 29.84) | 0.543 (-0.178, 1.268) |
| Fiji | 0.03 (0.02, 0.06) | 0.08 (0.04, 0.16) | 123.94 | 0.26 (0.12, 0.47) | 0.26 (0.14, 0.51) | 0.009 (-1.244, 1.278) |
| Finland | 0.10 (0.08, 0.11) | 0.12 (0.11, 0.14) | 24.68 | 0.02 (0.02, 0.02) | 0.01 (0.01, 0.02) | -3.836 (-5.251, -2.400) |
| France | 7.90 (7.44, 8.43) | 10.46 (9.71, 11.25) | 32.31 | 0.15 (0.14, 0.16) | 0.11 (0.10, 0.12) | -1.187 (-3.222, 0.892) |
| Gabon | 2.60 (1.13, 6.70) | 22.48 (14.00, 33.15) | 764.30 | 9.73 (4.21, 25.06) | 52.36 (32.59, 77.19) | 5.531 (3.941, 7.145) |
| Gambia | 0.25 (0.12, 0.52) | 18.67 (11.39, 27.14) | 7294.28 | 1.61 (0.80, 3.29) | 40.06 (24.45, 58.24) | 11.678 (10.537, 12.831) |
| Georgia | 0.00 (0.00, 0.00) | 0.04 (0.03, 0.05) | 1257.92 | 0.00 (0.00, 0.00) | 0.01 (0.01, 0.01) | 9.020 (1.293, 17.338) |
| Germany | 13.35 (12.53, 14.26) | 16.23 (14.86, 17.61) | 21.58 | 0.17 (0.16, 0.18) | 0.12 (0.11, 0.13) | -1.164 (-4.303, 2.077) |
| Ghana | 30.42 (18.33, 50.14) | 234.54 (164.34, 326.13) | 671.00 | 11.95 (7.20, 19.70) | 34.40 (24.11, 47.84) | 3.682 (3.193, 4.172) |
| Greece | 0.46 (0.42, 0.52) | 0.38 (0.34, 0.41) | -18.79 | 0.05 (0.04, 0.05) | 0.02 (0.02, 0.02) | -2.874 (-7.469, 1.951) |
| Greenland | 0.04 (0.03, 0.05) | 0.10 (0.07, 0.14) | 166.83 | 2.97 (2.43, 3.71) | 3.25 (2.10, 4.53) | -0.023 (-0.833, 0.792) |
| Grenada | 0.23 (0.22, 0.24) | 0.12 (0.12, 0.13) | -46.37 | 4.59 (4.44, 4.73) | 2.10 (2.02, 2.17) | -1.913 (-3.150, -0.660) |
| Guam | 0.02 (0.01, 0.03) | 0.25 (0.08, 0.68) | 1572.71 | 0.54 (0.23, 1.02) | 2.45 (0.79, 6.58) | 5.304 (4.893, 5.716) |
| Guatemala | 2.03 (1.83, 2.25) | 5.54 (4.99, 6.14) | 173.64 | 1.32 (1.19, 1.47) | 0.91 (0.82, 1.01) | -2.036 (-4.674, 0.676) |
| Guinea | 4.57 (2.55, 8.08) | 77.97 (52.37, 112.50) | 1606.68 | 2.76 (1.54, 4.89) | 30.01 (20.15, 43.29) | 8.348 (7.409, 9.295) |
| Guinea-Bissau | 0.62 (0.30, 1.17) | 12.90 (6.48, 22.78) | 1994.41 | 3.53 (1.71, 6.68) | 46.09 (23.13, 81.36) | 9.261 (8.399, 10.130) |
| Guyana | 1.94 (1.88, 2.00) | 1.91 (1.85, 1.97) | -1.65 | 11.09 (10.74, 11.42) | 7.00 (6.78, 7.20) | -0.708 (-2.429, 1.044) |
| Haiti | 37.60 (17.17, 74.89) | 106.71 (68.79, 154.10) | 183.82 | 27.45 (12.54, 54.68) | 36.52 (23.55, 52.74) | 0.761 (-0.372, 1.907) |
| Honduras | 0.00 (0.00, 0.00) | 1.65 (0.84, 3.03) | 109185.76 | 0.00 (0.00, 0.00) | 0.53 (0.27, 0.98) | 20.167 (17.399, 23.001) |
| Hungary | 0.13 (0.11, 0.15) | 0.08 (0.07, 0.10) | -35.92 | 0.02 (0.01, 0.02) | 0.01 (0.01, 0.01) | -3.239 (-4.638, -1.819) |
| Iceland | 0.03 (0.02, 0.03) | 0.02 (0.02, 0.02) | -33.66 | 0.15 (0.13, 0.16) | 0.05 (0.05, 0.05) | -3.636 (-4.921, -2.333) |
| India | 3.10 (1.45, 7.15) | 213.18 (114.53, 429.25) | 6784.67 | 0.02 (0.01, 0.04) | 0.38 (0.20, 0.76) | 10.364 (8.803, 11.948) |
| Indonesia | 0.00 (0.00, 0.00) | 41.07 (23.83, 76.54) | - | 0.00 (0.00, 0.00) | 0.46 (0.27, 0.85) | 118.894 (107.266, 131.175) |
| Iran (Islamic Republic of) | 1.01 (0.33, 1.68) | 25.59 (14.85, 47.54) | 2434.30 | 0.11 (0.03, 0.18) | 0.74 (0.43, 1.37) | 6.875 (5.921, 7.837) |
| Iraq | 0.03 (0.01, 0.06) | 0.52 (0.06, 2.66) | 1598.79 | 0.01 (0.00, 0.02) | 0.05 (0.01, 0.27) | 6.817 (6.399, 7.237) |
| Ireland | 0.03 (0.02, 0.03) | 0.03 (0.03, 0.03) | 8.55 | 0.01 (0.01, 0.01) | 0.01 (0.01, 0.01) | -1.965 (-8.180, 4.670) |
| Israel | 1.51 (1.39, 1.62) | 1.93 (1.79, 2.08) | 27.86 | 0.50 (0.46, 0.53) | 0.26 (0.24, 0.28) | -2.715 (-4.135, -1.273) |
| Italy | 6.23 (6.11, 6.36) | 19.01 (18.43, 19.56) | 205.22 | 0.11 (0.11, 0.11) | 0.18 (0.18, 0.19) | 1.401 (-1.020, 3.882) |
| Jamaica | 2.43 (2.33, 2.53) | 6.28 (6.05, 6.51) | 158.35 | 2.09 (2.01, 2.18) | 3.56 (3.43, 3.69) | 2.015 (0.890, 3.152) |
| Japan | 0.79 (0.77, 0.81) | 6.58 (6.41, 6.76) | 735.68 | 0.01 (0.01, 0.01) | 0.02 (0.02, 0.02) | 3.870 (1.387, 6.414) |
| Jordan | 0.00 (0.00, 0.00) | 0.51 (0.24, 0.97) | 75478.14 | 0.00 (0.00, 0.00) | 0.19 (0.09, 0.35) | 18.564 (16.798, 20.357) |
| Kazakhstan | 0.02 (0.01, 0.02) | 0.05 (0.05, 0.06) | 247.14 | 0.00 (0.00, 0.00) | 0.01 (0.01, 0.01) | 3.403 (1.256, 5.595) |
| Kenya | 212.81 (112.11, 337.91) | 828.97 (582.85, 1182.77) | 289.54 | 57.54 (30.31, 91.37) | 93.66 (65.85, 133.63) | 1.430 (0.565, 2.302) |
| Kiribati | 0.00 (0.00, 0.01) | 0.01 (0.00, 0.01) | 58.81 | 0.25 (0.16, 0.39) | 0.24 (0.12, 0.50) | 0.051 (-1.614, 1.743) |
| Kuwait | 0.01 (0.01, 0.01) | 0.01 (0.01, 0.01) | -29.67 | 0.05 (0.04, 0.06) | 0.01 (0.01, 0.01) | -8.122 (-9.831, -6.380) |
| Kyrgyzstan | 0.03 (0.03, 0.04) | 0.26 (0.24, 0.28) | 687.38 | 0.02 (0.02, 0.02) | 0.14 (0.13, 0.16) | 6.583 (4.437, 8.772) |
| Lao People's Democratic Republic | 0.00 (0.00, 0.00) | 12.19 (0.01, 124.71) | - | 0.00 (0.00, 0.00) | 6.77 (0.01, 69.26) | 193.785 (164.654, 226.124) |
| Latvia | 0.02 (0.02, 0.03) | 0.16 (0.14, 0.18) | 557.25 | 0.01 (0.01, 0.01) | 0.06 (0.05, 0.06) | 5.730 (3.232, 8.289) |
| Lebanon | 1.45 (0.05, 9.93) | 2.39 (0.02, 16.13) | 65.00 | 1.43 (0.05, 9.80) | 0.75 (0.01, 5.03) | -2.221 (-2.629, -1.811) |
| Lesotho | 7.95 (3.60, 14.90) | 230.43 (169.01, 301.59) | 2797.36 | 17.03 (7.70, 31.91) | 423.07 (310.31, 553.72) | 11.363 (10.267, 12.471) |
| Liberia | 2.54 (1.07, 5.64) | 21.90 (13.79, 33.07) | 763.13 | 4.35 (1.84, 9.66) | 25.98 (16.36, 39.22) | 6.306 (5.806, 6.809) |
| Libya | 0.13 (0.00, 0.77) | 1.60 (0.01, 8.60) | 1143.79 | 0.15 (0.01, 0.90) | 0.69 (0.00, 3.70) | 5.405 (5.204, 5.606) |
| Lithuania | 0.00 (0.00, 0.00) | 0.02 (0.02, 0.02) | 614.77 | 0.00 (0.00, 0.00) | 0.00 (0.00, 0.00) | 5.400 (5.037, 5.764) |
| Luxembourg | 0.07 (0.06, 0.08) | 0.04 (0.04, 0.05) | -36.07 | 0.20 (0.18, 0.22) | 0.07 (0.06, 0.08) | -3.685 (-4.736, -2.623) |
| Madagascar | 0.01 (0.00, 0.03) | 26.59 (16.33, 40.71) | 296833.95 | 0.00 (0.00, 0.01) | 7.17 (4.40, 10.98) | 29.635 (27.969, 31.323) |
| Malawi | 179.40 (88.19, 316.57) | 260.44 (174.08, 370.18) | 45.17 | 112.72 (55.41, 198.91) | 81.16 (54.25, 115.36) | -1.250 (-1.777, -0.719) |
| Malaysia | 0.26 (0.18, 0.40) | 17.71 (8.91, 26.48) | 6713.12 | 0.06 (0.04, 0.10) | 1.38 (0.70, 2.07) | 10.785 (9.686, 11.894) |
| Maldives | 0.00 (0.00, 0.00) | 0.02 (0.02, 0.03) | 2965.03 | 0.03 (0.02, 0.04) | 0.19 (0.15, 0.23) | 7.002 (6.065, 7.947) |
| Mali | 7.85 (3.24, 20.44) | 78.49 (47.35, 120.08) | 899.88 | 4.44 (1.84, 11.57) | 20.91 (12.61, 31.99) | 5.144 (4.132, 6.165) |
| Malta | 0.05 (0.04, 0.05) | 0.04 (0.04, 0.05) | -6.25 | 0.19 (0.17, 0.21) | 0.07 (0.06, 0.07) | -3.561 (-4.558, -2.554) |
| Marshall Islands | 0.01 (0.00, 0.07) | 0.10 (0.00, 0.96) | 944.71 | 1.31 (0.00, 9.30) | 9.44 (0.01, 87.93) | 6.847 (6.302, 7.395) |
| Mauritania | 3.79 (0.06, 20.24) | 1.31 (0.01, 3.52) | -65.45 | 7.22 (0.12, 38.59) | 1.30 (0.01, 3.50) | -5.744 (-6.522, -4.959) |
| Mauritius | 0.06 (0.05, 0.06) | 0.83 (0.75, 0.91) | 1317.37 | 0.17 (0.16, 0.19) | 0.92 (0.83, 1.01) | 7.038 (-2.760, 17.822) |
| Mexico | 5.93 (5.83, 6.05) | 69.82 (67.99, 71.71) | 1076.61 | 0.28 (0.28, 0.29) | 1.10 (1.07, 1.13) | 4.871 (2.045, 7.776) |
| Micronesia (Federated States of) | 0.02 (0.00, 0.19) | 0.92 (0.00, 8.16) | 3979.72 | 1.11 (0.00, 9.23) | 40.70 (0.05, 360.12) | 12.778 (11.036, 14.546) |
| Monaco | 0.03 (0.01, 0.13) | 0.13 (0.02, 0.45) | 268.16 | 0.69 (0.11, 2.59) | 1.80 (0.25, 6.43) | 3.232 (2.909, 3.556) |
| Mongolia | 0.00 (0.00, 0.00) | 0.44 (0.02, 1.38) | 17359063.33 | 0.00 (0.00, 0.00) | 0.53 (0.02, 1.66) | 66.661 (58.122, 75.661) |
| Montenegro | 0.00 (0.00, 0.01) | 0.04 (0.03, 0.05) | 798.30 | 0.01 (0.01, 0.02) | 0.07 (0.05, 0.08) | 6.151 (3.732, 8.626) |
| Morocco | 2.12 (0.05, 13.69) | 11.11 (0.07, 73.31) | 424.36 | 0.34 (0.01, 2.20) | 0.80 (0.00, 5.30) | 2.986 (2.504, 3.471) |
| Mozambique | 22.58 (11.03, 44.45) | 762.40 (534.48, 1052.82) | 3275.87 | 9.21 (4.50, 18.13) | 170.02 (119.19, 234.79) | 10.152 (8.483, 11.847) |
| Myanmar | 0.36 (0.07, 1.03) | 57.85 (23.87, 123.66) | 15934.35 | 0.04 (0.01, 0.10) | 2.75 (1.13, 5.87) | 15.560 (13.969, 17.174) |
| Namibia | 4.54 (2.41, 7.80) | 98.81 (67.25, 140.97) | 2076.27 | 12.07 (6.40, 20.73) | 143.48 (97.66, 204.71) | 9.087 (7.391, 10.810) |
| Nauru | 0.00 (0.00, 0.00) | 0.00 (0.00, 0.02) | 2742.60 | 0.13 (0.01, 0.50) | 5.33 (0.03, 27.31) | 13.543 (13.230, 13.858) |
| Nepal | 0.00 (0.00, 0.00) | 48.50 (0.20, 427.24) | - | 0.00 (0.00, 0.00) | 4.43 (0.02, 39.04) | 197.801 (151.602, 252.484) |
| Netherlands | 2.17 (1.96, 2.39) | 1.06 (0.97, 1.17) | -51.14 | 0.17 (0.15, 0.19) | 0.05 (0.04, 0.05) | -4.436 (-6.115, -2.727) |
| New Zealand | 0.68 (0.63, 0.73) | 0.22 (0.20, 0.23) | -68.39 | 0.28 (0.26, 0.30) | 0.04 (0.04, 0.04) | -7.348 (-9.173, -5.487) |
| Nicaragua | 0.06 (0.05, 0.07) | 10.89 (2.98, 22.12) | 17156.78 | 0.09 (0.07, 0.10) | 5.06 (1.39, 10.28) | 15.191 (14.212, 16.179) |
| Niger | 3.13 (1.47, 6.47) | 24.27 (14.64, 38.88) | 675.05 | 3.03 (1.42, 6.25) | 7.82 (4.72, 12.53) | 2.973 (1.815, 4.145) |
| Nigeria | 114.08 (71.39, 182.72) | 1221.33 (1027.15, 1424.38) | 970.55 | 5.57 (3.49, 8.93) | 34.59 (29.09, 40.34) | 6.262 (5.524, 7.005) |
| Niue | 0.00 (0.00, 0.00) | 0.00 (0.00, 0.02) | 3631.17 | 0.09 (0.01, 0.34) | 3.95 (0.02, 19.79) | 13.948 (13.290, 14.610) |
| North Macedonia | 0.00 (0.00, 0.00) | 0.03 (0.02, 0.03) | 1599.60 | 0.00 (0.00, 0.00) | 0.02 (0.01, 0.02) | 7.721 (3.442, 12.178) |
| Northern Mariana Islands | 0.00 (0.00, 0.00) | 0.02 (0.00, 0.05) | 3911.12 | 0.08 (0.04, 0.17) | 1.04 (0.08, 3.41) | 8.764 (8.251, 9.280) |
| Norway | 0.53 (0.50, 0.56) | 0.22 (0.21, 0.23) | -58.62 | 0.11 (0.10, 0.12) | 0.03 (0.03, 0.04) | -4.677 (-10.002, 0.964) |
| Oman | 0.02 (0.02, 0.03) | 1.24 (0.68, 2.26) | 5710.08 | 0.09 (0.07, 0.12) | 2.30 (1.26, 4.19) | 11.795 (10.514, 13.090) |
| Pakistan | 10.58 (0.08, 54.09) | 67.34 (0.12, 467.43) | 536.78 | 0.36 (0.00, 1.83) | 1.49 (0.00, 10.34) | 5.084 (4.371, 5.801) |
| Palau | 0.00 (0.00, 0.00) | 0.04 (0.00, 0.21) | 5911.37 | 0.14 (0.01, 0.52) | 4.87 (0.04, 25.45) | 13.071 (12.756, 13.386) |
| Palestine | 0.00 (0.00, 0.00) | 0.01 (0.01, 0.02) | 2389.44 | 0.00 (0.00, 0.00) | 0.01 (0.01, 0.02) | 8.715 (8.179, 9.254) |
| Panama | 2.38 (2.11, 2.66) | 8.59 (7.68, 9.59) | 261.20 | 2.91 (2.59, 3.26) | 3.51 (3.14, 3.92) | 0.625 (-0.359, 1.619) |
| Papua New Guinea | 0.01 (0.00, 0.04) | 48.99 (18.17, 142.51) | 357145.12 | 0.02 (0.00, 0.06) | 29.84 (11.07, 86.80) | 28.242 (25.892, 30.635) |
| Paraguay | 0.22 (0.11, 0.34) | 10.71 (4.07, 21.06) | 4669.56 | 0.19 (0.09, 0.29) | 3.69 (1.40, 7.26) | 11.223 (9.740, 12.725) |
| Peru | 9.81 (7.73, 12.73) | 54.30 (25.88, 99.34) | 453.47 | 1.64 (1.29, 2.13) | 2.94 (1.40, 5.38) | 2.130 (1.288, 2.980) |
| Philippines | 0.13 (0.13, 0.13) | 1.55 (1.31, 1.92) | 1091.11 | 0.01 (0.01, 0.01) | 0.05 (0.04, 0.06) | 5.369 (4.919, 5.821) |
| Poland | 0.06 (0.06, 0.06) | 0.80 (0.76, 0.83) | 1192.01 | 0.00 (0.00, 0.00) | 0.02 (0.02, 0.02) | 7.845 (4.686, 11.099) |
| Portugal | 2.90 (2.66, 3.13) | 16.88 (15.64, 18.27) | 482.44 | 0.34 (0.31, 0.36) | 0.99 (0.92, 1.07) | 2.692 (-1.380, 6.932) |
| Puerto Rico | 5.87 (5.70, 6.05) | 8.84 (8.56, 9.17) | 50.59 | 2.61 (2.54, 2.69) | 1.74 (1.69, 1.81) | -1.185 (-3.056, 0.723) |
| Qatar | 0.00 (0.00, 0.00) | 0.02 (0.02, 0.03) | 48667.07 | 0.00 (0.00, 0.00) | 0.16 (0.12, 0.22) | 16.110 (14.651, 17.587) |
| Republic of Korea | 1.32 (1.14, 1.54) | 13.70 (11.96, 15.84) | 935.10 | 0.10 (0.09, 0.12) | 0.26 (0.23, 0.30) | 2.597 (0.562, 4.674) |
| Republic of Moldova | 0.02 (0.01, 0.02) | 0.19 (0.17, 0.21) | 1152.46 | 0.01 (0.01, 0.01) | 0.06 (0.05, 0.07) | 7.397 (6.572, 8.228) |
| Romania | 0.61 (0.56, 0.67) | 2.63 (2.42, 2.87) | 331.77 | 0.04 (0.04, 0.05) | 0.11 (0.10, 0.12) | 4.003 (1.396, 6.676) |
| Russian Federation | 2.01 (1.99, 2.04) | 15.73 (15.54, 15.95) | 682.41 | 0.02 (0.02, 0.02) | 0.12 (0.11, 0.12) | 6.025 (4.744, 7.320) |
| Rwanda | 23.91 (8.97, 68.62) | 63.77 (41.53, 94.19) | 166.69 | 19.50 (7.31, 55.96) | 27.28 (17.76, 40.29) | 0.801 (0.071, 1.536) |
| Saint Kitts and Nevis | 0.06 (0.00, 0.22) | 0.99 (0.10, 3.57) | 1434.07 | 2.41 (0.12, 8.22) | 37.04 (3.74, 133.48) | 9.734 (9.238, 10.232) |
| Saint Lucia | 0.19 (0.18, 0.20) | 0.19 (0.19, 0.20) | 1.68 | 3.62 (3.49, 3.74) | 1.57 (1.53, 1.62) | -2.040 (-3.798, -0.251) |
| Saint Vincent and the Grenadines | 0.45 (0.44, 0.46) | 0.52 (0.51, 0.54) | 16.41 | 10.18 (9.88, 10.49) | 6.97 (6.74, 7.21) | -0.466 (-3.117, 2.257) |
| Samoa | 0.04 (0.00, 0.28) | 0.50 (0.00, 4.47) | 1028.50 | 1.07 (0.00, 6.81) | 7.36 (0.01, 65.98) | 6.909 (6.148, 7.675) |
| San Marino | 0.02 (0.00, 0.07) | 0.08 (0.01, 0.28) | 367.86 | 0.79 (0.10, 3.18) | 1.79 (0.25, 6.33) | 2.670 (2.267, 3.074) |
| Sao Tome and Principe | 0.01 (0.00, 0.02) | 0.00 (0.00, 0.01) | -41.52 | 0.25 (0.09, 0.49) | 0.11 (0.03, 0.28) | -3.287 (-4.395, -2.167) |
| Saudi Arabia | 1.28 (0.31, 2.57) | 7.45 (2.77, 25.69) | 483.05 | 0.52 (0.13, 1.04) | 1.53 (0.57, 5.28) | 3.679 (3.351, 4.007) |
| Senegal | 3.67 (2.05, 6.72) | 32.11 (19.58, 48.15) | 774.90 | 2.43 (1.35, 4.44) | 9.28 (5.66, 13.91) | 4.477 (3.567, 5.395) |
| Serbia | 0.30 (0.00, 0.54) | 1.53 (0.82, 4.43) | 406.81 | 0.06 (0.00, 0.10) | 0.15 (0.08, 0.43) | 3.315 (2.254, 4.387) |
| Seychelles | 0.00 (0.00, 0.00) | 0.12 (0.08, 0.16) | 3897.54 | 0.08 (0.06, 0.13) | 2.22 (1.55, 2.96) | 11.877 (11.075, 12.686) |
| Sierra Leone | 2.25 (0.94, 5.82) | 43.59 (28.44, 64.12) | 1839.14 | 2.20 (0.92, 5.70) | 26.38 (17.21, 38.80) | 8.537 (7.685, 9.395) |
| Singapore | 0.18 (0.15, 0.21) | 1.46 (1.26, 1.67) | 732.33 | 0.17 (0.14, 0.20) | 0.35 (0.30, 0.40) | 2.273 (-0.320, 4.934) |
| Slovakia | 0.00 (0.00, 0.00) | 0.12 (0.08, 0.19) | 6218.19 | 0.00 (0.00, 0.00) | 0.02 (0.01, 0.03) | 13.025 (7.703, 18.610) |
| Slovenia | 0.00 (0.00, 0.00) | 0.01 (0.01, 0.01) | 871.86 | 0.00 (0.00, 0.00) | 0.00 (0.00, 0.00) | 5.636 (5.021, 6.255) |
| Solomon Islands | 0.05 (0.00, 0.33) | 0.89 (0.00, 8.39) | 1677.64 | 1.18 (0.00, 7.76) | 8.65 (0.01, 81.54) | 7.089 (6.453, 7.728) |
| Somalia | 0.10 (0.01, 0.39) | 29.96 (17.38, 48.73) | 28697.32 | 0.13 (0.01, 0.48) | 13.17 (7.64, 21.42) | 16.098 (14.865, 17.344) |
| South Africa | 29.45 (14.87, 52.32) | 3194.22 (2679.62, 3865.57) | 10746.84 | 2.86 (1.44, 5.08) | 147.88 (124.06, 178.96) | 14.595 (12.579, 16.648) |
| South Sudan | 3.21 (1.18, 8.81) | 58.72 (21.60, 123.84) | 1727.68 | 2.86 (1.05, 7.84) | 38.18 (14.04, 80.52) | 9.339 (8.794, 9.888) |
| Spain | 5.35 (4.98, 5.70) | 13.18 (12.05, 14.39) | 146.58 | 0.16 (0.14, 0.17) | 0.20 (0.18, 0.22) | 0.426 (-1.337, 2.221) |
| Sri Lanka | 0.06 (0.03, 0.10) | 0.48 (0.24, 0.95) | 781.46 | 0.01 (0.01, 0.02) | 0.04 (0.02, 0.07) | 4.134 (3.255, 5.019) |
| Sudan | 2.97 (0.57, 10.71) | 79.65 (35.45, 175.17) | 2577.89 | 0.63 (0.12, 2.27) | 9.50 (4.23, 20.89) | 9.591 (8.630, 10.561) |
| Suriname | 1.25 (1.21, 1.29) | 1.91 (1.85, 1.96) | 52.46 | 9.70 (9.41, 9.98) | 6.05 (5.88, 6.23) | -0.737 (-1.860, 0.400) |
| Sweden | 1.12 (1.04, 1.21) | 0.55 (0.52, 0.59) | -50.65 | 0.10 (0.10, 0.11) | 0.04 (0.03, 0.04) | -3.621 (-7.632, 0.565) |
| Switzerland | 0.01 (0.01, 0.02) | 1.97 (1.80, 2.18) | 13122.44 | 0.00 (0.00, 0.00) | 0.16 (0.15, 0.18) | 13.837 (8.053, 19.930) |
| Syrian Arab Republic | 0.01 (0.01, 0.02) | 0.07 (0.03, 0.14) | 386.93 | 0.01 (0.00, 0.01) | 0.01 (0.01, 0.03) | 2.417 (1.151, 3.700) |
| Taiwan (Province of China) | 0.11 (0.09, 0.14) | 7.01 (5.93, 8.21) | 6222.16 | 0.02 (0.01, 0.02) | 0.31 (0.26, 0.37) | 10.513 (7.764, 13.333) |
| Tajikistan | 0.00 (0.00, 0.00) | 0.10 (0.04, 0.27) | 9396.71 | 0.00 (0.00, 0.00) | 0.07 (0.03, 0.19) | 17.194 (13.924, 20.559) |
| Thailand | 5.33 (4.00, 7.54) | 822.84 (489.17, 1613.31) | 15326.48 | 0.35 (0.26, 0.49) | 14.55 (8.65, 28.52) | 14.260 (11.103, 17.507) |
| Timor-Leste | 0.15 (0.00, 1.24) | 11.28 (0.03, 104.89) | 7284.74 | 1.77 (0.01, 14.38) | 28.53 (0.07, 265.41) | 10.118 (9.485, 10.755) |
| Togo | 2.96 (1.34, 6.68) | 50.14 (30.98, 77.49) | 1594.80 | 5.75 (2.61, 12.98) | 35.67 (22.04, 55.13) | 6.134 (4.279, 8.023) |
| Tokelau | 0.00 (0.00, 0.00) | 0.00 (0.00, 0.01) | 2223.32 | 0.14 (0.01, 0.53) | 4.30 (0.02, 22.80) | 12.609 (12.295, 12.924) |
| Tonga | 0.01 (0.00, 0.01) | 0.09 (0.00, 0.33) | 1453.87 | 0.21 (0.09, 0.43) | 1.96 (0.08, 7.42) | 7.915 (7.287, 8.546) |
| Trinidad and Tobago | 0.74 (0.71, 0.77) | 1.80 (1.73, 1.87) | 143.87 | 1.56 (1.50, 1.63) | 1.72 (1.66, 1.79) | 0.218 (-0.682, 1.127) |
| Tunisia | 0.18 (0.01, 0.89) | 4.66 (0.05, 26.74) | 2524.57 | 0.07 (0.00, 0.37) | 0.71 (0.01, 4.08) | 8.123 (7.795, 8.452) |
| Turkey | 0.18 (0.00, 0.33) | 10.88 (8.50, 13.16) | 6095.98 | 0.01 (0.00, 0.02) | 0.23 (0.18, 0.28) | 10.928 (10.065, 11.797) |
| Turkmenistan | 0.00 (0.00, 0.00) | 0.01 (0.01, 0.01) | 709.78 | 0.00 (0.00, 0.00) | 0.00 (0.00, 0.00) | 5.389 (5.111, 5.668) |
| Tuvalu | 0.00 (0.00, 0.00) | 0.02 (0.00, 0.12) | 6330.47 | 0.12 (0.01, 0.44) | 4.52 (0.02, 24.24) | 13.279 (13.007, 13.553) |
| Uganda | 1093.94 (681.59, 1585.25) | 270.82 (179.26, 395.01) | -75.24 | 369.81 (230.42, 535.90) | 45.43 (30.07, 66.26) | -7.050 (-7.546, -6.552) |
| Ukraine | 0.41 (0.37, 0.45) | 2.08 (1.91, 2.28) | 412.56 | 0.01 (0.01, 0.01) | 0.04 (0.04, 0.05) | 5.181 (3.580, 6.807) |
| United Arab Emirates | 0.01 (0.00, 0.08) | 31.13 (0.02, 232.94) | 235407.89 | 0.14 (0.00, 0.89) | 77.20 (0.04, 577.74) | 24.175 (21.241, 27.179) |
| United Kingdom | 4.42 (4.34, 4.51) | 4.29 (4.23, 4.34) | -3.08 | 0.07 (0.07, 0.07) | 0.05 (0.05, 0.05) | -0.905 (-3.514, 1.775) |
| United Republic of Tanzania | 543.75 (273.04, 930.26) | 475.41 (280.74, 763.80) | -12.57 | 110.50 (55.49, 189.05) | 43.40 (25.63, 69.73) | -3.238 (-4.320, -2.144) |
| United States of America | 171.42 (168.39, 174.61) | 214.70 (209.17, 220.41) | 25.25 | 0.80 (0.79, 0.82) | 0.60 (0.58, 0.61) | -1.286 (-2.700, 0.149) |
| United States Virgin Islands | 0.12 (0.12, 0.13) | 0.42 (0.40, 0.43) | 237.60 | 3.03 (2.93, 3.14) | 3.37 (3.27, 3.49) | 0.410 (-1.226, 2.072) |
| Uruguay | 7.18 (6.96, 7.39) | 17.29 (16.76, 17.87) | 140.70 | 2.92 (2.83, 3.01) | 4.69 (4.54, 4.84) | 1.882 (1.443, 2.324) |
| Uzbekistan | 0.10 (0.10, 0.11) | 0.28 (0.26, 0.30) | 172.22 | 0.02 (0.02, 0.02) | 0.05 (0.05, 0.06) | 3.836 (1.444, 6.284) |
| Vanuatu | 0.03 (0.00, 0.20) | 1.01 (0.00, 8.98) | 2902.36 | 1.20 (0.00, 7.10) | 12.64 (0.01, 112.92) | 8.099 (6.914, 9.297) |
| Venezuela (Bolivarian Republic of) | 4.91 (4.54, 5.27) | 35.99 (32.78, 39.54) | 633.66 | 1.03 (0.96, 1.11) | 2.35 (2.14, 2.59) | 3.024 (2.327, 3.725) |
| Viet Nam | 1.74 (1.27, 2.44) | 6.73 (3.93, 11.41) | 287.98 | 0.09 (0.06, 0.12) | 0.16 (0.10, 0.28) | 2.056 (-0.097, 4.256) |
| Yemen | 1.03 (0.01, 6.37) | 4.75 (0.02, 32.62) | 362.48 | 0.53 (0.00, 3.28) | 0.84 (0.00, 5.77) | 1.569 (1.195, 1.944) |
| Zambia | 134.62 (59.58, 245.68) | 321.51 (203.61, 477.39) | 138.83 | 115.83 (51.26, 211.40) | 116.90 (74.03, 173.58) | -0.210 (-1.275, 0.866) |
| Zimbabwe | 138.76 (46.10, 359.46) | 408.46 (262.62, 625.34) | 194.37 | 77.32 (25.69, 200.31) | 141.12 (90.73, 216.05) | 1.739 (0.855, 2.631) |

Note: AAPC, average annual percentage change; CI, confidence interval; UI, uncertainty interval


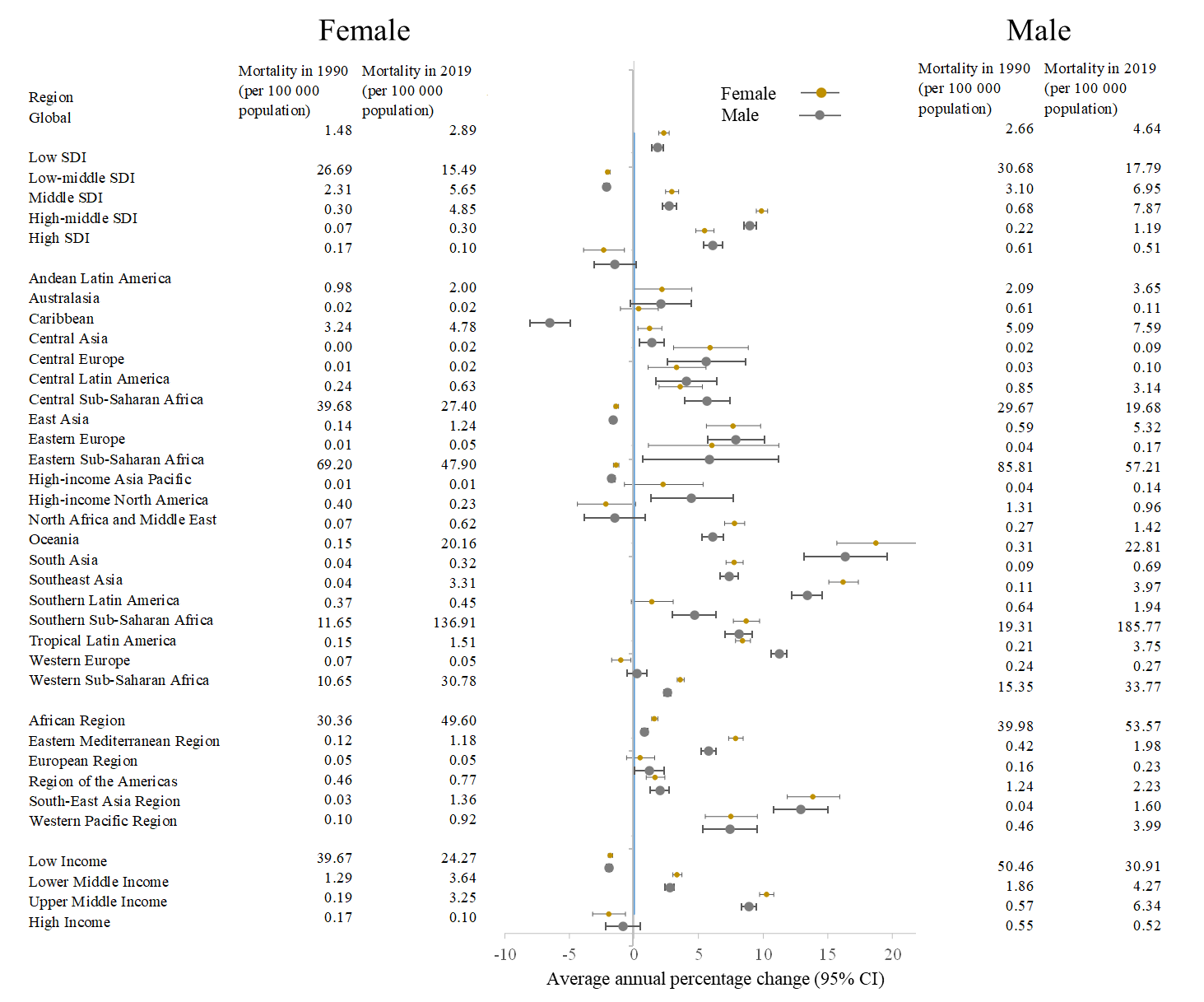


**Supplementary Figure S5**. The mortality and its trend of HIV/AIDs from 1990 to 2019 in global, SDI, GBD, WHO and World Bank income regions by sex.

CI: confidence interval; GBD: Global Burden of Diseases, Injuries, and Risk Factors Study; SDI: Sociodemographic Index; WHO: World Health Organization

**Supplementary Table S12**. The HIV/AIDs mortality and its trends among older adults by region and sex level from 1990 to 2019.

|  | Female |  |  | Male |  |  |
| --- | --- | --- | --- | --- | --- | --- |
|  | 1990 (95% UI) | 2019 (95% UI) | AAPC (95% CI) | 1990 (95% UI) | 2019 (95% UI) | AAPC (95% CI) |
| **Global** | 1.48 (0.99, 2.06) | 2.89 (2.47, 3.44) | 2.322 (1.785, 2.862) | 2.66 (1.73, 3.81) | 4.64 (4.04, 5.44) | 1.832 (1.082, 2.586) |
| Low SDI | 26.69 (17.38, 37.75) | 15.49 (12.38, 20.05) | -2.001 (-2.578, -1.422) | 30.68 (18.67, 45.65) | 17.79 (13.72, 23.30) | -1.945 (-2.359, -1.531) |
| Low-middle SDI | 2.31 (1.31, 3.86) | 5.65 (4.54, 7.32) | 2.956 (2.491, 3.422) | 3.10 (1.65, 5.39) | 6.95 (5.59, 8.97) | 2.709 (2.024, 3.397) |
| Middle SDI | 0.30 (0.20, 0.42) | 4.85 (4.07, 5.77) | 9.903 (9.371, 10.437) | 0.68 (0.39, 0.95) | 7.87 (6.80, 9.18) | 8.695 (8.202, 9.190) |
| High-middle SDI | 0.07 (0.06, 0.07) | 0.30 (0.25, 0.35) | 5.500 (4.925, 6.078) | 0.22 (0.17, 0.28) | 1.19 (0.99, 1.39) | 5.928 (5.257, 6.603) |
| High SDI | 0.17 (0.17, 0.18) | 0.10 (0.09, 0.11) | -2.328 (-3.888, -0.743) | 0.61 (0.59, 0.62) | 0.51 (0.44, 0.87) | -1.312 (-2.828, 0.228) |
| **GBD region** | | | | | | |
| Andean Latin America | 0.98 (0.46, 1.99) | 2.00 (0.64, 5.41) | 2.200 (0.729, 3.693) | 2.09 (1.27, 4.51) | 3.65 (2.25, 7.21) | 2.056 (1.196, 2.923) |
| Australasia | 0.02 (0.01, 0.02) | 0.02 (0.02, 0.02) | 0.401 (-5.227, 6.363) | 0.61 (0.58, 0.65) | 0.11 (0.10, 0.11) | -6.146 (-7.344, -4.932) |
| Caribbean | 3.24 (1.92, 5.76) | 4.78 (3.27, 6.82) | 1.234 (0.297, 2.179) | 5.09 (2.98, 9.19) | 7.59 (5.33, 10.88) | 1.386 (-0.042, 2.834) |
| Central Asia | 0.00 (0.00, 0.00) | 0.02 (0.01, 0.03) | 5.915 (4.099, 7.762) | 0.02 (0.02, 0.02) | 0.09 (0.05, 0.16) | 5.442 (4.774, 6.115) |
| Central Europe | 0.01 (0.01, 0.01) | 0.02 (0.01, 0.02) | 3.317 (0.013, 6.730) | 0.03 (0.02, 0.04) | 0.10 (0.09, 0.14) | 3.944 (2.497, 5.411) |
| Central Latin America | 0.24 (0.23, 0.25) | 0.63 (0.48, 0.87) | 3.596 (2.717, 4.482) | 0.85 (0.81, 0.89) | 3.14 (2.69, 3.87) | 5.503 (4.257, 6.764) |
| Central Sub-Saharan Africa | 39.68 (24.89, 60.25) | 27.40 (20.56, 35.91) | -1.354 (-1.774, -0.933) | 29.67 (17.34, 47.51) | 19.68 (14.59, 26.33) | -1.475 (-1.813, -1.136) |
| East Asia | 0.14 (0.06, 0.20) | 1.24 (0.85, 1.63) | 7.691 (6.030, 9.378) | 0.59 (0.23, 0.85) | 5.32 (4.02, 6.51) | 7.660 (5.661, 9.697) |
| Eastern Europe | 0.01 (0.01, 0.01) | 0.05 (0.05, 0.05) | 6.063 (5.371, 6.759) | 0.04 (0.04, 0.04) | 0.17 (0.17, 0.18) | 5.660 (2.393, 9.033) |
| Eastern Sub-Saharan Africa | 69.20 (43.57, 98.39) | 47.90 (37.33, 64.78) | -1.369 (-1.940, -0.795) | 85.81 (50.25, 129.51) | 57.21 (44.88, 73.29) | -1.555 (-2.207, -0.899) |
| High-income Asia Pacific | 0.01 (0.01, 0.01) | 0.01 (0.01, 0.01) | 2.267 (0.878, 3.676) | 0.04 (0.04, 0.05) | 0.14 (0.13, 0.16) | 4.343 (2.930, 5.774) |
| High-income North America | 0.40 (0.39, 0.41) | 0.23 (0.22, 0.23) | -2.150 (-4.413, 0.166) | 1.31 (1.28, 1.34) | 0.96 (0.93, 0.99) | -1.342 (-3.520, 0.886) |
| North Africa and Middle East | 0.07 (0.03, 0.20) | 0.62 (0.36, 1.17) | 7.799 (6.899, 8.707) | 0.27 (0.06, 1.21) | 1.42 (0.49, 5.14) | 5.920 (5.499, 6.342) |
| Oceania | 0.15 (0.02, 0.77) | 20.16 (7.94, 54.51) | 18.742 (17.730, 19.763) | 0.31 (0.05, 1.56) | 22.81 (7.32, 69.44) | 15.730 (14.561, 16.911) |
| South Asia | 0.04 (0.01, 0.15) | 0.32 (0.13, 1.16) | 7.787 (6.746, 8.838) | 0.09 (0.01, 0.38) | 0.69 (0.22, 2.37) | 7.138 (6.505, 7.775) |
| Southeast Asia | 0.04 (0.03, 0.07) | 3.31 (1.67, 7.39) | 16.211 (14.522, 17.926) | 0.11 (0.08, 0.18) | 3.97 (3.01, 5.69) | 12.929 (11.997, 13.870) |
| Southern Latin America | 0.37 (0.36, 0.38) | 0.45 (0.43, 0.46) | 1.403 (0.766, 2.045) | 0.64 (0.62, 0.65) | 1.94 (1.88, 2.00) | 4.561 (3.627, 5.502) |
| Southern Sub-Saharan Africa | 11.65 (5.01, 25.07) | 136.91 (110.95, 171.16) | 8.706 (7.309, 10.121) | 19.31 (7.15, 48.08) | 185.77 (146.86, 242.96) | 7.852 (6.889, 8.823) |
| Tropical Latin America | 0.15 (0.15, 0.16) | 1.51 (1.40, 1.63) | 8.439 (5.946, 10.990) | 0.21 (0.20, 0.22) | 3.75 (3.53, 3.98) | 10.853 (8.735, 13.011) |
| Western Europe | 0.07 (0.07, 0.08) | 0.05 (0.05, 0.06) | -0.980 (-2.398, 0.460) | 0.24 (0.23, 0.24) | 0.27 (0.26, 0.28) | 0.306 (-1.082, 1.714) |
| Western Sub-Saharan Africa | 10.65 (6.60, 16.40) | 30.78 (25.63, 37.34) | 3.613 (2.899, 4.332) | 15.35 (8.95, 24.65) | 33.77 (27.68, 40.75) | 2.565 (2.049, 3.083) |
| **WHO region** | | | | | | |
| African Region | 30.36 (19.52, 43.12) | 49.60 (41.93, 61.23) | 1.614 (1.259, 1.970) | 39.98 (24.08, 59.41) | 53.57 (45.60, 64.59) | 0.873 (0.270, 1.480) |
| Eastern Mediterranean Region | 0.12 (0.03, 0.41) | 1.18 (0.66, 3.04) | 7.890 (7.456, 8.326) | 0.42 (0.07, 1.66) | 1.98 (0.61, 7.77) | 5.619 (5.208, 6.031) |
| European Region | 0.05 (0.04, 0.05) | 0.05 (0.05, 0.05) | 0.504 (-0.661, 1.683) | 0.16 (0.16, 0.16) | 0.23 (0.22, 0.24) | 1.224 (-0.046, 2.510) |
| Region of the Americas | 0.46 (0.41, 0.56) | 0.77 (0.67, 0.92) | 1.663 (1.151, 2.177) | 1.24 (1.12, 1.45) | 2.23 (2.07, 2.46) | 2.000 (0.801, 3.214) |
| South-East Asia Region | 0.03 (0.02, 0.08) | 1.36 (0.79, 2.77) | 13.870 (12.746, 15.005) | 0.04 (0.03, 0.10) | 1.60 (1.15, 2.77) | 12.437 (11.722, 13.158) |
| Western Pacific Region | 0.10 (0.04, 0.14) | 0.92 (0.64, 1.18) | 7.528 (6.194, 8.879) | 0.46 (0.19, 0.65) | 3.99 (3.03, 4.86) | 7.206 (5.768, 8.665) |
| **World Bank Income region** |  |  |  |  |  |  |
| Low Income | 39.67 (25.81, 56.06) | 24.27 (19.16, 31.74) | -1.810 (-2.170, -1.449) | 50.46 (30.46, 75.67) | 30.91 (23.81, 40.17) | -1.771 (-2.216, -1.323) |
| Lower Middle Income | 1.29 (0.75, 2.03) | 3.64 (2.96, 4.61) | 3.364 (2.694, 4.039) | 1.86 (1.00, 3.12) | 4.27 (3.41, 5.56) | 2.725 (2.096, 3.357) |
| Upper Middle Income | 0.19 (0.12, 0.25) | 3.25 (2.72, 3.82) | 10.281 (9.822, 10.742) | 0.57 (0.31, 0.78) | 6.34 (5.44, 7.35) | 8.639 (8.292, 8.987) |
| High Income | 0.17 (0.16, 0.17) | 0.10 (0.10, 0.11) | -1.921 (-3.219, -0.606) | 0.55 (0.54, 0.56) | 0.52 (0.46, 0.82) | -0.742 (-1.908, 0.438) |

Note: AAPC, average annual percentage change; CI, confidence interval; UI, uncertainty interval

**Supplementary Table S13**. The HIV/AIDs deaths and its changes among older adults by region and sex level in 1990 and 2019.

|  | Female |  |  | Male |  |  |  |
| --- | --- | --- | --- | --- | --- | --- | --- |
|  | 1990 (95% UI) | 2019 (95% UI) | Percentage change (%) | 1990 (95% UI) | 2019 (95% UI) | Percentage change (%) |  |
| **Global** | 1765.24 (1180.43, 2457.72) | 7512.74 (6435.39, 8953.39) | 325.59 | 2188.85 (1420.25, 3130.51) | 9442.77 (8211.27, 11065.30) | 331.40 |  |
| **SDI region** |  |  |  |  |  |  |  |
| Low SDI | 1291.80 (841.34, 1827.18) | 1825.04 (1458.83, 2362.61) | 41.28 | 1476.25 (898.16, 2196.22) | 1913.19 (1475.48, 2505.02) | 29.60 |  |
| Low-middle SDI | 300.09 (170.08, 501.00) | 2079.81 (1673.67, 2695.70) | 593.07 | 379.56 (202.19, 660.47) | 2184.52 (1757.45, 2820.05) | 475.54 |  |
| Middle SDI | 76.94 (50.24, 106.89) | 3319.23 (2791.27, 3953.39) | 4214.04 | 136.27 (78.25, 190.63) | 4475.14 (3866.78, 5218.65) | 3184.05 |  |
| High-middle SDI | 22.80 (20.00, 25.72) | 211.82 (174.83, 246.41) | 829.05 | 45.11 (33.97, 57.67) | 582.36 (485.80, 681.40) | 1191.10 |  |
| High SDI | 71.91 (70.19, 73.49) | 70.63 (67.97, 77.32) | -1.78 | 149.44 (146.44, 152.47) | 280.37 (243.23, 480.63) | 87.62 |  |
| **GBD region** |  |  |  |  |  |  |  |
| Andean Latin America | 5.28 (2.29, 11.39) | 33.20 (10.30, 98.98) | 529.31 | 10.14 (5.86, 22.76) | 54.23 (32.91, 107.49) | 434.91 |  |
| Australasia | 0.14 (0.12, 0.15) | 0.32 (0.27, 0.37) | 134.58 | 3.58 (3.25, 3.95) | 1.65 (1.48, 1.82) | -53.97 |  |
| Caribbean | 25.64 (15.18, 45.75) | 80.88 (55.29, 115.55) | 215.45 | 35.58 (20.88, 63.63) | 105.60 (74.04, 151.92) | 196.77 |  |
| Central Asia | 0.05 (0.04, 0.06) | 0.34 (0.21, 0.61) | 601.39 | 0.11 (0.10, 0.13) | 0.94 (0.52, 1.71) | 730.98 |  |
| Central Europe | 0.35 (0.23, 0.47) | 1.32 (0.96, 2.03) | 278.42 | 1.00 (0.70, 1.23) | 5.31 (4.41, 7.91) | 433.08 |  |
| Central Latin America | 5.06 (4.71, 5.48) | 45.21 (34.04, 63.36) | 793.85 | 16.20 (15.03, 17.53) | 183.56 (154.35, 228.58) | 1033.17 |  |
| Central Sub-Saharan Africa | 173.43 (109.04, 262.84) | 325.97 (244.61, 428.89) | 87.95 | 118.58 (69.07, 190.21) | 149.88 (111.20, 201.62) | 26.39 |  |
| East Asia | 31.15 (10.69, 50.79) | 761.42 (520.16, 1002.98) | 2344.60 | 100.37 (30.81, 157.42) | 2701.28 (2027.16, 3324.76) | 2591.40 |  |
| Eastern Europe | 1.05 (1.02, 1.08) | 7.33 (7.12, 7.56) | 597.75 | 1.42 (1.35, 1.49) | 10.89 (10.50, 11.30) | 669.43 |  |
| Eastern Sub-Saharan Africa | 1104.88 (698.57, 1580.26) | 1783.67 (1391.58, 2409.41) | 61.43 | 1332.41 (780.02, 2011.64) | 1759.63 (1372.34, 2271.40) | 32.06 |  |
| High-income Asia Pacific | 0.45 (0.40, 0.51) | 2.40 (2.19, 2.66) | 438.19 | 1.85 (1.59, 2.16) | 19.52 (16.48, 23.11) | 954.26 |  |
| High-income North America | 58.03 (55.57, 60.57) | 52.03 (49.68, 54.53) | -10.35 | 116.99 (112.53, 121.44) | 168.13 (160.19, 176.46) | 43.71 |  |
| North Africa and Middle East | 2.70 (1.08, 8.08) | 61.12 (34.37, 117.74) | 2162.64 | 9.90 (2.16, 45.44) | 138.29 (47.28, 510.54) | 1296.37 |  |
| Oceania | 0.08 (0.01, 0.44) | 26.46 (10.34, 71.78) | 32591.92 | 0.17 (0.03, 0.86) | 29.16 (9.06, 89.44) | 17094.42 |  |
| South Asia | 3.91 (0.72, 16.33) | 114.34 (44.03, 405.17) | 2821.71 | 9.85 (0.83, 44.82) | 226.77 (72.10, 745.00) | 2202.40 |  |
| Southeast Asia | 2.73 (1.60, 4.00) | 534.57 (268.13, 1200.89) | 19454.91 | 5.44 (3.29, 8.46) | 460.30 (331.76, 693.90) | 8360.32 |  |
| Southern Latin America | 5.73 (5.32, 6.16) | 13.85 (13.03, 14.71) | 141.71 | 6.82 (6.48, 7.18) | 41.37 (39.29, 43.56) | 506.45 |  |
| Southern Sub-Saharan Africa | 93.83 (39.90, 202.28) | 2283.08 (1852.03, 2852.17) | 2333.19 | 101.33 (37.52, 253.21) | 1827.57 (1442.02, 2391.68) | 1703.65 |  |
| Tropical Latin America | 3.70 (3.35, 4.11) | 116.10 (103.39, 130.23) | 3038.02 | 4.04 (3.69, 4.41) | 212.34 (193.95, 233.10) | 5152.90 |  |
| Western Europe | 17.68 (16.40, 19.04) | 19.47 (18.14, 20.96) | 10.11 | 32.37 (30.69, 34.30) | 72.11 (68.02, 76.69) | 122.77 |  |
| Western Sub-Saharan Africa | 229.37 (141.60, 353.42) | 1249.65 (1033.92, 1519.53) | 444.82 | 280.70 (163.15, 455.39) | 1274.27 (1041.59, 1548.66) | 353.96 |  |
| **WHO region** | | | | | | |  |
| African Region | 1600.70 (1029.19, 2273.64) | 5616.61 (4748.79, 6934.43) | 250.89 | 1832.25 (1103.48, 2723.16) | 5007.79 (4263.27, 6038.84) | 173.31 |  |
| Eastern Mediterranean Region | 4.85 (1.26, 16.20) | 102.01 (56.92, 263.89) | 2005.37 | 17.99 (2.82, 72.15) | 181.04 (55.50, 709.02) | 906.33 |  |
| European Region | 19.13 (18.49, 19.84) | 30.86 (29.76, 32.28) | 61.26 | 35.02 (34.08, 36.10) | 97.21 (93.80, 101.17) | 177.57 |  |
| Region of the Americas | 100.36 (88.28, 120.46) | 335.80 (291.97, 402.13) | 234.59 | 184.39 (167.32, 216.02) | 754.58 (699.49, 832.49) | 309.23 |  |
| South-East Asia Region | 4.44 (2.56, 10.70) | 619.78 (357.45, 1260.15) | 13869.21 | 5.71 (3.33, 12.50) | 624.70 (450.59, 1081.94) | 10840.95 |  |
| Western Pacific Region | 31.83 (14.45, 45.36) | 798.03 (552.62, 1020.02) | 2407.49 | 106.59 (44.55, 151.37) | 2760.18 (2095.01, 3367.91) | 2489.53 |  |
| **World Bank Income region** | | | | | | |  |
| Low Income | 1301.45 (846.73, 1839.40) | 1869.63 (1476.15, 2445.50) | 43.66 | 1482.17 (894.85, 2222.72) | 1828.51 (1408.44, 2376.12) | 23.37 |  |
| Lower Middle Income | 299.31 (175.07, 472.86) | 2191.80 (1783.54, 2779.54) | 632.29 | 371.90 (199.90, 622.96) | 2178.66 (1741.04, 2837.50) | 485.81 |  |
| Upper Middle Income | 78.77 (50.42, 107.25) | 3355.46 (2803.77, 3940.12) | 4159.57 | 165.76 (90.75, 225.80) | 5088.22 (4364.27, 5898.58) | 2969.58 |  |
| High Income | 84.00 (82.30, 85.61) | 89.61 (86.89, 96.20) | 6.68 | 166.79 (163.75, 169.85) | 340.15 (302.59, 540.88) | 103.94 |  |

Note: UI, uncertainty interval

**Supplementary Table S14**. Average annual percentage changes of mortality by region and age from 2010 to 2019.

|  | 70-74 years | 75-79 years | 80-84 years | 85-89 years | 90-94 years | 95+ years |
| --- | --- | --- | --- | --- | --- | --- |
| **Global** | 1.946 (1.344, 2.551) | 2.175 (1.642, 2.711) | 4.646 (4.050, 5.244) | 4.313 (3.737, 4.893) | 3.608 (2.974, 4.246) | 2.069 (1.394, 2.749) |
| **SDI region** |  |  |  |  |  |  |
| Low SDI | -2.061 (-2.753, -1.364) | -2.159 (-2.707, -1.607) | 0.176 (-0.316, 0.670) | 0.354 (-0.356, 1.070) | 0.472 (-0.048, 0.995) | 1.509 (0.795, 2.229) |
| Low-middle SDI | 2.847 (2.334, 3.362) | 2.505 (1.921, 3.092) | 5.980 (4.802, 7.172) | 5.781 (4.878, 6.691) | 5.129 (4.094, 6.174) | 4.171 (2.907, 5.451) |
| Middle SDI | 9.855 (9.419, 10.292) | 8.633 (7.498, 9.781) | 9.938 (9.395, 10.484) | 9.622 (9.138, 10.108) | 9.017 (8.359, 9.679) | 7.152 (6.651, 7.656) |
| High-middle SDI | 7.473 (6.593, 8.360) | 5.588 (4.360, 6.830) | 5.468 (4.073, 6.882) | 3.808 (2.930, 4.694) | 3.066 (2.311, 3.826) | 2.336 (1.261, 3.423) |
| High SDI | -0.003 (-1.148, 1.156) | -1.215 (-2.481, 0.068) | -1.408 (-2.641, -0.159) | -2.676 (-4.318, -1.005) | -2.979 (-4.690, -1.238) | -3.707 (-5.686, -1.686) |
| **GBD region** |  |  |  |  |  |  |
| Andean Latin America | 3.697 (2.746, 4.657) | -0.012 (-1.292, 1.283) | 2.479 (0.100, 4.915) | 2.958 (0.491, 5.486) | 3.398 (0.661, 6.209) | 3.106 (0.347, 5.940) |
| Australasia | -5.675 (-6.083, -5.266) | -5.825 (-8.582, -2.984) | -5.377 (-8.663, -1.973) | -4.676 (-7.694, -1.561) | -3.154 (-5.521, -0.729) | 0.732 (-3.396, 5.037) |
| Caribbean | 1.019 (-0.145, 2.196) | 1.785 (0.214, 3.381) | 2.625 (1.430, 3.834) | 1.896 (1.045, 2.755) | 1.212 (0.428, 2.003) | -2.138 (-2.897, -1.373) |
| Central Asia | 7.907 (5.846, 10.008) | 4.948 (4.248, 5.653) | 3.458 (1.917, 5.022) | 5.174 (3.550, 6.823) | 5.007 (3.034, 7.017) | 4.663 (2.992, 6.361) |
| Central Europe | 3.911 (2.420, 5.424) | 3.149 (1.888, 4.425) | 3.799 (2.476, 5.138) | 4.906 (3.712, 6.115) | 4.696 (3.323, 6.086) | 5.834 (4.545, 7.139) |
| Central Latin America | 6.209 (4.611, 7.832) | 4.273 (3.122, 5.437) | 3.085 (2.327, 3.848) | 2.349 (1.552, 3.152) | 2.240 (1.325, 3.164) | 1.429 (0.317, 2.554) |
| Central Sub-Saharan Africa | -1.161 (-1.701, -0.618) | -0.921 (-1.497, -0.341) | 1.086 (0.580, 1.595) | 1.810 (1.221, 2.403) | 1.824 (1.330, 2.321) | 2.271 (1.769, 2.775) |
| East Asia | 8.840 (7.748, 9.943) | 6.189 (3.774, 8.660) | 9.351 (6.794, 11.968) | 9.051 (6.744, 11.407) | 8.129 (5.702, 10.611) | 7.498 (3.751, 11.381) |
| Eastern Europe | 6.346 (3.484, 9.287) | 5.893 (4.042, 7.776) | 5.890 (4.560, 7.237) | 6.459 (5.478, 7.448) | 7.097 (5.461, 8.758) | 6.333 (5.056, 7.626) |
| Eastern Sub-Saharan Africa | -1.429 (-1.811, -1.045) | -1.637 (-2.030, -1.242) | 0.403 (-0.216, 1.026) | 0.799 (0.004, 1.601) | 0.688 (-0.065, 1.445) | 1.436 (0.733, 2.143) |
| High-income Asia Pacific | 4.026 (1.428, 6.691) | 5.400 (4.156, 6.658) | 4.695 (3.995, 5.400) | 5.132 (4.112, 6.162) | 5.122 (4.307, 5.943) | 5.050 (4.104, 6.004) |
| High-income North America | -0.937 (-2.499, 0.649) | -1.381 (-2.614, -0.133) | -2.502 (-4.246, -0.727) | -2.777 (-5.520, 0.046) | -4.093 (-4.674, -3.509) | -3.271 (-5.847, -0.623) |
| North Africa and Middle East | 7.769 (6.999, 8.543) | 4.423 (3.872, 4.977) | 7.505 (6.785, 8.230) | 7.704 (7.258, 8.151) | 8.530 (8.110, 8.952) | 9.261 (8.755, 9.771) |
| Oceania | 20.995 (19.143, 22.876) | 13.966 (12.471, 15.482) | 14.242 (13.050, 15.447) | 13.380 (12.324, 14.446) | 13.881 (12.323, 15.461) | 15.549 (14.404, 16.705) |
| South Asia | 11.607 (10.648, 12.575) | 3.689 (2.517, 4.875) | 1.603 (0.461, 2.757) | 0.621 (-0.271, 1.520) | -0.672 (-1.804, 0.472) | -1.011 (-2.315, 0.311) |
| Southeast Asia | 21.782 (19.348, 24.265) | 10.264 (9.493, 11.040) | 5.795 (5.266, 6.327) | 6.364 (4.891, 7.859) | 6.645 (5.432, 7.872) | 7.080 (5.305, 8.885) |
| Southern Latin America | 5.167 (3.279, 7.091) | 3.144 (2.048, 4.252) | 0.551 (-0.454, 1.565) | -0.162 (-1.312, 1.000) | 2.428 (1.913, 2.945) | 2.628 (1.029, 4.253) |
| Southern Sub-Saharan Africa | 7.513 (6.707, 8.325) | 8.752 (6.829, 10.709) | 14.060 (13.034, 15.095) | 16.176 (13.758, 18.647) | 15.943 (14.653, 17.247) | 18.103 (15.499, 20.766) |
| Tropical Latin America | 13.063 (10.857, 15.313) | 10.166 (8.130, 12.241) | 7.226 (4.103, 10.442) | 2.915 (2.431, 3.402) | 0.074 (-0.965, 1.123) | -0.254 (-1.375, 0.880) |
| Western Europe | 1.022 (-0.786, 2.862) | 0.542 (-0.676, 1.774) | 0.179 (-1.191, 1.567) | -1.953 (-3.017, -0.876) | -1.619 (-2.804, -0.419) | -1.649 (-2.645, -0.643) |
| Western Sub-Saharan Africa | 3.012 (2.347, 3.681) | 2.830 (2.271, 3.392) | 6.373 (5.841, 6.908) | 7.265 (6.590, 7.944) | 7.789 (7.091, 8.491) | 8.582 (7.775, 9.395) |
| **WHO region** |  |  |  |  |  |  |
| African Region | 1.340 (0.584, 2.101) | 1.240 (0.498, 1.989) | 3.732 (3.225, 4.243) | 4.455 (3.862, 5.051) | 4.934 (4.277, 5.596) | 5.723 (5.056, 6.394) |
| Eastern Mediterranean Region | 8.650 (7.898, 9.407) | 3.544 (3.124, 3.966) | 4.606 (3.947, 5.269) | 4.656 (3.598, 5.724) | 4.935 (4.182, 5.694) | 5.591 (4.803, 6.385) |
| European Region | 2.205 (0.621, 3.814) | 1.437 (0.277, 2.610) | 0.849 (-0.775, 2.500) | -0.828 (-1.803, 0.156) | -0.607 (-2.014, 0.821) | -0.319 (-1.285, 0.656) |
| Region of the Americas | 2.242 (1.366, 3.124) | 1.850 (1.114, 2.591) | 1.760 (0.902, 2.624) | 1.276 (0.474, 2.085) | 1.099 (0.251, 1.954) | 0.427 (-0.902, 1.774) |
| South-East Asia Region | 18.601 (17.634, 19.575) | 9.862 (9.061, 10.670) | 5.175 (4.668, 5.684) | 5.008 (4.236, 5.786) | 4.973 (4.194, 5.758) | 5.277 (3.573, 7.008) |
| Western Pacific Region | 9.222 (8.310, 10.141) | 6.343 (4.297, 8.428) | 9.217 (6.999, 11.480) | 8.813 (6.913, 10.746) | 7.577 (5.525, 9.668) | 5.735 (3.801, 7.705) |
| **World Bank Income region** |  |  |  |  |  |  |
| Low Income | -1.717 (-2.083, -1.350) | -2.036 (-2.409, -1.662) | 0.171 (-0.273, 0.616) | 0.676 (0.170, 1.184) | 0.978 (0.485, 1.474) | 2.009 (1.402, 2.619) |
| Lower Middle Income | 2.973 (2.435, 3.514) | 2.909 (1.945, 3.882) | 5.915 (5.320, 6.513) | 6.260 (5.406, 7.121) | 6.335 (5.703, 6.970) | 7.701 (7.027, 8.379) |
| Upper Middle Income | 10.212 (9.879, 10.545) | 8.832 (8.033, 9.638) | 9.662 (8.866, 10.465) | 8.774 (8.294, 9.255) | 7.602 (7.043, 8.163) | 4.780 (4.069, 5.495) |
| High Income | -0.178 (-1.230, 0.885) | -0.700 (-1.722, 0.334) | -1.188 (-2.367, 0.004) | -2.387 (-3.479, -1.282) | -1.833 (-3.296, -0.348) | -2.314 (-3.623, -0.988) |

Note: data was presented as average annual percentage change and its 95% confidence interval.


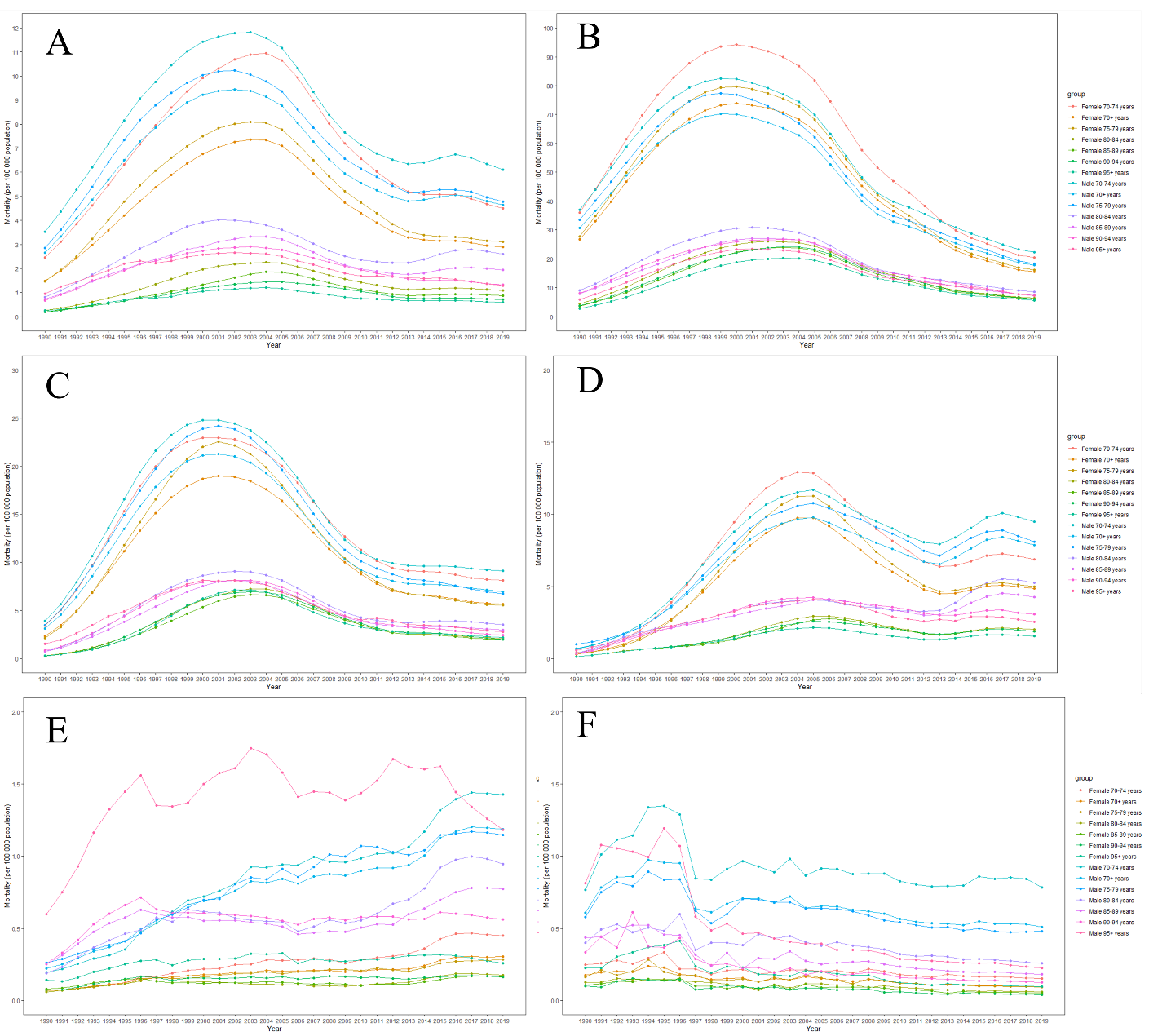


**Supplementary Figure S6**. Sex and age group distribution of HIV/AIDs mortality by SDI region from1990 to 2019.

(A) Global; (B) Low SDI; (C) Low-middle SDI; (D) Middle SDI; (E) High-middle SDI; (F) High SDI. SDI: Socio-demographic Index

**Supplementary Table S15**. The attributable risk factor of HIV/AIDs mortality and DALYs in 2019

|  | Number | Percent | Rate |
| --- | --- | --- | --- |
| **Deaths** |  |  |  |
| Drug use | 1512.60 (1048.210, 2173.202) | 8.91 (6.32, 12.48) | 0.33 (0.23, 0.47) |
| Intimate partner violence | 1393.72 (738.842, 2176.153) | 8.21 (4.44, 12.31) | 0.30 (0.16, 0.47) |
| Unsafe sex | 15381.16 (13402.784, 18097.156) | 90.73 (87.09, 93.34) | 3.32 (2.89, 3.90) |
| **DALYs** |  |  |  |
| Drug use | 30957.54 (20972.67, 44894.65) | 8.50 (6.02, 11.95) | 6.68 (4.52, 9.68) |
| Intimate partner violence | 30275.06 (16116.25, 47271.30) | 8.33 (4.52, 12.45) | 6.53 (3.48, 10.19) |
| Unsafe sex | 331140.56 (285150.21, 397281.03) | 91.12 (87.65, 93.66) | 71.42 (61.50, 85.68) |

**Supplementary Table S16**. The number of HIV/AIDs DALYs and DALY rate among older adults in 204 countries or territories from 1990 to 2019.

|  | Deaths |  |  | Age-standardized DALY rate (per 100,000 population) | | |
| --- | --- | --- | --- | --- | --- | --- |
| **Countries or territories** | 1990 (95% UI) | 2019 (95% UI) | Percentage change (%) | 1990 (95% UI) | 2019 (95% UI) | AAPC (95% CI) |
| Afghanistan | 25.98 (0.44, 174.10) | 59.94 (0.29, 369.90) | 130.7144 | 7.93 (0.14, 53.15) | 12.05 (0.06, 74.36) | 1.459 (1.225, 1.693) |
| Albania | 0.45 (0.30, 0.75) | 1.68 (1.27, 2.33) | 276.1295 | 0.44 (0.30, 0.74) | 0.63 (0.48, 0.88) | 1.211 (0.301, 2.129) |
| Algeria | 5.47 (0.37, 36.56) | 281.23 (6.30, 1934.75) | 5037.199 | 0.90 (0.06, 6.02) | 17.62 (0.39, 121.21) | 10.756 (10.212, 11.302) |
| American Samoa | 0.02 (0.01, 0.04) | 0.28 (0.04, 0.91) | 1098.484 | 2.60 (1.39, 4.54) | 12.52 (1.71, 40.57) | 5.631 (4.940, 6.326) |
| Andorra | 0.77 (0.12, 3.13) | 7.35 (0.27, 41.50) | 849.2691 | 26.38 (3.94, 106.65) | 86.82 (3.20, 489.84) | 4.218 (3.709, 4.729) |
| Angola | 41.86 (21.75, 85.37) | 3550.73 (2416.23, 5044.99) | 8381.913 | 31.72 (16.48, 64.69) | 939.04 (639.00, 1334.21) | 12.407 (11.363, 13.460) |
| Antigua and Barbuda | 3.53 (3.27, 4.14) | 4.14 (3.76, 4.50) | 17.4906 | 97.30 (90.16, 114.15) | 78.63 (71.42, 85.45) | -0.043 (-1.565, 1.503) |
| Argentina | 91.26 (81.33, 103.13) | 785.23 (632.97, 993.06) | 760.441 | 4.93 (4.39, 5.57) | 22.96 (18.51, 29.04) | 5.546 (4.062, 7.050) |
| Armenia | 0.08 (0.02, 0.12) | 0.78 (0.43, 1.38) | 865.6988 | 0.07 (0.01, 0.10) | 0.35 (0.19, 0.61) | 5.441 (4.533, 6.357) |
| Australia | 58.22 (51.89, 67.23) | 146.42 (97.73, 209.74) | 151.5082 | 4.81 (4.29, 5.56) | 5.23 (3.49, 7.49) | 0.274 (-0.782, 1.342) |
| Austria | 22.09 (16.53, 30.85) | 121.47 (56.60, 232.19) | 449.8997 | 2.89 (2.16, 4.04) | 9.87 (4.60, 18.87) | 4.376 (3.304, 5.459) |
| Azerbaijan | 0.09 (0.04, 0.13) | 2.02 (1.35, 3.68) | 2252.149 | 0.04 (0.02, 0.06) | 0.62 (0.41, 1.12) | 9.897 (8.199, 11.621) |
| Bahamas | 11.13 (9.73, 13.61) | 33.69 (30.92, 35.80) | 202.7549 | 138.42 (120.98, 169.28) | 179.96 (165.16, 191.23) | 1.061 (-0.673, 2.826) |
| Bahrain | 0.23 (0.18, 0.30) | 1.53 (0.99, 2.36) | 570.9638 | 3.89 (3.03, 5.06) | 6.56 (4.21, 10.08) | 2.326 (1.078, 3.590) |
| Bangladesh | 0.00 (0.00, 0.00) | 249.13 (29.18, 1923.79) | - | 0.02 (0.03, 0.01) | 3.58 (0.42, 27.62) | 20.567 (19.135, 22.017) |
| Barbados | 27.58 (25.10, 32.08) | 24.47 (21.32, 27.74) | -11.275 | 129.03 (117.46, 150.12) | 80.41 (70.06, 91.17) | -1.277 (-2.042, -0.505) |
| Belarus | 0.15 (0.12, 0.21) | 3.84 (2.48, 5.19) | 2393.323 | 0.02 (0.02, 0.03) | 0.41 (0.27, 0.56) | 10.725 (9.772, 11.686) |
| Belgium | 48.80 (43.86, 56.26) | 260.19 (110.04, 491.75) | 433.1405 | 5.07 (4.56, 5.85) | 16.71 (7.07, 31.58) | 4.095 (0.244, 8.093) |
| Belize | 9.58 (9.04, 10.81) | 15.72 (14.29, 17.20) | 64.13827 | 183.61 (173.17, 207.11) | 122.00 (110.84, 133.41) | -0.582 (-2.119, 0.979) |
| Benin | 9.54 (4.77, 17.65) | 658.10 (417.29, 1016.69) | 6801.792 | 9.26 (4.63, 17.14) | 302.14 (191.58, 466.77) | 12.848 (10.017, 15.752) |
| Bermuda | 6.71 (6.23, 7.73) | 7.37 (6.84, 7.71) | 9.792566 | 192.24 (178.28, 221.45) | 87.09 (80.84, 91.14) | -2.333 (-3.927, -0.712) |
| Bhutan | 1.91 (0.09, 10.81) | 17.64 (0.15, 129.90) | 821.3131 | 20.88 (0.98, 117.91) | 61.41 (0.51, 452.34) | 3.723 (3.427, 4.020) |
| Bolivia (Plurinational State of) | 89.07 (6.71, 381.98) | 308.70 (15.80, 2214.72) | 246.5617 | 58.97 (4.44, 252.88) | 67.93 (3.48, 487.35) | 0.420 (0.028, 0.813) |
| Bosnia and Herzegovina | 0.05 (0.04, 0.06) | 3.82 (1.22, 10.96) | 7700.935 | 0.03 (0.03, 0.04) | 1.08 (0.34, 3.09) | 11.201 (8.270, 14.212) |
| Botswana | 300.78 (147.24, 520.75) | 2505.49 (1807.13, 3356.38) | 733.0065 | 1213.62 (594.11, 2101.21) | 4711.63 (3398.35, 6311.74) | 4.660 (3.566, 5.766) |
| Brazil | 146.28 (122.83, 200.87) | 8062.66 (6877.85, 9473.79) | 5411.976 | 3.46 (2.90, 4.75) | 61.60 (52.55, 72.38) | 10.832 (4.437, 17.619) |
| Brunei Darussalam | 0.25 (0.20, 0.30) | 4.10 (3.19, 5.29) | 1542.263 | 6.73 (5.42, 8.15) | 41.35 (32.12, 53.28) | 6.164 (5.494, 6.839) |
| Bulgaria | 0.49 (0.42, 0.57) | 4.63 (3.17, 5.89) | 854.2043 | 0.07 (0.06, 0.09) | 0.45 (0.31, 0.57) | 6.664 (5.688, 7.648) |
| Burkina Faso | 4476.01 (2660.82, 7159.53) | 1127.60 (686.22, 1873.89) | -74.8079 | 2275.91 (1352.94, 3640.40) | 288.35 (175.48, 479.20) | -6.935 (-7.429, -6.439) |
| Burundi | 1870.55 (625.11, 5648.40) | 626.10 (352.30, 1201.55) | -66.5289 | 1629.86 (544.67, 4921.57) | 382.70 (215.34, 734.45) | -5.137 (-6.219, -4.043) |
| Cabo Verde | 14.53 (6.15, 31.43) | 22.79 (10.74, 51.84) | 56.88328 | 95.67 (40.51, 207.00) | 108.78 (51.26, 247.45) | 0.232 (-1.120, 1.603) |
| Cambodia | 3.52 (2.03, 6.34) | 559.80 (292.65, 1026.70) | 15794.44 | 1.94 (1.12, 3.49) | 104.91 (54.84, 192.41) | 14.635 (13.520, 15.762) |
| Cameroon | 264.59 (138.04, 457.50) | 6903.20 (4776.97, 9767.47) | 2509.02 | 143.46 (74.84, 248.05) | 1411.20 (976.54, 1996.74) | 7.950 (7.242, 8.662) |
| Canada | 75.12 (63.13, 92.49) | 549.21 (352.36, 819.54) | 631.1461 | 3.73 (3.14, 4.60) | 12.21 (7.84, 18.23) | 3.968 (2.860, 5.088) |
| Central African Republic | 307.47 (120.30, 637.69) | 1266.19 (698.32, 2020.02) | 311.8127 | 774.65 (303.09, 1606.64) | 1880.07 (1036.89, 2999.38) | 2.725 (1.793, 3.666) |
| Chad | 255.85 (134.67, 578.47) | 1510.73 (980.64, 2210.03) | 490.4821 | 169.63 (89.29, 383.55) | 620.43 (402.73, 907.62) | 4.678 (3.293, 6.082) |
| Chile | 26.37 (24.34, 28.61) | 337.02 (276.17, 441.50) | 1178.194 | 4.94 (4.56, 5.36) | 23.37 (19.15, 30.62) | 6.285 (2.394, 10.323) |
| China | 2853.38 (1603.61, 3810.07) | 64591.27 (46687.45, 83710.28) | 2163.674 | 7.46 (4.19, 9.96) | 59.83 (43.24, 77.53) | 7.417 (6.219, 8.629) |
| Colombia | 54.15 (48.45, 60.91) | 1056.57 (927.89, 1205.11) | 1851.169 | 6.52 (5.84, 7.34) | 34.16 (30.00, 38.96) | 5.740 (2.290, 9.307) |
| Comoros | 1.91 (1.14, 2.74) | 2.59 (0.76, 8.80) | 35.60749 | 16.47 (9.80, 23.61) | 10.22 (3.00, 34.71) | -1.738 (-2.956, -0.505) |
| Congo | 804.73 (483.69, 1234.08) | 1077.43 (747.15, 1638.35) | 33.88641 | 1820.39 (1094.16, 2791.62) | 1081.50 (749.98, 1644.56) | -1.849 (-2.585, -1.108) |
| Cook Islands | 0.02 (0.00, 0.06) | 1.09 (0.02, 5.93) | 6726.511 | 2.62 (0.30, 9.87) | 78.53 (1.10, 426.13) | 12.381 (12.088, 12.674) |
| Costa Rica | 11.86 (10.35, 13.87) | 54.13 (45.81, 63.16) | 356.2606 | 12.49 (10.89, 14.60) | 18.81 (15.91, 21.94) | 0.971 (-0.861, 2.836) |
| Croatia | 1.73 (1.52, 2.02) | 10.33 (6.76, 14.99) | 497.937 | 0.53 (0.47, 0.62) | 1.75 (1.14, 2.54) | 3.882 (1.414, 6.409) |
| Cuba | 9.31 (8.59, 10.87) | 162.22 (123.78, 234.24) | 1642.473 | 1.48 (1.36, 1.72) | 13.39 (10.21, 19.33) | 8.046 (5.946, 10.188) |
| Cyprus | 0.33 (0.27, 0.42) | 12.90 (10.70, 15.49) | 3794.536 | 0.64 (0.53, 0.80) | 10.42 (8.64, 12.51) | 9.975 (9.286, 10.669) |
| Czechia | 2.07 (1.80, 2.38) | 25.87 (18.55, 36.44) | 1149.329 | 0.26 (0.23, 0.30) | 1.76 (1.26, 2.48) | 7.328 (5.995, 8.676) |
| Cote d'Ivoire | 1521.76 (560.94, 4256.67) | 3723.85 (2406.98, 5773.85) | 144.7057 | 1139.10 (419.88, 3186.29) | 938.02 (606.31, 1454.41) | -1.264 (-2.201, -0.319) |
| Democratic People's Republic of Korea | 27.22 (4.62, 162.10) | 771.33 (51.20, 5736.99) | 2733.731 | 4.28 (0.73, 25.50) | 42.51 (2.82, 316.18) | 8.303 (6.930, 9.695) |
| Democratic Republic of the Congo | 4511.63 (2948.69, 6858.54) | 2705.80 (1649.83, 4581.90) | -40.026 | 769.60 (502.99, 1169.93) | 201.35 (122.77, 340.96) | -4.671 (-5.177, -4.163) |
| Denmark | 8.21 (3.79, 15.18) | 86.63 (54.86, 136.31) | 954.8521 | 1.47 (0.68, 2.71) | 10.56 (6.68, 16.61) | 8.779 (6.046, 11.583) |
| Djibouti | 0.10 (0.04, 0.26) | 213.78 (126.13, 359.29) | 204017.6 | 2.42 (0.94, 6.09) | 1023.28 (603.73, 1719.79) | 23.468 (21.486, 25.482) |
| Dominica | 3.85 (3.61, 4.23) | 3.24 (2.87, 3.64) | -16.0054 | 76.14 (71.32, 83.58) | 59.58 (52.74, 67.09) | -0.618 (-2.387, 1.183) |
| Dominican Republic | 90.45 (32.36, 206.91) | 818.45 (484.88, 1332.29) | 804.8126 | 48.64 (17.40, 111.26) | 162.12 (96.04, 263.90) | 4.192 (3.608, 4.780) |
| Ecuador | 16.36 (13.34, 20.00) | 357.36 (306.40, 410.98) | 2084.625 | 6.01 (4.91, 7.35) | 42.47 (36.42, 48.85) | 7.706 (6.458, 8.969) |
| Egypt | 10.89 (7.82, 13.29) | 26.85 (20.35, 34.72) | 146.6467 | 0.86 (0.62, 1.05) | 1.05 (0.79, 1.35) | 0.676 (-0.998, 2.379) |
| El Salvador | 45.32 (36.26, 59.41) | 846.01 (306.53, 1739.49) | 1766.651 | 28.74 (22.99, 37.67) | 229.97 (83.32, 472.85) | 7.531 (7.060, 8.005) |
| Equatorial Guinea | 5.78 (3.07, 11.18) | 472.99 (273.57, 739.95) | 8081.186 | 72.82 (38.61, 140.85) | 2428.49 (1404.61, 3799.12) | 12.716 (11.579, 13.864) |
| Eritrea | 64.73 (28.51, 156.31) | 313.24 (197.22, 504.85) | 383.9556 | 221.58 (97.61, 535.11) | 356.47 (224.43, 574.51) | 1.461 (0.622, 2.308) |
| Estonia | 0.03 (0.03, 0.04) | 0.78 (0.44, 1.31) | 2240.392 | 0.03 (0.02, 0.03) | 0.42 (0.24, 0.71) | 9.678 (9.157, 10.203) |
| Eswatini | 5.63 (3.32, 10.96) | 1414.29 (900.70, 2057.99) | 25021.3 | 44.99 (26.51, 87.54) | 5580.68 (3554.11, 8120.68) | 17.911 (16.633, 19.203) |
| Ethiopia | 2628.68 (1301.41, 5164.06) | 7787.40 (5407.86, 11430.58) | 196.2475 | 341.84 (169.24, 671.55) | 412.25 (286.28, 605.11) | 0.425 (-0.218, 1.072) |
| Fiji | 0.75 (0.46, 1.22) | 2.22 (1.36, 4.14) | 194.3059 | 5.54 (3.38, 8.94) | 7.26 (4.46, 13.56) | 0.823 (-0.078, 1.733) |
| Finland | 2.07 (1.77, 2.43) | 14.44 (8.50, 23.20) | 598.2254 | 0.45 (0.39, 0.53) | 1.63 (0.96, 2.63) | 3.460 (-0.687, 7.780) |
| France | 140.29 (118.62, 176.92) | 725.42 (502.96, 976.38) | 417.0801 | 2.69 (2.27, 3.39) | 7.68 (5.33, 10.34) | 3.723 (2.601, 4.858) |
| Gabon | 53.52 (24.42, 135.19) | 453.64 (286.80, 670.26) | 747.634 | 200.12 (91.32, 505.51) | 1056.42 (667.89, 1560.88) | 5.687 (4.377, 7.013) |
| Gambia | 5.75 (3.13, 10.87) | 362.29 (222.28, 546.85) | 6201.914 | 36.75 (19.99, 69.49) | 777.51 (477.04, 1173.61) | 11.100 (10.132, 12.077) |
| Georgia | 0.17 (0.13, 0.21) | 3.98 (2.06, 6.30) | 2286.618 | 0.05 (0.04, 0.07) | 1.07 (0.55, 1.69) | 9.906 (4.867, 15.188) |
| Germany | 260.42 (223.32, 314.04) | 879.20 (621.56, 1223.48) | 237.6038 | 3.22 (2.76, 3.89) | 6.63 (4.69, 9.23) | 2.721 (-0.712, 6.273) |
| Ghana | 607.11 (379.26, 979.63) | 4579.17 (3207.73, 6521.64) | 654.256 | 238.47 (148.97, 384.80) | 671.71 (470.54, 956.64) | 3.627 (3.154, 4.102) |
| Greece | 9.03 (8.11, 10.34) | 22.96 (14.77, 35.86) | 154.1155 | 0.95 (0.85, 1.09) | 1.34 (0.86, 2.09) | 1.647 (-0.075, 3.400) |
| Greenland | 0.79 (0.69, 0.91) | 2.49 (1.59, 3.64) | 215.5286 | 61.28 (53.74, 71.17) | 79.36 (50.63, 116.36) | 0.674 (0.022, 1.331) |
| Grenada | 4.00 (3.71, 4.62) | 2.73 (2.31, 3.16) | -31.7583 | 79.34 (73.64, 91.61) | 46.15 (39.10, 53.48) | -1.051 (-2.921, 0.855) |
| Guam | 0.30 (0.16, 0.52) | 4.76 (1.51, 12.86) | 1481.987 | 10.84 (5.80, 18.58) | 46.14 (14.67, 124.70) | 5.076 (4.720, 5.433) |
| Guatemala | 44.99 (39.37, 51.63) | 177.86 (142.12, 230.87) | 295.3157 | 29.39 (25.72, 33.73) | 29.14 (23.29, 37.83) | -0.260 (-2.543, 2.077) |
| Guinea | 98.69 (58.97, 166.73) | 1566.91 (1072.08, 2250.54) | 1487.645 | 59.73 (35.68, 100.90) | 603.00 (412.58, 866.09) | 8.100 (7.253, 8.954) |
| Guinea-Bissau | 14.34 (8.21, 24.84) | 263.56 (136.00, 484.74) | 1737.683 | 82.27 (47.12, 142.46) | 941.52 (485.85, 1731.61) | 8.810 (8.017, 9.608) |
| Guyana | 35.02 (32.59, 40.80) | 57.85 (41.21, 78.30) | 65.17786 | 200.04 (186.15, 233.06) | 212.00 (151.03, 286.94) | 0.325 (-0.645, 1.304) |
| Haiti | 760.49 (348.63, 1489.24) | 2056.77 (1333.32, 2991.98) | 170.4528 | 555.30 (254.56, 1087.41) | 703.97 (456.35, 1024.06) | 0.614 (-0.434, 1.673) |
| Honduras | 0.03 (0.02, 0.04) | 60.33 (44.12, 80.75) | 206351.4 | 0.03 (0.02, 0.04) | 19.57 (14.31, 26.20) | 23.178 (19.775, 26.678) |
| Hungary | 2.55 (2.15, 2.96) | 9.43 (4.23, 12.48) | 269.2723 | 0.30 (0.25, 0.35) | 0.72 (0.32, 0.96) | 2.618 (0.911, 4.354) |
| Iceland | 0.54 (0.47, 0.66) | 0.79 (0.55, 1.11) | 47.17774 | 2.95 (2.55, 3.62) | 2.23 (1.55, 3.11) | -0.995 (-1.626, -0.360) |
| India | 197.96 (133.05, 300.30) | 5718.33 (3669.77, 9685.29) | 2788.572 | 1.21 (0.81, 1.84) | 10.12 (6.49, 17.14) | 7.704 (6.356, 9.070) |
| Indonesia | 8.10 (0.00, 33.85) | 1501.34 (1063.43, 2207.08) | 18435.97 | 0.21 (0.00, 0.89) | 16.71 (11.84, 24.57) | 5.491 (2.807, 8.245) |
| Iran (Islamic Republic of) | 30.18 (18.63, 42.89) | 485.24 (299.63, 877.38) | 1507.996 | 3.14 (1.94, 4.46) | 13.98 (8.63, 25.27) | 5.240 (4.455, 6.031) |
| Iraq | 0.59 (0.16, 1.13) | 10.81 (1.75, 53.94) | 1737.536 | 0.15 (0.04, 0.30) | 1.11 (0.18, 5.55) | 7.037 (6.622, 7.453) |
| Ireland | 0.57 (0.49, 0.67) | 5.13 (3.02, 8.12) | 802.3067 | 0.21 (0.18, 0.25) | 1.06 (0.63, 1.68) | 5.468 (3.422, 7.554) |
| Israel | 29.14 (24.97, 35.88) | 124.01 (65.19, 212.58) | 325.6486 | 9.56 (8.20, 11.78) | 16.55 (8.70, 28.38) | 1.809 (0.497, 3.139) |
| Italy | 118.96 (102.66, 145.53) | 580.12 (459.09, 749.17) | 387.642 | 2.15 (1.85, 2.63) | 5.64 (4.46, 7.28) | 3.183 (1.643, 4.746) |
| Jamaica | 45.97 (42.74, 51.61) | 154.86 (135.74, 175.62) | 236.8788 | 39.52 (36.74, 44.37) | 87.69 (76.86, 99.44) | 2.463 (1.185, 3.757) |
| Japan | 18.19 (15.43, 23.57) | 851.29 (448.22, 1437.32) | 4580.581 | 0.18 (0.16, 0.24) | 3.09 (1.63, 5.21) | 9.650 (8.421, 10.892) |
| Jordan | 0.01 (0.01, 0.02) | 10.93 (5.24, 20.22) | 84580.47 | 0.03 (0.02, 0.04) | 3.96 (1.90, 7.33) | 19.070 (17.175, 20.997) |
| Kazakhstan | 0.27 (0.24, 0.31) | 1.88 (1.40, 2.23) | 597.6075 | 0.04 (0.04, 0.05) | 0.24 (0.18, 0.29) | 5.909 (5.068, 6.756) |
| Kenya | 4339.07 (2603.38, 6582.35) | 16876.54 (11801.13, 24203.32) | 288.9434 | 1173.22 (703.92, 1779.77) | 1906.77 (1333.33, 2734.58) | 1.436 (0.718, 2.160) |
| Kiribati | 0.08 (0.06, 0.11) | 0.16 (0.10, 0.29) | 100.7743 | 5.73 (4.31, 7.95) | 7.04 (4.52, 12.72) | 0.874 (-0.111, 1.870) |
| Kuwait | 0.18 (0.14, 0.23) | 0.69 (0.36, 1.16) | 280.6524 | 0.90 (0.71, 1.14) | 0.74 (0.39, 1.24) | -2.421 (-4.275, -0.531) |
| Kyrgyzstan | 0.82 (0.67, 1.03) | 6.71 (5.21, 8.63) | 720.9026 | 0.53 (0.44, 0.67) | 3.69 (2.87, 4.75) | 6.831 (6.100, 7.568) |
| Lao People's Democratic Republic | 0.02 (0.00, 0.13) | 265.08 (25.30, 2365.78) | 1396700 | 0.02 (0.00, 0.14) | 147.21 (14.05, 1313.82) | 27.335 (21.083, 33.910) |
| Latvia | 0.56 (0.46, 0.71) | 4.54 (3.36, 5.54) | 706.3868 | 0.27 (0.22, 0.34) | 1.61 (1.19, 1.97) | 6.680 (6.274, 7.089) |
| Lebanon | 27.20 (1.37, 181.95) | 47.61 (1.11, 308.49) | 75.04909 | 26.85 (1.36, 179.64) | 14.85 (0.34, 96.26) | -2.057 (-2.683, -1.427) |
| Lesotho | 189.43 (105.86, 318.09) | 4752.41 (3535.71, 6266.99) | 2408.765 | 405.58 (226.64, 681.02) | 8725.53 (6491.64, 11506.31) | 10.901 (9.966, 11.845) |
| Liberia | 50.92 (21.83, 116.29) | 420.56 (263.87, 644.85) | 725.9278 | 87.19 (37.39, 199.13) | 498.72 (312.91, 764.69) | 6.026 (5.506, 6.547) |
| Libya | 2.85 (0.59, 13.87) | 31.37 (1.11, 167.62) | 999.6012 | 3.33 (0.69, 16.20) | 13.49 (0.48, 72.09) | 4.975 (4.756, 5.195) |
| Lithuania | 0.05 (0.04, 0.06) | 2.52 (1.28, 3.55) | 5069.144 | 0.02 (0.02, 0.02) | 0.63 (0.32, 0.89) | 12.836 (12.228, 13.448) |
| Luxembourg | 1.40 (1.21, 1.73) | 3.20 (1.99, 5.38) | 128.7659 | 4.09 (3.54, 5.07) | 5.09 (3.16, 8.56) | 0.653 (0.083, 1.227) |
| Madagascar | 0.25 (0.08, 0.61) | 516.11 (303.50, 818.72) | 204664.7 | 0.11 (0.03, 0.27) | 139.18 (81.85, 220.79) | 28.015 (26.498, 29.551) |
| Malawi | 3610.65 (1899.16, 6309.94) | 6029.75 (4118.16, 8392.41) | 66.99872 | 2268.69 (1193.30, 3964.73) | 1879.04 (1283.34, 2615.32) | -0.700 (-1.466, 0.071) |
| Malaysia | 10.90 (7.23, 16.25) | 384.08 (216.65, 556.14) | 3423.564 | 2.59 (1.72, 3.87) | 29.97 (16.91, 43.40) | 8.693 (7.871, 9.522) |
| Maldives | 0.02 (0.02, 0.03) | 0.49 (0.39, 0.60) | 2355.234 | 0.66 (0.52, 0.84) | 3.76 (3.00, 4.64) | 6.226 (5.236, 7.225) |
| Mali | 161.47 (72.72, 402.57) | 1546.22 (933.88, 2366.37) | 857.6108 | 91.41 (41.17, 227.91) | 411.95 (248.81, 630.45) | 5.012 (4.075, 5.958) |
| Malta | 0.91 (0.80, 1.04) | 2.97 (1.68, 4.70) | 224.7084 | 3.66 (3.22, 4.18) | 4.51 (2.55, 7.14) | 0.546 (-0.430, 1.532) |
| Marshall Islands | 0.21 (0.01, 1.25) | 2.06 (0.01, 18.66) | 900.414 | 27.35 (1.32, 165.77) | 189.27 (1.14, 1711.05) | 6.846 (6.471, 7.222) |
| Mauritania | 117.36 (39.71, 408.69) | 37.24 (8.82, 99.04) | -68.2647 | 223.79 (75.72, 779.35) | 36.99 (8.76, 98.38) | -6.027 (-6.529, -5.522) |
| Mauritius | 1.06 (0.95, 1.17) | 15.61 (13.70, 17.92) | 1378.422 | 3.12 (2.79, 3.46) | 17.25 (15.13, 19.79) | 6.708 (3.071, 10.474) |
| Mexico | 120.54 (105.11, 155.28) | 1552.64 (1412.73, 1702.58) | 1188.091 | 5.74 (5.01, 7.40) | 24.38 (22.18, 26.73) | 5.391 (-2.028, 13.372) |
| Micronesia (Federated States of) | 0.48 (0.04, 3.60) | 17.33 (0.07, 150.95) | 3475.754 | 23.70 (1.84, 175.94) | 764.59 (3.24, 6659.85) | 12.497 (11.441, 13.563) |
| Monaco | 0.64 (0.11, 2.48) | 2.51 (0.37, 9.10) | 291.9511 | 12.87 (2.13, 49.81) | 36.01 (5.27, 130.43) | 3.511 (3.236, 3.786) |
| Mongolia | 0.00 (0.00, 0.00) | 9.36 (0.58, 29.14) | 10673403 | 0.01 (0.02, 0.00) | 11.19 (0.69, 34.84) | 28.494 (25.787, 31.259) |
| Montenegro | 0.08 (0.06, 0.10) | 0.93 (0.72, 1.20) | 1066.526 | 0.25 (0.19, 0.32) | 1.61 (1.25, 2.07) | 7.083 (4.663, 9.559) |
| Morocco | 46.06 (7.09, 244.06) | 233.41 (11.62, 1497.27) | 406.7986 | 7.39 (1.14, 39.18) | 16.86 (0.84, 108.16) | 2.842 (2.407, 3.279) |
| Mozambique | 500.83 (278.10, 896.34) | 15881.82 (11284.50, 21829.27) | 3071.093 | 204.32 (113.46, 365.68) | 3541.80 (2516.55, 4868.14) | 9.975 (8.450, 11.522) |
| Myanmar | 21.42 (13.39, 35.50) | 1478.91 (798.46, 2719.48) | 6802.924 | 2.16 (1.35, 3.58) | 70.25 (37.93, 129.18) | 12.335 (11.309, 13.371) |
| Namibia | 104.45 (64.52, 164.79) | 2104.25 (1473.01, 2964.77) | 1914.519 | 277.70 (171.53, 438.10) | 3055.65 (2139.01, 4305.25) | 8.799 (7.287, 10.331) |
| Nauru | 0.00 (0.00, 0.01) | 0.09 (0.00, 0.44) | 2760.385 | 2.68 (0.41, 9.80) | 106.75 (1.46, 551.80) | 13.578 (13.300, 13.856) |
| Nepal | 0.02 (0.00, 0.10) | 964.34 (17.74, 8497.19) | 5343753 | 0.00 (0.00, 0.03) | 88.12 (1.62, 776.49) | 37.496 (34.726, 40.323) |
| Netherlands | 42.89 (37.10, 50.55) | 191.86 (112.57, 289.38) | 347.3248 | 3.34 (2.89, 3.93) | 8.24 (4.83, 12.42) | 3.260 (1.847, 4.694) |
| New Zealand | 12.91 (11.66, 14.53) | 12.49 (8.29, 17.70) | -3.27954 | 5.22 (4.71, 5.87) | 2.41 (1.60, 3.41) | -3.077 (-4.550, -1.581) |
| Nicaragua | 1.21 (1.03, 1.40) | 225.27 (65.40, 450.14) | 18583.03 | 1.65 (1.41, 1.91) | 104.68 (30.39, 209.17) | 15.704 (15.044, 16.367) |
| Niger | 65.72 (33.09, 128.99) | 494.68 (297.81, 803.97) | 652.6807 | 63.53 (31.99, 124.70) | 159.43 (95.98, 259.11) | 2.874 (1.763, 3.996) |
| Nigeria | 2335.08 (1542.20, 3575.94) | 23974.70 (20038.15, 28414.19) | 926.7191 | 114.09 (75.35, 174.72) | 678.97 (567.49, 804.70) | 6.126 (5.437, 6.821) |
| Niue | 0.00 (0.00, 0.01) | 0.10 (0.00, 0.49) | 3651.341 | 1.78 (0.25, 6.34) | 77.13 (1.00, 391.17) | 13.945 (13.438, 14.455) |
| North Macedonia | 0.03 (0.03, 0.04) | 0.72 (0.55, 0.93) | 2121.91 | 0.04 (0.03, 0.05) | 0.40 (0.31, 0.52) | 8.635 (4.617, 12.807) |
| Northern Mariana Islands | 0.01 (0.00, 0.02) | 0.32 (0.03, 1.03) | 3527.26 | 1.80 (0.96, 3.23) | 20.15 (2.10, 64.94) | 8.439 (7.981, 8.898) |
| Norway | 10.57 (9.68, 11.88) | 17.34 (11.70, 24.59) | 64.0506 | 2.16 (1.98, 2.43) | 2.66 (1.79, 3.77) | 0.583 (-1.554, 2.766) |
| Oman | 0.42 (0.34, 0.52) | 25.50 (14.34, 45.51) | 5915.596 | 1.84 (1.46, 2.25) | 47.33 (26.62, 84.46) | 12.005 (10.773, 13.250) |
| Pakistan | 236.51 (34.51, 1042.10) | 1361.36 (24.62, 8912.33) | 475.6124 | 8.02 (1.17, 35.35) | 30.12 (0.54, 197.17) | 4.703 (4.192, 5.217) |
| Palau | 0.01 (0.00, 0.05) | 0.78 (0.01, 4.10) | 6028.435 | 2.67 (0.34, 10.21) | 96.99 (1.36, 508.02) | 13.165 (12.810, 13.521) |
| Palestine | 0.01 (0.01, 0.01) | 0.28 (0.21, 0.39) | 2459.563 | 0.03 (0.02, 0.03) | 0.29 (0.22, 0.41) | 8.843 (8.230, 9.460) |
| Panama | 44.59 (38.99, 51.49) | 181.61 (160.30, 201.29) | 307.2659 | 54.60 (47.74, 63.05) | 74.18 (65.48, 82.22) | 0.942 (-0.059, 1.953) |
| Papua New Guinea | 0.41 (0.11, 0.97) | 970.34 (342.13, 2834.98) | 237798.3 | 0.62 (0.17, 1.48) | 591.02 (208.39, 1726.75) | 26.500 (24.287, 28.753) |
| Paraguay | 4.39 (2.57, 6.30) | 224.41 (89.67, 425.33) | 5008.289 | 3.78 (2.21, 5.42) | 77.36 (30.91, 146.62) | 11.625 (10.105, 13.165) |
| Peru | 183.54 (146.34, 232.87) | 1121.34 (599.67, 1954.28) | 510.9476 | 30.70 (24.48, 38.96) | 60.73 (32.48, 105.84) | 2.490 (1.774, 3.210) |
| Philippines | 2.17 (2.12, 2.22) | 279.87 (111.44, 654.12) | 12816.65 | 0.17 (0.16, 0.17) | 8.31 (3.31, 19.43) | 14.318 (13.832, 14.806) |
| Poland | 1.20 (1.13, 1.33) | 46.07 (31.24, 66.03) | 3730.929 | 0.05 (0.05, 0.06) | 1.05 (0.71, 1.50) | 11.341 (8.539, 14.216) |
| Portugal | 62.95 (53.85, 81.00) | 366.05 (321.14, 419.48) | 481.5195 | 7.30 (6.24, 9.39) | 21.47 (18.84, 24.60) | 3.139 (-1.464, 7.956) |
| Puerto Rico | 105.21 (92.38, 130.50) | 175.01 (151.59, 186.79) | 66.3493 | 46.77 (41.07, 58.01) | 34.50 (29.89, 36.83) | -0.720 (-3.316, 1.945) |
| Qatar | 0.00 (0.00, 0.00) | 0.54 (0.41, 0.73) | 54671.08 | 0.04 (0.03, 0.05) | 3.55 (2.72, 4.80) | 16.569 (15.107, 18.051) |
| Republic of Korea | 28.98 (20.73, 35.86) | 782.61 (416.24, 1479.93) | 2600.708 | 2.28 (1.63, 2.82) | 14.90 (7.92, 28.17) | 6.469 (4.230, 8.755) |
| Republic of Moldova | 0.33 (0.23, 0.56) | 6.31 (4.14, 8.96) | 1838.377 | 0.15 (0.11, 0.27) | 1.96 (1.29, 2.78) | 9.068 (7.659, 10.495) |
| Romania | 12.46 (10.91, 14.33) | 79.71 (55.30, 116.18) | 539.6108 | 0.87 (0.76, 1.00) | 3.25 (2.26, 4.74) | 4.901 (2.443, 7.418) |
| Russian Federation | 37.85 (34.47, 44.66) | 403.42 (339.31, 475.20) | 965.9237 | 0.39 (0.36, 0.46) | 2.96 (2.49, 3.49) | 6.937 (4.849, 9.067) |
| Rwanda | 500.67 (208.93, 1385.51) | 1421.83 (962.28, 2072.67) | 183.9879 | 408.27 (170.37, 1129.81) | 608.15 (411.59, 886.53) | 1.018 (0.301, 1.741) |
| Saint Kitts and Nevis | 1.26 (0.09, 4.15) | 19.07 (2.04, 68.12) | 1408.305 | 47.29 (3.41, 155.30) | 713.71 (76.21, 2549.83) | 9.759 (9.389, 10.132) |
| Saint Lucia | 3.47 (3.25, 3.81) | 3.81 (3.28, 4.33) | 9.65055 | 65.93 (61.75, 72.33) | 30.94 (26.69, 35.22) | -1.924 (-3.267, -0.561) |
| Saint Vincent and the Grenadines | 7.82 (7.31, 9.04) | 9.20 (8.60, 9.71) | 17.65028 | 177.02 (165.41, 204.57) | 122.56 (114.56, 129.34) | -1.186 (-3.620, 1.309) |
| Samoa | 0.93 (0.06, 5.74) | 9.59 (0.11, 87.76) | 933.9065 | 22.48 (1.57, 139.17) | 141.46 (1.62, 1294.82) | 6.578 (5.859, 7.302) |
| San Marino | 0.33 (0.04, 1.34) | 1.54 (0.22, 5.47) | 363.1817 | 15.42 (1.88, 62.31) | 34.63 (4.95, 123.19) | 2.678 (2.313, 3.043) |
| Sao Tome and Principe | 0.62 (0.41, 0.86) | 0.35 (0.21, 0.55) | -43.1378 | 18.72 (12.21, 25.95) | 7.93 (4.62, 12.28) | -2.998 (-3.530, -2.463) |
| Saudi Arabia | 25.74 (7.78, 49.93) | 145.28 (52.26, 500.57) | 464.4571 | 10.47 (3.17, 20.32) | 29.86 (10.74, 102.89) | 3.551 (3.294, 3.809) |
| Senegal | 75.58 (43.85, 131.45) | 641.81 (405.95, 974.69) | 749.1417 | 49.98 (29.00, 86.92) | 185.44 (117.29, 281.62) | 4.417 (3.554, 5.287) |
| Serbia | 5.86 (0.00, 10.24) | 34.77 (18.53, 91.99) | 493.7279 | 1.11 (0.00, 1.94) | 3.38 (1.80, 8.94) | 4.269 (3.306, 5.241) |
| Seychelles | 0.08 (0.06, 0.11) | 2.38 (1.65, 3.25) | 3032.398 | 2.21 (1.70, 3.11) | 45.33 (31.50, 61.98) | 11.107 (10.292, 11.929) |
| Sierra Leone | 45.95 (20.57, 112.55) | 854.91 (554.52, 1301.42) | 1760.571 | 44.96 (20.12, 110.12) | 517.26 (335.51, 787.41) | 8.454 (7.668, 9.246) |
| Singapore | 4.01 (3.29, 5.08) | 69.78 (46.03, 113.55) | 1641.9 | 3.83 (3.14, 4.85) | 16.70 (11.01, 27.17) | 5.436 (4.629, 6.249) |
| Slovakia | 0.04 (0.00, 0.06) | 2.92 (2.20, 4.04) | 6973.837 | 0.01 (0.00, 0.02) | 0.52 (0.39, 0.72) | 13.316 (8.216, 18.658) |
| Slovenia | 0.02 (0.02, 0.03) | 1.09 (0.50, 1.99) | 4316.18 | 0.02 (0.01, 0.02) | 0.38 (0.18, 0.69) | 11.008 (10.326, 11.694) |
| Solomon Islands | 1.11 (0.07, 6.97) | 17.76 (0.18, 166.73) | 1504.942 | 26.05 (1.55, 164.05) | 172.69 (1.79, 1620.84) | 6.751 (6.227, 7.277) |
| Somalia | 3.10 (0.94, 9.10) | 584.86 (324.84, 991.50) | 18784.25 | 3.81 (1.16, 11.20) | 257.06 (142.77, 435.79) | 15.018 (14.111, 15.933) |
| South Africa | 709.28 (427.37, 1130.39) | 68155.74 (57761.87, 80909.38) | 9509.108 | 68.89 (41.51, 109.79) | 3155.33 (2674.13, 3745.77) | 13.967 (12.280, 15.681) |
| South Sudan | 67.65 (25.76, 195.40) | 1156.80 (420.48, 2538.53) | 1609.907 | 60.16 (22.91, 173.76) | 752.14 (273.39, 1650.54) | 9.096 (8.565, 9.630) |
| Spain | 109.08 (90.89, 135.34) | 388.28 (313.83, 484.98) | 255.9529 | 3.17 (2.64, 3.93) | 5.86 (4.74, 7.32) | 1.579 (-1.158, 4.392) |
| Sri Lanka | 1.03 (0.54, 1.75) | 11.39 (6.44, 19.51) | 1001.86 | 0.21 (0.11, 0.35) | 0.84 (0.47, 1.43) | 5.033 (3.876, 6.204) |
| Sudan | 63.27 (13.05, 229.63) | 1553.06 (659.93, 3650.41) | 2354.821 | 13.44 (2.77, 48.79) | 185.22 (78.71, 435.36) | 9.299 (8.402, 10.204) |
| Suriname | 21.84 (20.32, 24.79) | 36.43 (33.67, 38.89) | 66.79683 | 169.41 (157.64, 192.31) | 115.64 (106.87, 123.43) | -0.419 (-1.834, 1.016) |
| Sweden | 22.34 (19.95, 26.15) | 38.39 (22.11, 64.04) | 71.8387 | 2.04 (1.83, 2.39) | 2.50 (1.44, 4.16) | 0.396 (-0.625, 1.427) |
| Switzerland | 15.37 (6.89, 30.07) | 189.48 (115.62, 291.42) | 1132.661 | 2.20 (0.99, 4.31) | 15.85 (9.67, 24.37) | 8.809 (6.959, 10.691) |
| Syrian Arab Republic | 0.26 (0.21, 0.34) | 1.57 (0.91, 2.95) | 502.6427 | 0.12 (0.09, 0.15) | 0.29 (0.17, 0.55) | 2.989 (1.805, 4.187) |
| Taiwan (Province of China) | 3.16 (2.50, 3.98) | 130.50 (109.04, 155.93) | 4026.408 | 0.43 (0.34, 0.54) | 5.83 (4.87, 6.96) | 9.427 (5.931, 13.038) |
| Tajikistan | 0.02 (0.02, 0.03) | 2.60 (1.23, 5.79) | 11619.42 | 0.02 (0.01, 0.02) | 1.81 (0.86, 4.03) | 17.473 (13.955, 21.099) |
| Thailand | 168.23 (124.33, 263.50) | 17446.72 (10183.69, 33771.64) | 10270.66 | 11.00 (8.13, 17.23) | 308.42 (180.03, 597.01) | 12.356 (10.755, 13.980) |
| Timor-Leste | 3.78 (0.07, 29.57) | 216.71 (1.27, 2028.24) | 5631.74 | 43.74 (0.83, 342.12) | 548.37 (3.22, 5132.27) | 9.111 (8.433, 9.792) |
| Togo | 61.32 (27.61, 141.36) | 1007.11 (627.15, 1583.82) | 1542.412 | 119.13 (53.64, 274.62) | 716.47 (446.16, 1126.75) | 6.049 (4.445, 7.677) |
| Tokelau | 0.00 (0.00, 0.01) | 0.05 (0.00, 0.29) | 2230.384 | 2.70 (0.42, 10.03) | 84.55 (1.20, 455.44) | 12.602 (12.242, 12.963) |
| Tonga | 0.12 (0.07, 0.22) | 1.68 (0.12, 6.15) | 1278.499 | 4.65 (2.59, 8.26) | 38.04 (2.74, 139.09) | 7.559 (7.103, 8.017) |
| Trinidad and Tobago | 14.44 (13.06, 16.71) | 48.33 (40.81, 56.03) | 234.736 | 30.59 (27.67, 35.41) | 46.30 (39.10, 53.67) | 1.193 (0.376, 2.017) |
| Tunisia | 3.45 (0.34, 17.33) | 89.37 (2.44, 501.33) | 2488.514 | 1.42 (0.14, 7.12) | 13.62 (0.37, 76.43) | 8.143 (7.661, 8.628) |
| Turkey | 4.91 (0.00, 8.02) | 213.92 (166.61, 270.81) | 4253.767 | 0.31 (0.00, 0.51) | 4.47 (3.49, 5.66) | 9.488 (8.843, 10.137) |
| Turkmenistan | 0.02 (0.01, 0.02) | 0.66 (0.39, 0.91) | 4060.744 | 0.02 (0.02, 0.02) | 0.43 (0.26, 0.61) | 11.429 (10.575, 12.289) |
| Tuvalu | 0.01 (0.00, 0.02) | 0.45 (0.01, 2.44) | 6031.652 | 2.51 (0.41, 8.42) | 89.18 (1.40, 487.08) | 13.109 (12.852, 13.366) |
| Uganda | 20865.68 (13534.18, 29501.60) | 6368.10 (4438.55, 8868.75) | -69.4805 | 7053.75 (4575.30, 9973.17) | 1068.12 (744.48, 1487.55) | -6.373 (-6.797, -5.947) |
| Ukraine | 7.73 (6.88, 8.73) | 82.99 (56.95, 101.50) | 973.6139 | 0.19 (0.17, 0.22) | 1.76 (1.21, 2.15) | 7.960 (6.555, 9.383) |
| United Arab Emirates | 0.26 (0.02, 1.60) | 619.74 (4.16, 4481.39) | 239054 | 2.74 (0.22, 16.95) | 1537.07 (10.32, 11114.65) | 24.299 (21.689, 26.964) |
| United Kingdom | 93.35 (81.86, 114.34) | 710.13 (392.94, 1083.22) | 660.7443 | 1.50 (1.32, 1.84) | 8.09 (4.48, 12.34) | 5.652 (4.491, 6.826) |
| United Republic of Tanzania | 10616.64 (5489.40, 17685.83) | 10294.32 (6354.43, 16038.30) | -3.03595 | 2157.54 (1115.57, 3594.16) | 939.83 (580.13, 1464.23) | -2.883 (-3.885, -1.872) |
| United States of America | 3480.46 (3142.60, 4011.31) | 11884.10 (7527.48, 18267.39) | 241.4523 | 16.31 (14.73, 18.80) | 33.05 (20.94, 50.81) | 2.077 (1.051, 3.114) |
| United States Virgin Islands | 2.28 (2.05, 2.68) | 8.29 (7.60, 8.88) | 263.5561 | 56.03 (50.35, 65.87) | 67.15 (61.55, 71.94) | 0.640 (-0.889, 2.192) |
| Uruguay | 137.59 (116.61, 182.14) | 298.35 (275.68, 331.02) | 116.8436 | 56.02 (47.48, 74.16) | 80.85 (74.70, 89.70) | 1.148 (0.685, 1.614) |
| Uzbekistan | 2.42 (1.97, 3.55) | 7.82 (5.55, 10.97) | 223.9221 | 0.45 (0.37, 0.66) | 1.43 (1.01, 2.00) | 3.983 (2.681, 5.302) |
| Vanuatu | 0.72 (0.05, 3.88) | 16.98 (0.25, 149.74) | 2243.013 | 26.02 (1.93, 139.15) | 213.57 (3.10, 1883.59) | 7.435 (6.443, 8.436) |
| Venezuela (Bolivarian Republic of) | 93.66 (79.88, 119.10) | 753.37 (656.50, 862.07) | 704.3498 | 19.70 (16.80, 25.06) | 49.26 (42.93, 56.37) | 3.406 (2.903, 3.912) |
| Viet Nam | 69.55 (49.40, 97.79) | 190.15 (123.73, 291.51) | 173.396 | 3.41 (2.42, 4.80) | 4.61 (3.00, 7.07) | 1.093 (0.289, 1.904) |
| Yemen | 20.92 (2.02, 121.86) | 97.84 (2.92, 610.78) | 367.6731 | 10.76 (1.04, 62.69) | 17.31 (0.52, 108.03) | 1.616 (1.288, 1.945) |
| Zambia | 2679.43 (1300.37, 4704.85) | 7086.83 (4723.51, 10222.71) | 164.4905 | 2305.57 (1118.93, 4048.39) | 2576.81 (1717.49, 3717.03) | 0.177 (-0.867, 1.232) |
| Zimbabwe | 2874.26 (1094.69, 7022.05) | 8869.65 (5953.16, 13277.82) | 208.5893 | 1601.69 (610.02, 3913.06) | 3064.38 (2056.76, 4587.36) | 1.963 (1.163, 2.770) |

Note: AAPC, average annual percentage change; CI, confidence interval; DALYs: disability-adjusted life years; UI, uncertainty interval


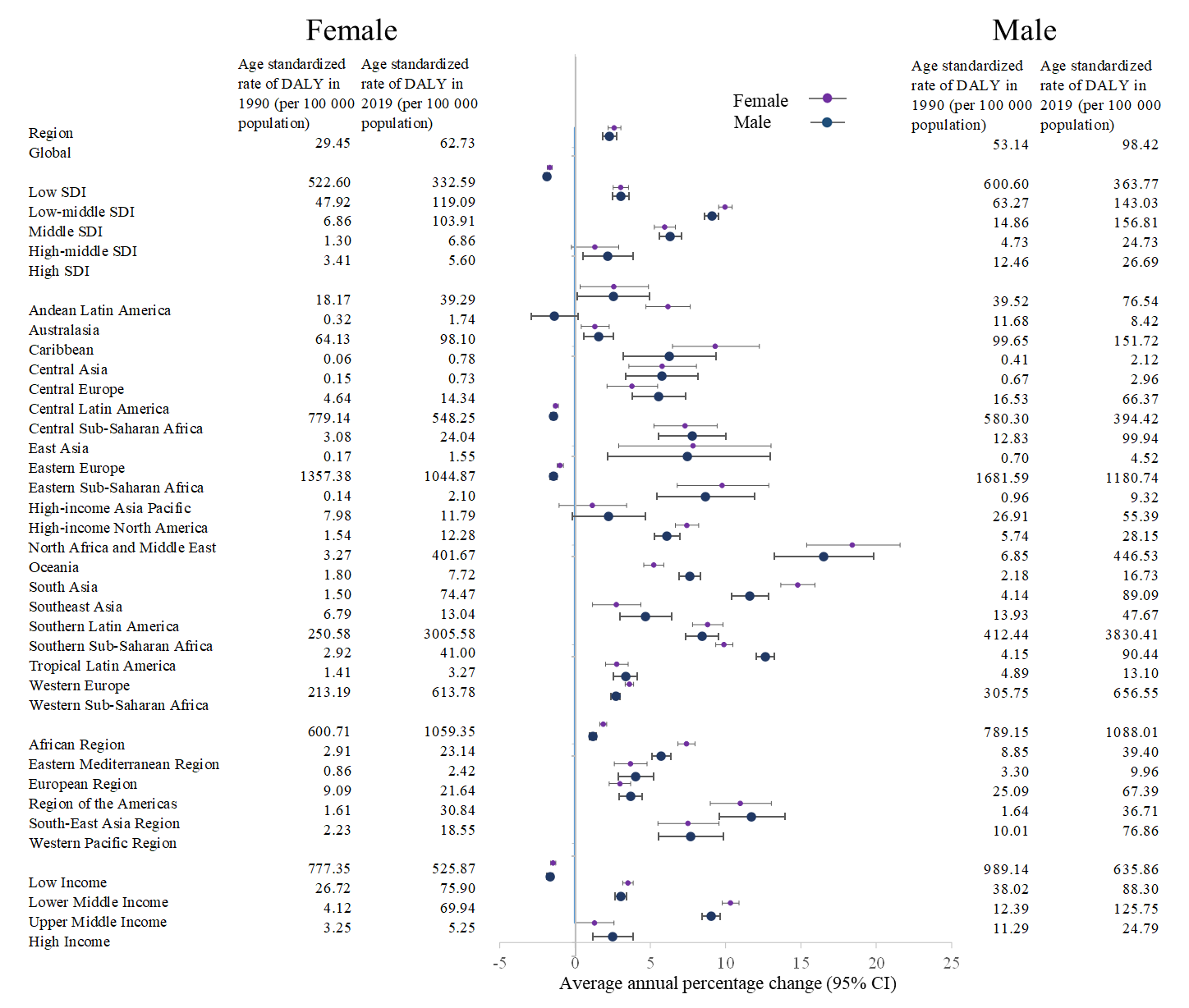


**Supplementary Figure S7**. The DALY rate and its trend of HIV/AIDs from 1990 to 2019 in global, SDI, GBD, WHO and World Bank income regions by sex.

CI: confidence interval; GBD: Global Burden of Diseases, Injuries, and Risk Factors Study; SDI: Sociodemographic Index; WHO: World Health Organization

**Supplementary Table S17**. The HIV/AIDs DALY rate and its trends among older adults by region and sex level from 1990 to 2019.

|  | Female |  |  | Male |  |  |
| --- | --- | --- | --- | --- | --- | --- |
|  | 1990 (95% UI) | 2019 (95% UI) | AAPC (95% CI) | 1990 (95% UI) | 2019 (95% UI) | AAPC (95% CI) |
| **Global** | 29.45 (20.99, 39.74) | 62.73 (53.09, 75.99) | 2.619 (2.245, 2.995) | 53.14 (37.31, 73.32) | 98.42 (84.39, 118.01) | 2.110 (1.474, 2.751) |
| **SDI region** | | | | | | |
| Low SDI | 522.60 (362.49, 722.98) | 332.59 (268.29, 434.83) | -1.668 (-2.127, -1.207) | 600.60 (389.17, 873.73) | 363.77 (280.19, 483.35) | -1.805 (-2.136, -1.472) |
| Low-middle SDI | 47.92 (29.21, 75.29) | 119.09 (95.92, 153.80) | 3.032 (2.618, 3.447) | 63.27 (37.66, 103.78) | 143.03 (115.65, 185.57) | 2.789 (2.088, 3.495) |
| Middle SDI | 6.86 (5.21, 8.96) | 103.91 (87.39, 125.02) | 9.975 (8.793, 11.170) | 14.86 (10.22, 19.32) | 156.81 (133.59, 185.14) | 8.466 (8.133, 8.799) |
| High-middle SDI | 1.30 (1.11, 1.52) | 6.86 (5.59, 8.33) | 5.963 (5.103, 6.829) | 4.73 (3.70, 5.95) | 24.73 (20.48, 30.13) | 5.888 (5.344, 6.435) |
| High SDI | 3.41 (3.08, 3.97) | 5.60 (3.53, 8.20) | 1.318 (0.097, 2.553) | 12.46 (11.21, 14.42) | 26.69 (17.58, 40.04) | 2.013 (0.731, 3.312) |
| **GBD region** | | | | | | |
| Andean Latin America | 18.17 (9.19, 35.58) | 39.29 (14.25, 105.45) | 2.579 (1.393, 3.780) | 39.52 (24.93, 80.63) | 76.54 (50.58, 148.27) | 2.327 (1.510, 3.149) |
| Australasia | 0.32 (0.25, 0.44) | 1.74 (0.79, 3.14) | 6.178 (4.761, 7.615) | 11.68 (10.56, 13.28) | 8.42 (5.69, 11.98) | -1.323 (-2.447, -0.186) |
| Caribbean | 64.13 (39.13, 114.36) | 98.10 (68.23, 139.50) | 1.331 (0.546, 2.123) | 99.65 (61.30, 178.41) | 151.72 (107.56, 214.76) | 1.410 (0.245, 2.588) |
| Central Asia | 0.06 (0.05, 0.07) | 0.78 (0.53, 1.13) | 9.314 (7.677, 10.976) | 0.41 (0.33, 0.58) | 2.12 (1.22, 3.58) | 5.822 (5.252, 6.396) |
| Central Europe | 0.15 (0.11, 0.18) | 0.73 (0.47, 1.06) | 5.792 (4.155, 7.455) | 0.67 (0.49, 0.80) | 2.96 (2.20, 3.95) | 5.347 (4.496, 6.206) |
| Central Latin America | 4.64 (4.16, 5.61) | 14.34 (11.10, 18.94) | 3.796 (3.483, 4.111) | 16.53 (14.85, 19.52) | 66.37 (54.68, 81.40) | 5.173 (3.722, 6.643) |
| Central Sub-Saharan Africa | 779.14 (502.96, 1156.17) | 548.25 (411.70, 734.40) | -1.273 (-1.678, -0.867) | 580.30 (346.72, 915.44) | 394.42 (289.78, 537.05) | -1.385 (-1.687, -1.082) |
| East Asia | 3.08 (1.79, 4.17) | 24.04 (15.86, 33.84) | 7.313 (5.901, 8.743) | 12.83 (6.87, 17.31) | 99.94 (73.13, 128.65) | 7.237 (6.088, 8.398) |
| Eastern Europe | 0.17 (0.16, 0.20) | 1.55 (1.25, 1.90) | 7.839 (6.346, 9.353) | 0.70 (0.63, 0.82) | 4.52 (3.73, 5.17) | 6.942 (6.214, 7.675) |
| Eastern Sub-Saharan Africa | 1357.38 (915.95, 1866.99) | 1044.87 (825.04, 1371.82) | -0.983 (-1.477, -0.488) | 1681.59 (1064.22, 2443.39) | 1180.74 (922.31, 1552.50) | -1.375 (-1.934, -0.813) |
| High-income Asia Pacific | 0.14 (0.12, 0.18) | 2.10 (1.04, 3.61) | 9.773 (8.574, 10.984) | 0.96 (0.77, 1.18) | 9.32 (5.26, 16.03) | 8.051 (7.056, 9.056) |
| High-income North America | 7.98 (7.20, 9.28) | 11.79 (7.51, 17.73) | 1.156 (-0.308, 2.641) | 26.91 (24.21, 31.10) | 55.39 (35.16, 84.47) | 2.038 (0.824, 3.266) |
| North Africa and Middle East | 1.54 (0.71, 4.23) | 12.28 (7.10, 23.12) | 7.429 (6.648, 8.216) | 5.74 (1.80, 23.12) | 28.15 (9.59, 102.36) | 5.693 (5.268, 6.120) |
| Oceania | 3.27 (0.89, 15.98) | 401.67 (149.43, 1146.45) | 18.425 (17.246, 19.616) | 6.85 (1.86, 29.61) | 446.53 (139.59, 1454.07) | 15.438 (14.345, 16.541) |
| South Asia | 1.80 (0.92, 4.33) | 7.72 (3.72, 24.13) | 5.229 (4.065, 6.406) | 2.18 (0.66, 7.91) | 16.73 (7.63, 48.69) | 7.094 (6.565, 7.626) |
| Southeast Asia | 1.50 (1.05, 2.26) | 74.47 (40.86, 158.09) | 14.789 (13.603, 15.987) | 4.14 (3.07, 6.18) | 89.09 (68.28, 122.75) | 10.854 (9.914, 11.801) |
| Southern Latin America | 6.79 (5.95, 8.29) | 13.04 (9.84, 18.36) | 2.754 (2.215, 3.295) | 13.93 (12.21, 16.64) | 47.67 (41.52, 56.63) | 4.342 (2.114, 6.618) |
| Southern Sub-Saharan Africa | 250.58 (122.17, 506.18) | 3005.58 (2439.52, 3629.12) | 8.805 (7.682, 9.939) | 412.44 (175.58, 971.61) | 3830.41 (3065.96, 4940.65) | 7.870 (6.272, 9.491) |
| Tropical Latin America | 2.92 (2.53, 3.69) | 41.00 (34.05, 49.79) | 9.900 (3.303, 16.918) | 4.15 (3.37, 5.98) | 90.44 (76.52, 107.59) | 11.805 (6.835, 17.006) |
| Western Europe | 1.41 (1.24, 1.72) | 3.27 (2.15, 4.68) | 2.779 (2.018, 3.546) | 4.89 (4.22, 6.01) | 13.10 (9.81, 17.08) | 3.095 (1.410, 4.807) |
| Western Sub-Saharan Africa | 213.19 (137.78, 318.44) | 613.78 (500.16, 766.17) | 3.613 (3.067, 4.163) | 305.75 (188.79, 477.92) | 656.55 (533.03, 815.98) | 2.487 (2.007, 2.969) |
| **WHO region** | | | | | | |
| African Region | 600.71 (410.52, 832.40) | 1059.35 (888.75, 1314.60) | 1.881 (1.602, 2.161) | 789.15 (511.57, 1125.25) | 1088.01 (922.06, 1319.48) | 1.068 (0.411, 1.728) |
| Eastern Mediterranean Region | 2.91 (1.08, 8.27) | 23.14 (12.69, 59.39) | 7.406 (6.937, 7.878) | 8.85 (2.29, 31.42) | 39.40 (11.69, 154.17) | 5.335 (4.924, 5.747) |
| European Region | 0.86 (0.76, 1.04) | 2.42 (1.73, 3.27) | 3.689 (2.916, 4.468) | 3.30 (2.88, 3.99) | 9.96 (7.67, 12.70) | 3.740 (2.216, 5.287) |
| Region of the Americas | 9.09 (7.86, 11.07) | 21.64 (17.45, 27.23) | 2.982 (2.591, 3.374) | 25.09 (22.05, 29.60) | 67.39 (54.01, 85.01) | 3.397 (2.820, 3.977) |
| South-East Asia Region | 1.61 (1.06, 3.07) | 30.84 (19.00, 60.76) | 10.978 (9.840, 12.127) | 1.64 (1.18, 2.65) | 36.71 (27.58, 59.88) | 10.960 (10.465, 11.457) |
| Western Pacific Region | 2.23 (1.34, 2.92) | 18.55 (12.57, 25.47) | 7.506 (6.789, 8.229) | 10.01 (5.72, 13.28) | 76.86 (56.80, 98.76) | 7.156 (6.285, 8.034) |
| Low Income | 777.35 (534.04, 1071.30) | 525.87 (419.15, 684.35) | -1.451 (-1.777, -1.124) | 989.14 (631.08, 1437.68) | 635.86 (493.03, 842.23) | -1.610 (-2.017, -1.201) |
| Lower Middle Income | 26.72 (17.12, 40.72) | 75.90 (61.08, 97.92) | 3.517 (2.720, 4.320) | 38.02 (23.16, 61.24) | 88.30 (70.34, 115.15) | 2.826 (2.163, 3.494) |
| Upper Middle Income | 4.12 (3.09, 5.22) | 69.94 (58.55, 83.45) | 10.331 (9.959, 10.704) | 12.39 (8.20, 15.89) | 125.75 (106.33, 149.06) | 8.438 (8.060, 8.816) |
| High Income | 3.25 (2.92, 3.79) | 5.25 (3.46, 7.53) | 1.299 (0.208, 2.402) | 11.29 (10.14, 13.16) | 24.79 (16.89, 36.56) | 2.314 (1.326, 3.311) |

Note: AAPC, average annual percentage change; CI, confidence interval; DALYs: disability-adjusted life years; UI, uncertainty interval

**Supplementary Table S18**. The HIV/AIDs DALYs and its changes among older adults by region and sex level in 1990 and 2019.

|  | Female |  |  | Male |  |  |
| --- | --- | --- | --- | --- | --- | --- |
|  | 1990 (95% UI) | 2019 (95% UI) | Percentage change (%) | 1990 (95% UI) | 2019 (95% UI) | Percentage change (%) |
| **Global** | 35135.66 (25042.50, 47414.38) | 163266.42 (138164.12, 197765.68) | 364.67 | 43699.49 (30677.32, 60289.55) | 200196.86 (171655.96, 240050.22) | 358.12 |
| **SDI region** | | | | | | |
| Low SDI | 25291.86 (17543.09, 34989.54) | 39197.57 (31619.29, 51246.12) | 54.98 | 28897.71 (18725.03, 42039.28) | 39111.91 (30125.32, 51968.76) | 35.35 |
| Low-middle SDI | 6215.80 (3788.95, 9766.07) | 43871.93 (35335.49, 56656.13) | 605.81 | 7749.82 (4612.74, 12710.96) | 44946.17 (36343.32, 58312.61) | 479.96 |
| Middle SDI | 1731.73 (1315.10, 2263.47) | 71180.91 (59867.16, 85639.08) | 4010.40 | 2970.01 (2042.57, 3861.50) | 89117.71 (75919.43, 105217.01) | 2900.59 |
| High-middle SDI | 455.02 (387.28, 530.49) | 4793.33 (3907.94, 5822.59) | 953.44 | 970.71 (758.79, 1220.66) | 12139.46 (10053.46, 14789.56) | 1150.58 |
| High SDI | 1407.72 (1269.52, 1638.98) | 4092.04 (2584.84, 5993.55) | 190.69 | 3067.45 (2758.81, 3549.40) | 14734.14 (9704.63, 22108.85) | 380.34 |
| **GBD region** | | | | | | |
| Andean Latin America | 97.38 (49.24, 190.75) | 651.52 (236.30, 1748.70) | 569.08 | 191.60 (120.85, 390.89) | 1135.88 (750.54, 2200.20) | 492.85 |
| Australasia | 2.80 (2.16, 3.84) | 31.37 (14.26, 56.51) | 1021.28 | 68.33 (61.78, 77.72) | 127.54 (86.18, 181.54) | 86.65 |
| Caribbean | 507.35 (309.54, 904.72) | 1659.04 (1153.88, 2359.26) | 227.00 | 696.71 (428.60, 1247.37) | 2110.29 (1496.00, 2987.02) | 202.89 |
| Central Asia | 0.94 (0.83, 1.06) | 13.74 (9.35, 19.81) | 1366.19 | 2.94 (2.33, 4.14) | 22.07 (12.71, 37.29) | 651.57 |
| Central Europe | 7.17 (5.14, 8.88) | 63.12 (40.73, 91.55) | 780.19 | 19.86 (14.36, 23.68) | 158.86 (118.04, 211.91) | 699.76 |
| Central Latin America | 99.78 (89.45, 120.55) | 1030.04 (797.57, 1360.69) | 932.36 | 316.58 (284.50, 373.90) | 3877.75 (3194.58, 4755.38) | 1124.88 |
| Central Sub-Saharan Africa | 3405.72 (2198.49, 5053.80) | 6523.29 (4898.57, 8738.16) | 91.54 | 2319.27 (1385.73, 3658.71) | 3003.49 (2206.66, 4089.61) | 29.50 |
| East Asia | 694.67 (403.61, 940.01) | 14715.73 (9708.69, 20714.66) | 2018.37 | 2189.09 (1172.29, 2952.89) | 50777.37 (37156.20, 65360.98) | 2219.56 |
| Eastern Europe | 19.42 (17.93, 22.27) | 219.45 (177.13, 268.62) | 1029.76 | 27.28 (24.69, 32.07) | 284.95 (235.20, 326.10) | 944.66 |
| Eastern Sub-Saharan Africa | 21674.09 (14625.60, 29811.47) | 38904.73 (30719.29, 51078.36) | 79.50 | 26110.92 (16524.76, 37939.92) | 36315.46 (28367.06, 47749.52) | 39.08 |
| High-income Asia Pacific | 9.93 (8.21, 12.42) | 404.34 (199.98, 695.22) | 3971.22 | 41.49 (33.35, 50.98) | 1303.44 (735.03, 2242.21) | 3041.60 |
| High-income North America | 1149.30 (1037.00, 1337.46) | 2696.79 (1718.23, 4054.40) | 134.65 | 2407.15 (2165.91, 2781.66) | 9739.20 (6181.14, 14852.50) | 304.60 |
| North Africa and Middle East | 58.81 (27.14, 161.64) | 1201.85 (695.30, 2262.72) | 1943.70 | 210.26 (65.77, 846.42) | 2738.88 (933.23, 9959.92) | 1202.63 |
| Oceania | 1.79 (0.49, 8.74) | 527.20 (196.13, 1504.74) | 29391.06 | 3.70 (1.01, 16.00) | 570.69 (178.41, 1858.38) | 15307.21 |
| South Asia | 192.38 (98.35, 462.75) | 2786.32 (1342.17, 8710.10) | 1348.37 | 244.03 (73.79, 886.20) | 5524.49 (2519.40, 16080.82) | 2163.89 |
| Southeast Asia | 92.01 (64.53, 139.03) | 12040.95 (6607.44, 25561.55) | 12987.25 | 198.26 (146.82, 295.81) | 10340.91 (7924.87, 14247.82) | 5115.80 |
| Southern Latin America | 105.75 (92.68, 129.20) | 404.13 (305.05, 568.83) | 282.16 | 149.47 (131.05, 178.53) | 1016.54 (885.28, 1207.41) | 580.08 |
| Southern Sub-Saharan Africa | 2019.10 (984.39, 4078.57) | 50119.60 (40680.19, 60517.43) | 2382.27 | 2164.73 (921.58, 5099.60) | 37682.23 (30161.84, 48604.39) | 1640.74 |
| Tropical Latin America | 70.28 (61.01, 88.90) | 3161.41 (2625.49, 3839.64) | 4398.57 | 80.39 (65.25, 115.87) | 5125.65 (4336.52, 6097.71) | 6275.80 |
| Western Europe | 334.62 (294.16, 407.16) | 1192.53 (784.51, 1706.25) | 256.39 | 667.28 (575.78, 820.30) | 3563.97 (2668.99, 4646.14) | 434.10 |
| Western Sub-Saharan Africa | 4592.38 (2967.98, 6859.68) | 24919.27 (20306.29, 31106.07) | 442.62 | 5590.14 (3451.74, 8738.00) | 24777.21 (20115.74, 30793.76) | 343.23 |
| **WHO region** | | | | | | |
| African Region | 31674.63 (21646.43, 43891.60) | 119967.60 (100647.77, 148873.77) | 278.75 | 36169.73 (23447.03, 51574.24) | 101717.43 (86202.95, 123357.48) | 181.22 |
| Eastern Mediterranean Region | 114.56 (42.35, 325.88) | 2005.64 (1099.73, 5148.04) | 1650.69 | 383.63 (99.34, 1361.88) | 3595.65 (1067.00, 14068.87) | 837.27 |
| European Region | 362.51 (321.75, 437.11) | 1536.28 (1100.13, 2078.89) | 323.79 | 721.08 (629.15, 874.07) | 4182.84 (3221.36, 5335.22) | 480.08 |
| Region of the Americas | 1972.53 (1705.54, 2401.03) | 9491.54 (7651.53, 11940.53) | 381.19 | 3744.01 (3290.73, 4418.27) | 22795.59 (18269.01, 28754.39) | 508.86 |
| South-East Asia Region | 219.92 (144.76, 417.62) | 14035.75 (8648.01, 27650.43) | 6282.19 | 209.79 (151.61, 339.08) | 14340.58 (10774.46, 23392.38) | 6735.83 |
| Western Pacific Region | 716.95 (429.81, 940.85) | 16031.07 (10857.61, 22009.13) | 2136.00 | 2343.19 (1337.75, 3107.32) | 53224.66 (39330.48, 68393.51) | 2171.46 |
| **World Bank Income region** | | | | | | |
| Low Income | 25503.95 (17521.31, 35148.06) | 40517.13 (32294.98, 52727.75) | 58.87 | 29055.60 (18537.74, 42231.37) | 37614.29 (29165.13, 49822.34) | 29.46 |
| Lower Middle Income | 6220.62 (3985.34, 9480.32) | 45735.30 (36803.69, 59005.89) | 635.22 | 7586.52 (4622.05, 12219.77) | 45102.33 (35928.10, 58819.51) | 494.51 |
| Upper Middle Income | 1742.33 (1305.80, 2207.03) | 72213.83 (60454.44, 86160.93) | 4044.67 | 3595.79 (2380.31, 4612.45) | 100965.22 (85369.13, 119679.28) | 2707.88 |
| High Income | 1635.23 (1469.53, 1911.58) | 4669.17 (3074.82, 6696.64) | 185.54 | 3417.77 (3068.32, 3983.21) | 16366.66 (11148.41, 24135.81) | 378.87 |

Note: DALYs: disability-adjusted life years; UI, uncertainty interval

**Supplementary Table S19**. Average annual percentage changes of DALY rate by region and age from 2010 to 2019.

|  | 70-74 years | 75-79 years | 80-84 years | 85-89 years | 90-94 years | 95+ years |
| --- | --- | --- | --- | --- | --- | --- |
| **Global** | 2.390 (1.488, 3.301) | 2.491 (2.045, 2.939) | 5.261 (4.681, 5.843) | 5.410 (4.420, 6.409) | 4.849 (4.152, 5.551) | 4.182 (3.416, 4.954) |
| Low SDI | -1.794 (-2.409, -1.175) | -1.825 (-2.296, -1.352) | 0.944 (0.448, 1.443) | 1.345 (0.839, 1.854) | 1.696 (1.199, 2.195) | 3.085 (2.612, 3.560) |
| Low-middle SDI | 3.008 (2.581, 3.436) | 2.559 (2.049, 3.071) | 6.382 (5.465, 7.306) | 6.276 (5.674, 6.881) | 5.996 (5.218, 6.779) | 5.461 (4.914, 6.010) |
| Middle SDI | 9.872 (9.473, 10.272) | 7.619 (6.791, 8.454) | 10.086 (9.442, 10.735) | 9.969 (9.359, 10.582) | 9.550 (8.773, 10.332) | 8.237 (6.958, 9.530) |
| High-middle SDI | 7.589 (6.754, 8.430) | 4.972 (4.480, 5.467) | 6.069 (4.333, 7.833) | 4.778 (3.213, 6.367) | 4.391 (3.104, 5.694) | 3.368 (2.349, 4.398) |
| High SDI | 2.789 (1.366, 4.232) | 1.979 (0.790, 3.182) | 1.609 (0.850, 2.374) | 1.943 (1.079, 2.815) | 2.953 (2.115, 3.798) | 1.307 (0.134, 2.494) |
| Andean Latin America | 3.947 (3.114, 4.787) | 0.093 (-1.038, 1.237) | 2.974 (0.922, 5.068) | 3.737 (1.760, 5.752) | 4.366 (2.283, 6.491) | 5.126 (2.882, 7.419) |
| Australasia | 0.537 (-0.187, 1.267) | -0.886 (-1.575, -0.192) | -1.417 (-3.574, 0.788) | 0.937 (-0.654, 2.554) | 2.059 (0.288, 3.861) | 2.944 (-1.857, 7.979) |
| Caribbean | 1.313 (0.598, 2.033) | 1.729 (0.468, 3.006) | 3.226 (2.248, 4.213) | 2.786 (2.049, 3.529) | 2.472 (1.951, 2.995) | -0.496 (-1.201, 0.213) |
| Central Asia | 9.919 (8.716, 11.135) | 3.771 (2.965, 4.583) | 3.105 (2.359, 3.856) | 4.844 (3.472, 6.234) | 4.718 (3.118, 6.344) | 4.375 (3.155, 5.609) |
| Central Europe | 6.135 (5.296, 6.981) | 4.698 (3.864, 5.538) | 5.454 (3.860, 7.072) | 6.335 (5.126, 7.559) | 7.799 (6.878, 8.728) | 6.479 (4.460, 8.538) |
| Central Latin America | 6.059 (4.988, 7.141) | 3.596 (2.561, 4.640) | 3.400 (2.155, 4.659) | 3.089 (1.820, 4.375) | 2.821 (2.140, 3.506) | 1.757 (0.860, 2.663) |
| Central Sub-Saharan Africa | -0.998 (-1.484, -0.510) | -0.717 (-1.199, -0.232) | 1.690 (1.171, 2.212) | 2.363 (1.823, 2.906) | 2.597 (2.192, 3.003) | 3.210 (2.854, 3.568) |
| East Asia | 8.922 (8.122, 9.728) | 5.460 (4.191, 6.743) | 9.027 (6.933, 11.163) | 8.905 (5.363, 12.566) | 8.013 (3.402, 12.829) | 6.967 (4.452, 9.544) |
| Eastern Europe | 8.162 (7.165, 9.168) | 6.245 (5.353, 7.145) | 6.607 (5.417, 7.811) | 6.958 (6.334, 7.585) | 8.237 (7.565, 8.914) | 6.915 (6.339, 7.493) |
| Eastern Sub-Saharan Africa | -1.130 (-1.516, -0.743) | -1.264 (-1.668, -0.859) | 1.253 (0.556, 1.954) | 1.753 (1.129, 2.380) | 1.911 (1.288, 2.538) | 2.941 (2.353, 3.533) |
| High-income Asia Pacific | 9.302 (8.210, 10.405) | 8.963 (8.212, 9.720) | 8.595 (7.078, 10.133) | 9.463 (8.198, 10.742) | 12.233 (11.355, 13.117) | 10.015 (8.703, 11.343) |
| High-income North America | 2.682 (1.335, 4.046) | 1.844 (0.833, 2.864) | 1.625 (0.382, 2.883) | 2.327 (0.786, 3.890) | 3.222 (2.243, 4.210) | 1.962 (0.217, 3.738) |
| North Africa and Middle East | 7.401 (6.880, 7.924) | 3.991 (3.482, 4.503) | 7.613 (6.948, 8.282) | 7.792 (7.422, 8.162) | 8.483 (8.118, 8.850) | 9.268 (8.832, 9.706) |
| Oceania | 20.502 (18.598, 22.437) | 13.054 (11.666, 14.460) | 14.370 (12.679, 16.086) | 13.660 (12.541, 14.790) | 13.969 (12.865, 15.084) | 15.664 (14.353, 16.990) |
| South Asia | 10.927 (10.107, 11.753) | 2.162 (1.525, 2.803) | 1.496 (0.388, 2.616) | 1.177 (0.590, 1.768) | 0.342 (-0.219, 0.907) | -0.110 (-1.131, 0.921) |
| Southeast Asia | 20.084 (17.961, 22.246) | 7.560 (6.734, 8.392) | 5.379 (4.637, 6.126) | 5.657 (4.588, 6.737) | 5.545 (4.834, 6.262) | 5.873 (4.379, 7.389) |
| Southern Latin America | 5.722 (4.166, 7.301) | 2.717 (1.040, 4.422) | 2.113 (1.363, 2.869) | 1.201 (0.427, 1.980) | 3.224 (2.396, 4.058) | 2.990 (1.788, 4.206) |
| Southern Sub-Saharan Africa | 7.901 (6.282, 9.545) | 8.796 (7.241, 10.374) | 15.265 (13.453, 17.106) | 17.031 (15.516, 18.565) | 17.676 (16.213, 19.158) | 18.888 (17.464, 20.329) |
| Tropical Latin America | 13.217 (3.457, 23.898) | 10.315 (5.422, 15.435) | 8.428 (7.433, 9.433) | 5.039 (2.608, 7.528) | 3.073 (1.955, 4.203) | 3.239 (2.609, 3.873) |
| Western Europe | 4.404 (2.614, 6.225) | 2.970 (2.069, 3.878) | 2.743 (1.210, 4.299) | 1.879 (1.111, 2.652) | 3.644 (3.015, 4.277) | 2.405 (1.727, 3.087) |
| Western Sub-Saharan Africa | 3.069 (2.557, 3.584) | 2.907 (2.413, 3.403) | 6.895 (5.814, 7.986) | 7.552 (6.912, 8.196) | 8.292 (7.807, 8.780) | 9.125 (8.615, 9.637) |
| African Region | 1.573 (1.028, 2.121) | 1.492 (0.936, 2.051) | 4.418 (4.049, 4.788) | 5.319 (4.982, 5.656) | 6.090 (5.686, 6.497) | 7.102 (6.694, 7.511) |
| Eastern Mediterranean Region | 8.330 (7.653, 9.011) | 2.699 (2.356, 3.044) | 4.435 (3.779, 5.095) | 4.439 (3.557, 5.329) | 4.634 (4.017, 5.254) | 5.252 (4.806, 5.700) |
| European Region | 5.201 (3.922, 6.496) | 3.411 (2.618, 4.210) | 3.089 (1.594, 4.606) | 2.412 (1.666, 3.163) | 4.031 (2.900, 5.175) | 3.173 (2.565, 3.785) |
| Region of the Americas | 3.797 (3.235, 4.362) | 2.863 (2.276, 3.453) | 3.263 (2.658, 3.873) | 3.114 (2.194, 4.042) | 3.617 (3.062, 4.175) | 2.808 (1.329, 4.307) |
| South-East Asia Region | 16.367 (15.760, 16.976) | 6.336 (5.775, 6.901) | 4.376 (3.656, 5.102) | 4.061 (2.955, 5.180) | 3.684 (2.810, 4.565) | 4.103 (2.993, 5.225) |
| Western Pacific Region | 9.221 (8.563, 9.883) | 5.406 (3.942, 6.890) | 8.932 (7.143, 10.750) | 8.755 (7.179, 10.355) | 7.672 (4.630, 10.802) | 6.033 (3.121, 9.028) |
| Low Income | -1.432 (-2.002, -0.858) | -1.630 (-2.410, -0.843) | 0.960 (0.643, 1.277) | 1.634 (1.260, 2.010) | 2.256 (1.827, 2.685) | 3.629 (3.264, 3.996) |
| Lower Middle Income | 3.122 (2.698, 3.548) | 2.969 (2.190, 3.754) | 6.403 (5.772, 7.037) | 6.782 (5.992, 7.577) | 6.707 (6.162, 7.255) | 8.362 (7.923, 8.802) |
| Upper Middle Income | 10.202 (9.912, 10.493) | 7.765 (6.776, 8.763) | 9.910 (9.451, 10.371) | 9.201 (8.630, 9.775) | 8.470 (7.949, 8.994) | 6.274 (5.701, 6.851) |
| High Income | 3.018 (1.779, 4.272) | 2.288 (1.511, 3.071) | 1.599 (0.965, 2.238) | 1.581 (0.851, 2.317) | 2.760 (2.023, 3.503) | 1.271 (-0.379, 2.948) |

Note: data was presented as average annual percentage change and its 95% confidence interval


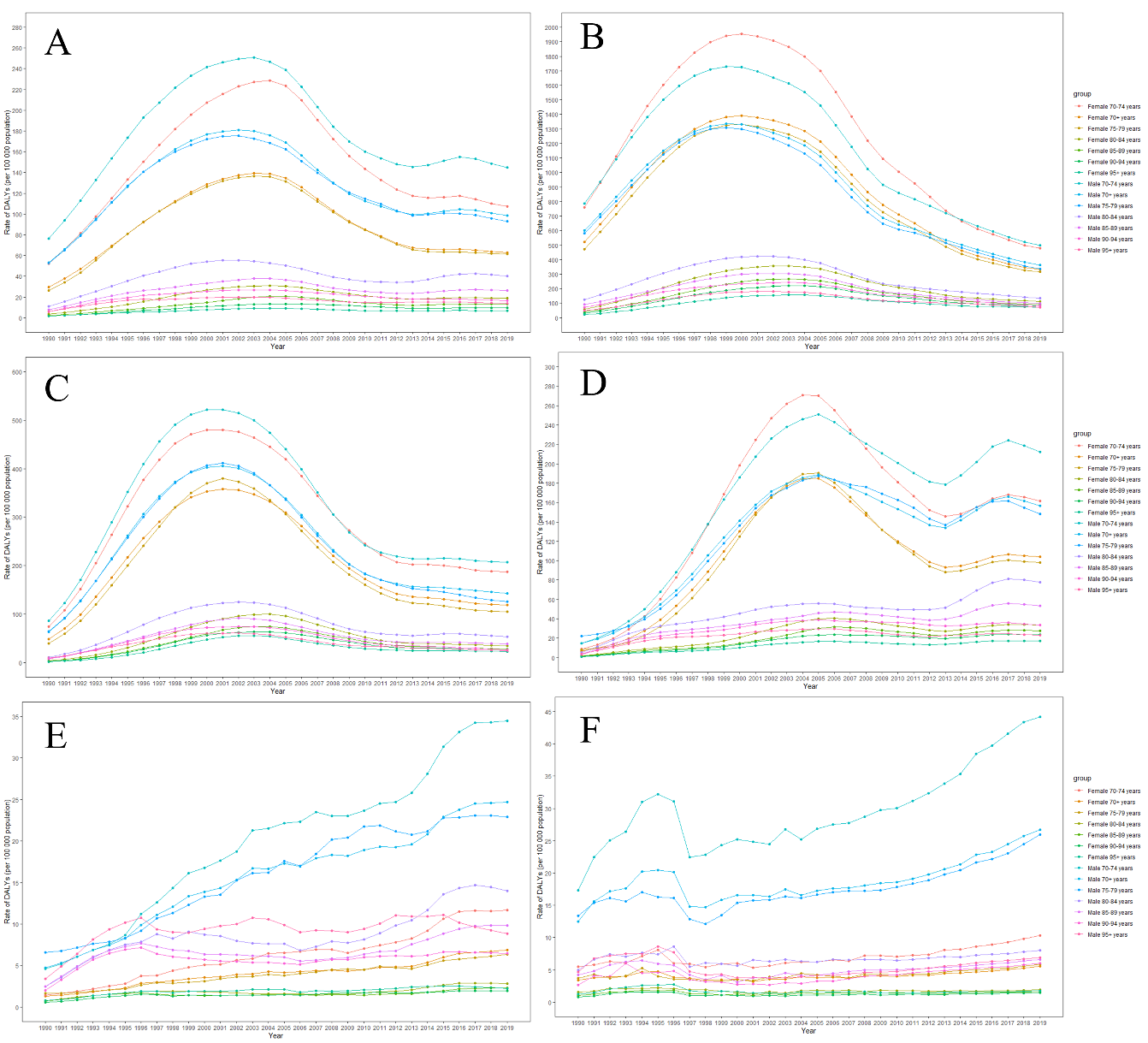


**Supplementary Figure S8**. Sex and age group distribution of HIV/AIDs DALY rate by SDI region from1990 to 2019.

(A) Global; (B) Low SDI; (C) Low-middle SDI; (D) Middle SDI; (E) High-middle SDI; (F) High SDI. SDI: Socio-demographic Index
